# Supplementary material for: Untargeted Fecal Metabolomic Analyses across an Industrialization Gradient Reveal Shared Metabolites and Impact of Industrialization on Fecal Microbiome-Metabolome Interactions
Source: mSystems. 2022 Nov 23;7(6):e00710-22. doi: 10.1128/msystems.00710-22 (PMC9765122; doi:10.1128/msystems.00710-22)
Supplement: TABLE S1 [file msystems.00710-22-s0005.pdf]

SampleID	Population	Industrialization Category	Sex	Age
NO01	Norman	Urban Industrial	M	23
NO02	Norman	Urban Industrial	F	37
NO03	Norman	Urban Industrial	M	40
NO04	Norman	Urban Industrial	M	26
NO05	Norman	Urban Industrial	M	28
NO06	Norman	Urban Industrial	M	28
NO07	Norman	Urban Industrial	F	32
NO08	Norman	Urban Industrial	F	32
NO09	Norman	Urban Industrial	F	34
NO10	Norman	Urban Industrial	M	41
NO11	Norman	Urban Industrial	M	26
NO12	Norman	Urban Industrial	F	27
NO13	Norman	Urban Industrial	M	35
NO19	Norman	Urban Industrial	F	32
NO20	Norman	Urban Industrial	M	26
NO21	Norman	Urban Industrial	M	23
NO22	Norman	Urban Industrial	M	26
NO23	Norman	Urban Industrial	F	26
GU1	Guayabo	Rural Industrial	F	52
GU2	Guayabo	Rural Industrial	F	19
GU4	Guayabo	Rural Industrial	F	40
GU6	Guayabo	Rural Industrial	NA	6
GU7	Guayabo	Rural Industrial	NA	9
GU10	Guayabo	Rural Industrial	F	58
GU11	Guayabo	Rural Industrial	NA	7
GU12	Guayabo	Rural Industrial	NA	16
GU13	Guayabo	Rural Industrial	F	41
GU17	Guayabo	Rural Industrial	F	51
GU19	Guayabo	Rural Industrial	F	24
GU20	Guayabo	Rural Industrial	F	63
1TM	Tambo de Mora	Rural Industrial	F	61
2TM	Tambo de Mora	Rural Industrial	F	40
3TM	Tambo de Mora	Rural Industrial	NA	5
4TM	Tambo de Mora	Rural Industrial	F	40
6TM	Tambo de Mora	Rural Industrial	F	31

10TM	Tambo de Mora	Rural Industrial	NA	8
11TM	Tambo de Mora	Rural Industrial	M	39
14TM	Tambo de Mora	Rural Industrial	F	77
16TM	Tambo de Mora	Rural Industrial	F	38
17TM	Tambo de Mora	Rural Industrial	NA	7
18TM	Tambo de Mora	Rural Industrial	NA	13
26TM	Tambo de Mora	Rural Industrial	F	36
27TM	Tambo de Mora	Rural Industrial	NA	13
31TM	Tambo de Mora	Rural Industrial	NA	28
TM10_01	Boulkiemdé	Rural Traditional	M	55
TM13_01	Boulkiemdé	Rural Traditional	M	53
TM23_02	Boulkiemdé	Rural Traditional	F	51
TM01_01	Boulkiemdé	Rural Traditional	M	55
TM09_02	Boulkiemdé	Rural Traditional	F	32
TM11_04	Boulkiemdé	Rural Traditional	F	40
TM17_02	Boulkiemdé	Rural Traditional	F	35
TM20_03	Boulkiemdé	Rural Traditional	F	37
TM22_03	Boulkiemdé	Rural Traditional	M	29
TM25_03	Boulkiemdé	Rural Traditional	F	38
TM29_01	Boulkiemdé	Rural Traditional	M	73
HCO01	Tunapuco	Rural Traditional	F	36
HCO03	Tunapuco	Rural Traditional	M	6
HCO09	Tunapuco	Rural Traditional	NA	13
HCO10	Tunapuco	Rural Traditional	M	10
HCO11	Tunapuco	Rural Traditional	M	36
HCO12	Tunapuco	Rural Traditional	F	35
HCO13	Tunapuco	Rural Traditional	F	9
HCO14	Tunapuco	Rural Traditional	F	34
HCO15	Tunapuco	Rural Traditional	F	63
HCO16	Tunapuco	Rural Traditional	NA	11
HCO17	Tunapuco	Rural Traditional	F	7
HCO18	Tunapuco	Rural Traditional	M	11
HCO21	Tunapuco	Rural Traditional	M	10
HCO41	Tunapuco	Rural Traditional	NA	54
HCO53	Tunapuco	Rural Traditional	F	44
HCO61	Tunapuco	Rural Traditional	F	20
HCO63	Tunapuco	Rural Traditional	F	6
HCO66	Tunapuco	Rural Traditional	M	11

HCO67	Tunapuco	Rural Traditional	F	26
HCO68	Tunapuco	Rural Traditional	M	7
HCO69	Tunapuco	Rural Traditional	NA	9
HCO70	Tunapuco	Rural Traditional	F	40
HCO72	Tunapuco	Rural Traditional	F	5
HCO74	Tunapuco	Rural Traditional	F	36
SM01	Matses	Isolated Traditional	M	30
SM02	Matses	Isolated Traditional	F	25
SM03	Matses	Isolated Traditional	M	10
SM10	Matses	Isolated Traditional	F	6
SM23	Matses	Isolated Traditional	M	7
SM28	Matses	Isolated Traditional	F	52
SM29	Matses	Isolated Traditional	F	50
SM33	Matses	Isolated Traditional	F	5
SM37	Matses	Isolated Traditional	M	12
SM39	Matses	Isolated Traditional	F	40
SM41	Matses	Isolated Traditional	M	6
