# Supplementary figures and images for: Untargeted Fecal Metabolomic Analyses across an Industrialization Gradient Reveal Shared Metabolites and Impact of Industrialization on Fecal Microbiome-Metabolome Interactions
Source: mSystems. 2022 Nov 23;7(6):e00710-22. doi: 10.1128/msystems.00710-22 (PMC9765122; doi:10.1128/msystems.00710-22)

**a**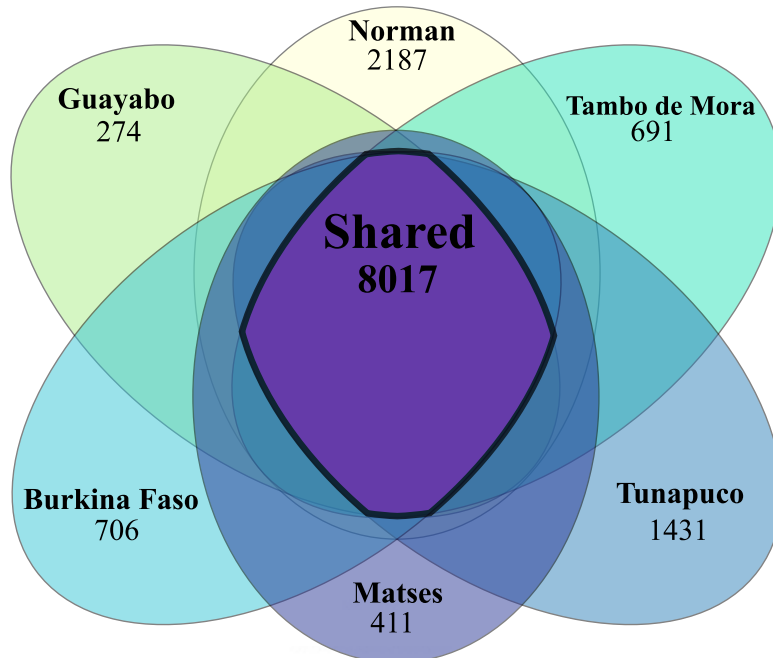**b**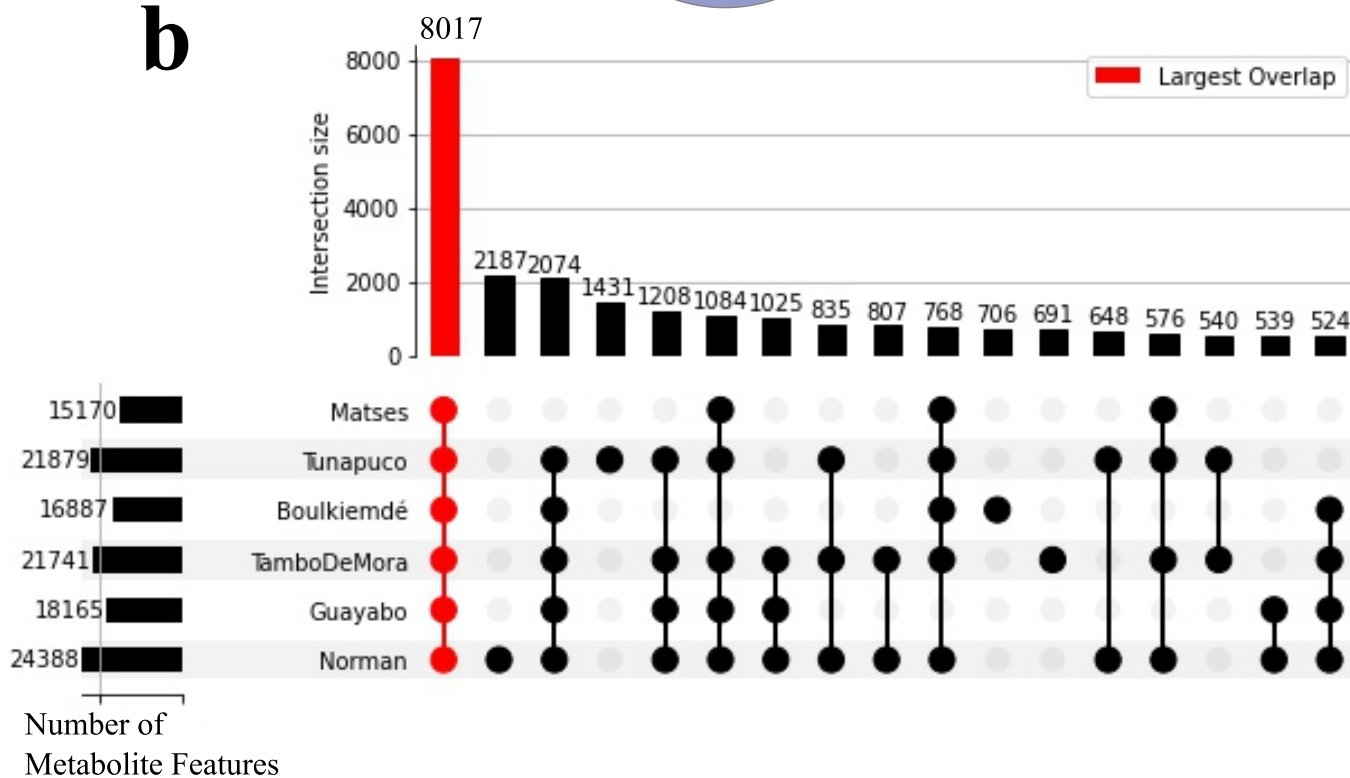**c**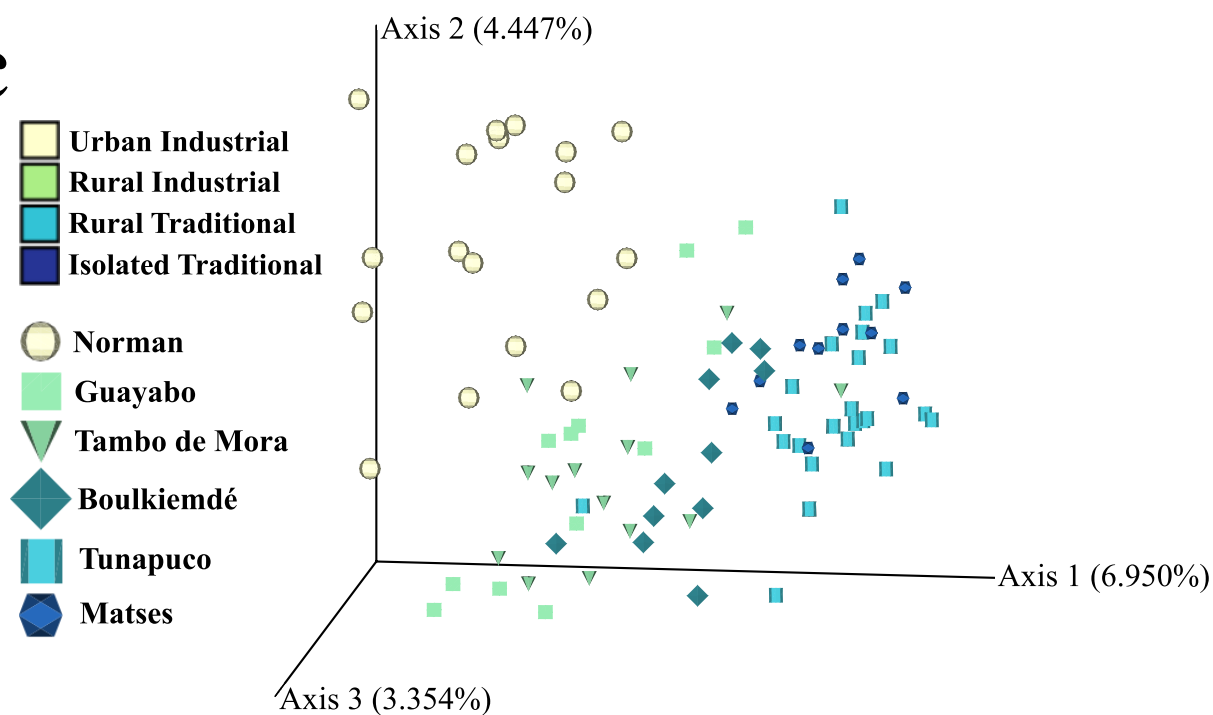

Supplement: FIG S1 [file msystems.00710-22-s0001.pdf]

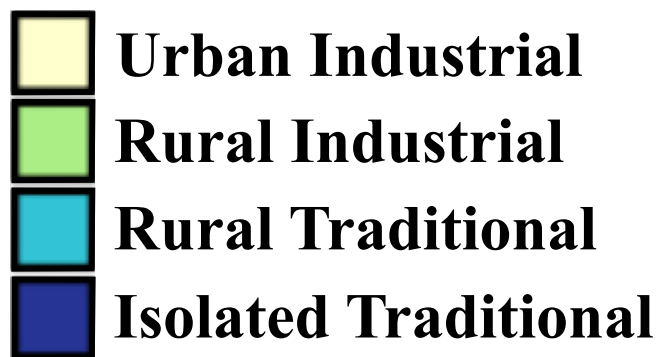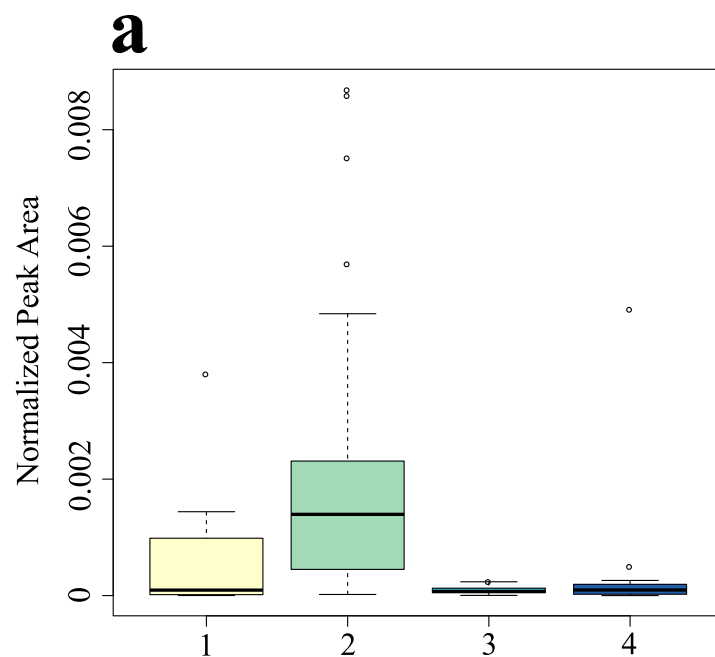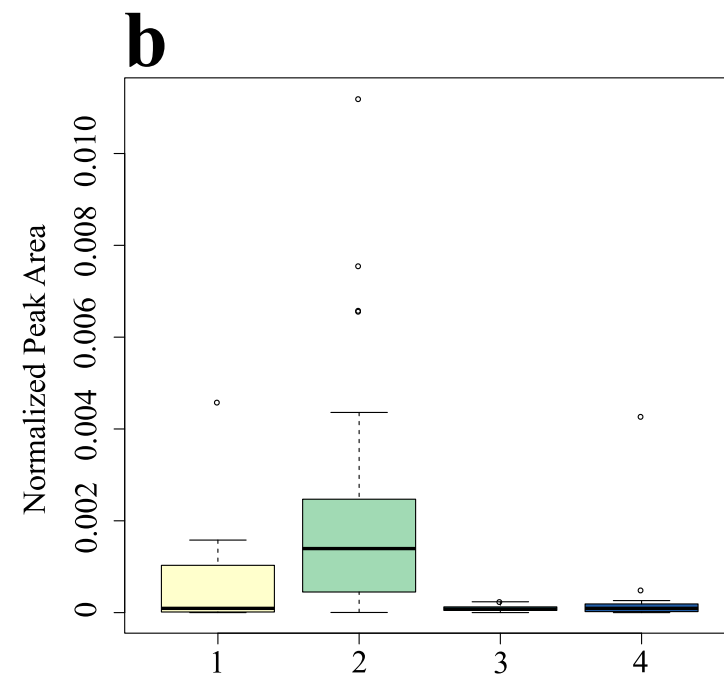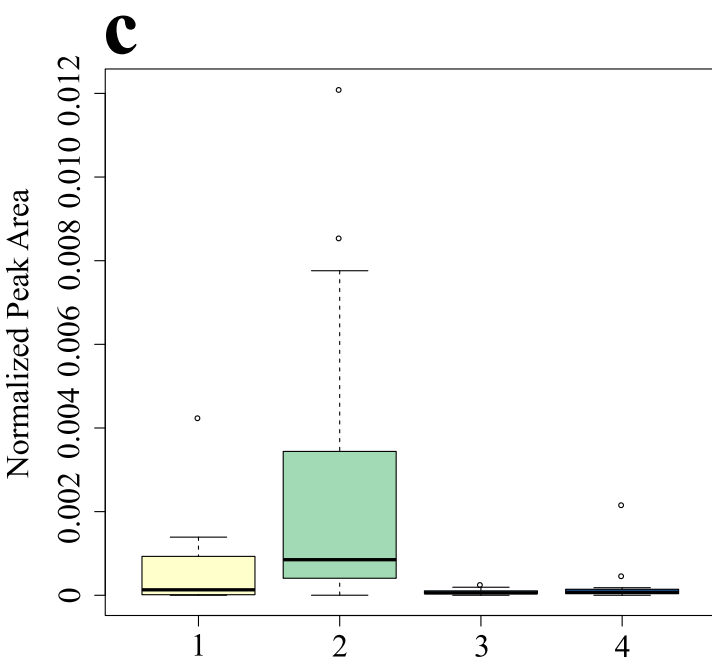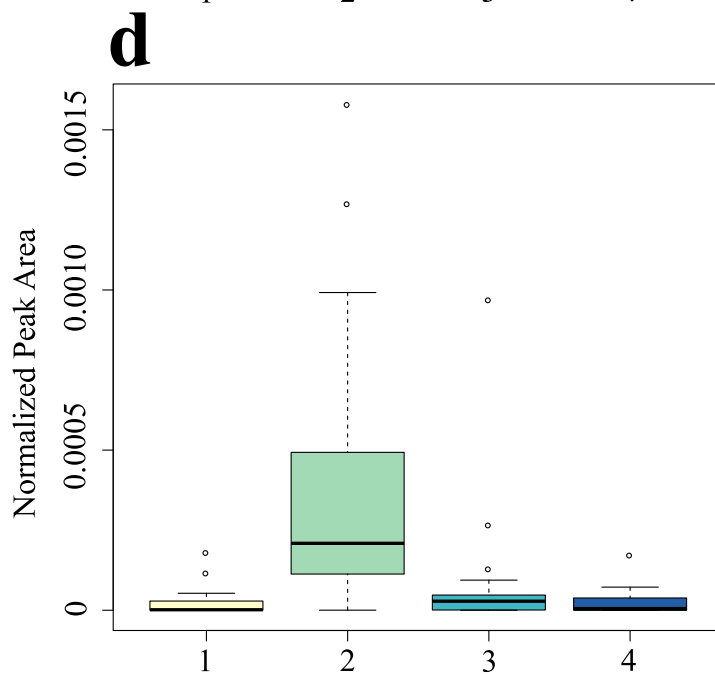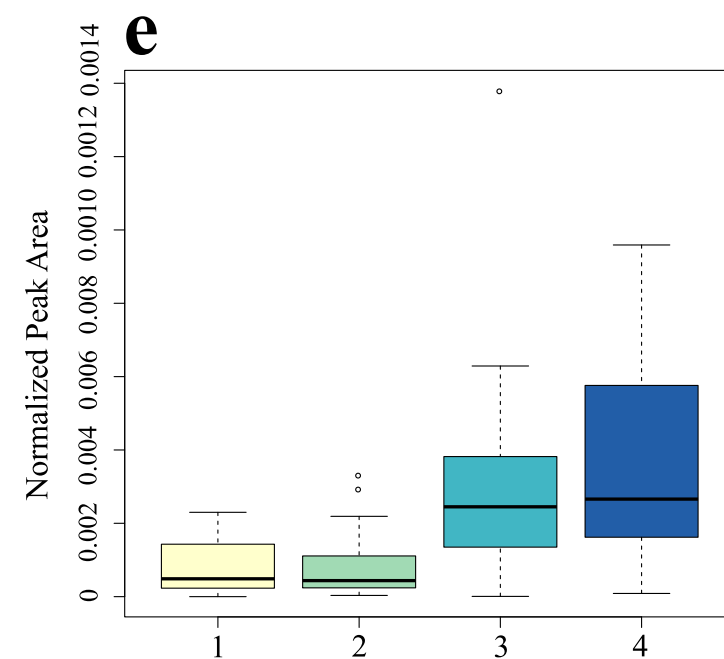

**f**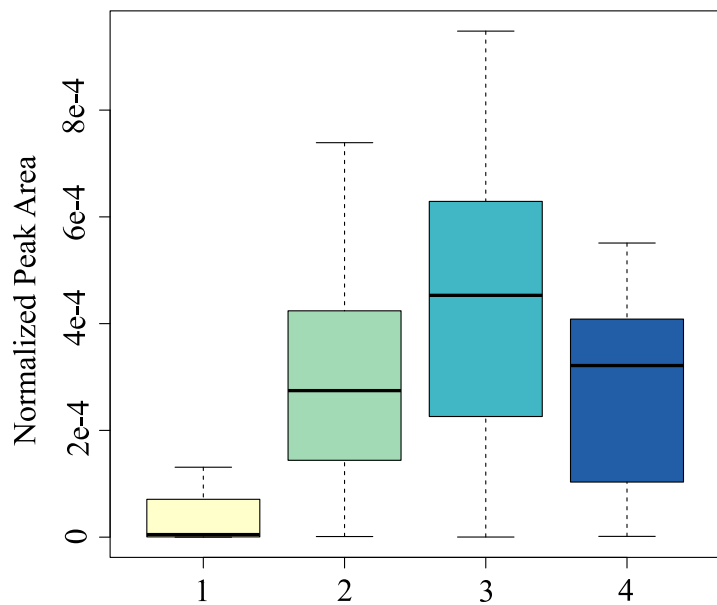**g**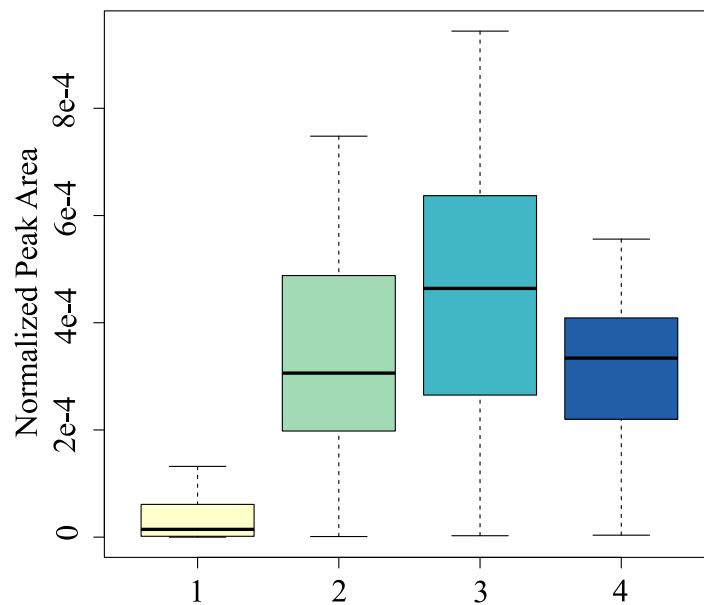**h**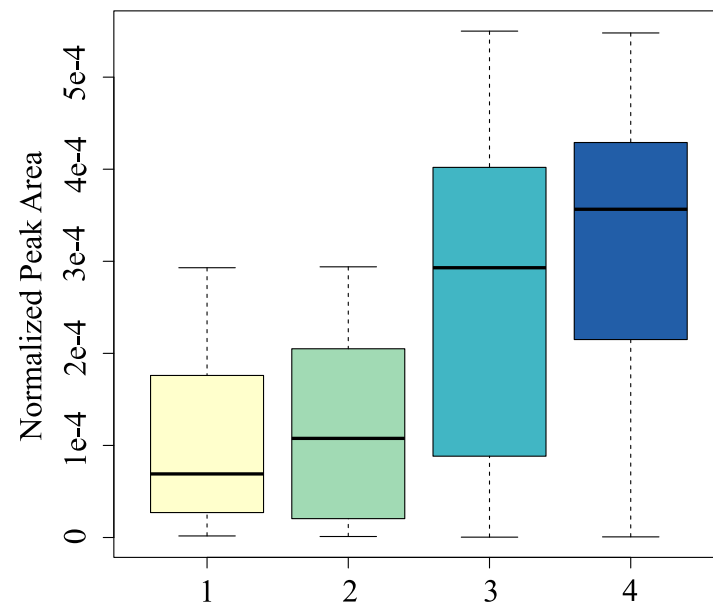**i**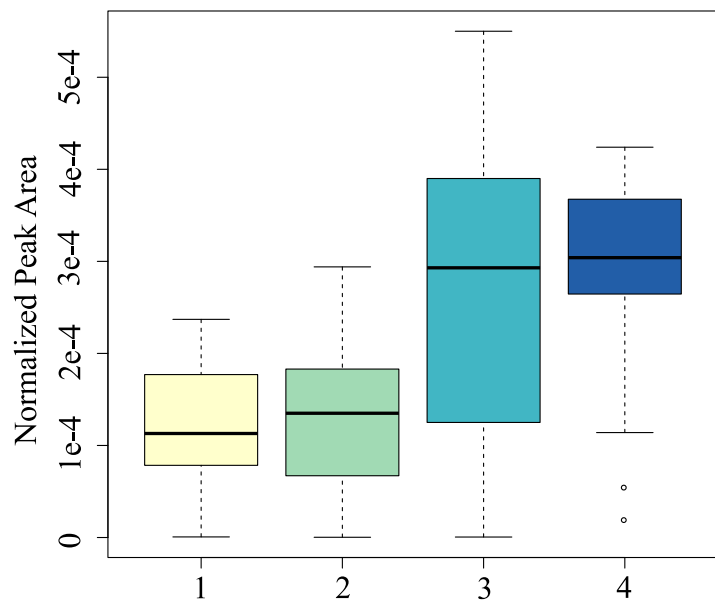**j**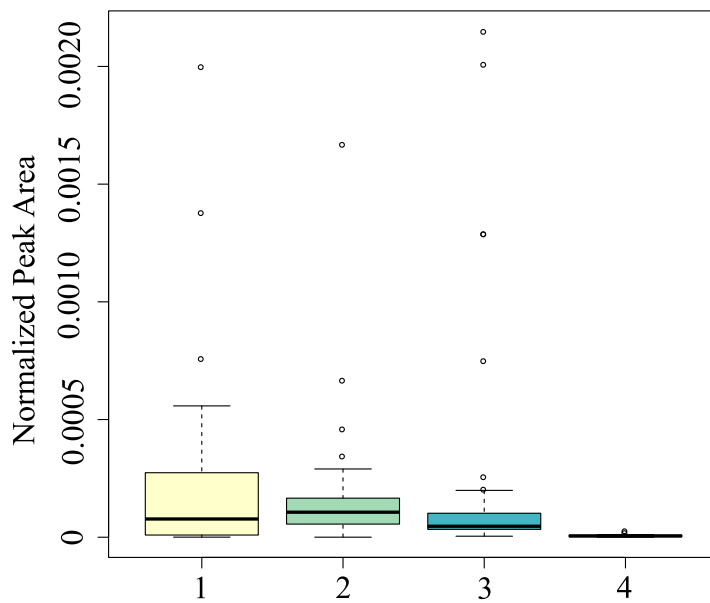**k**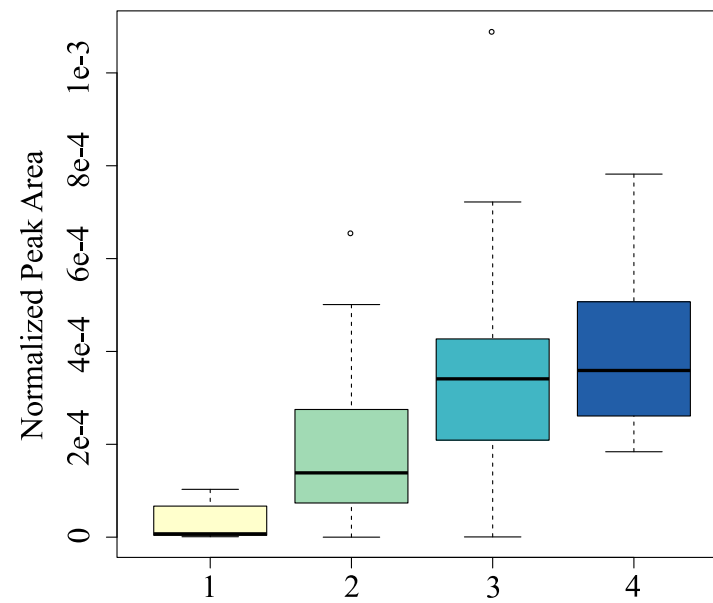

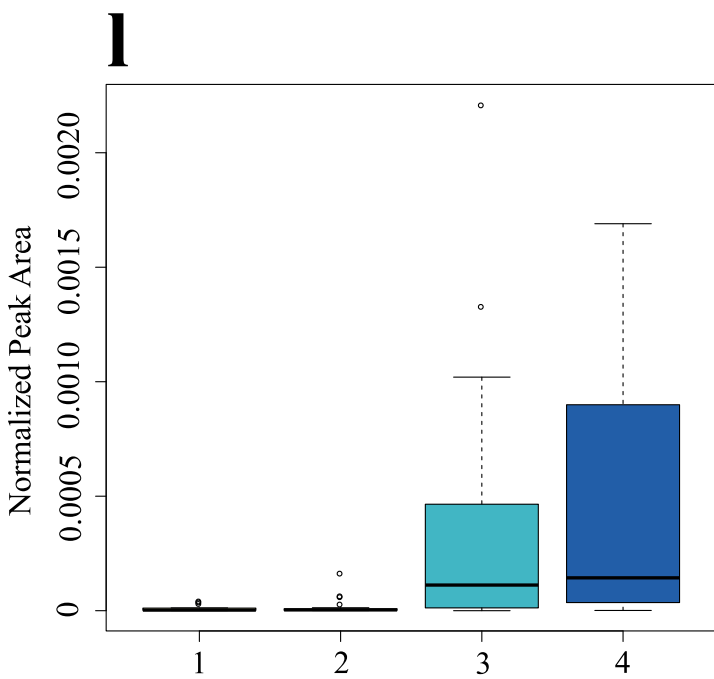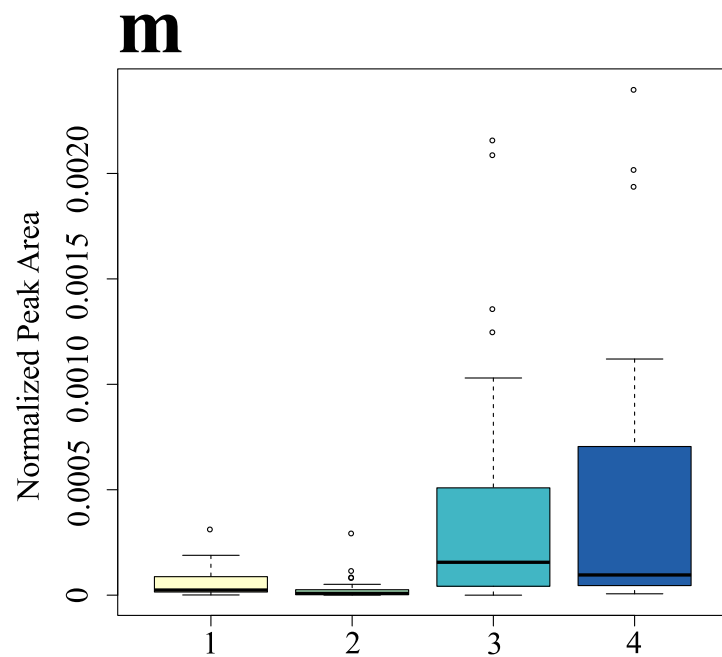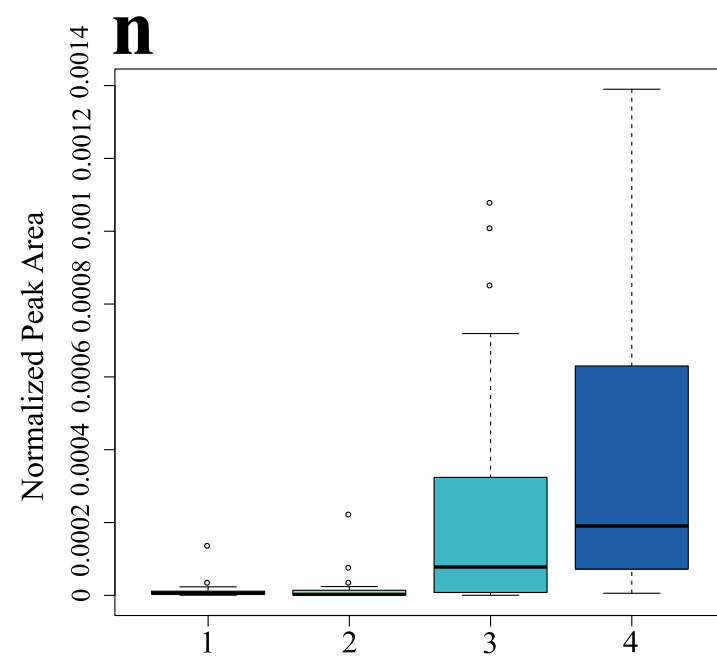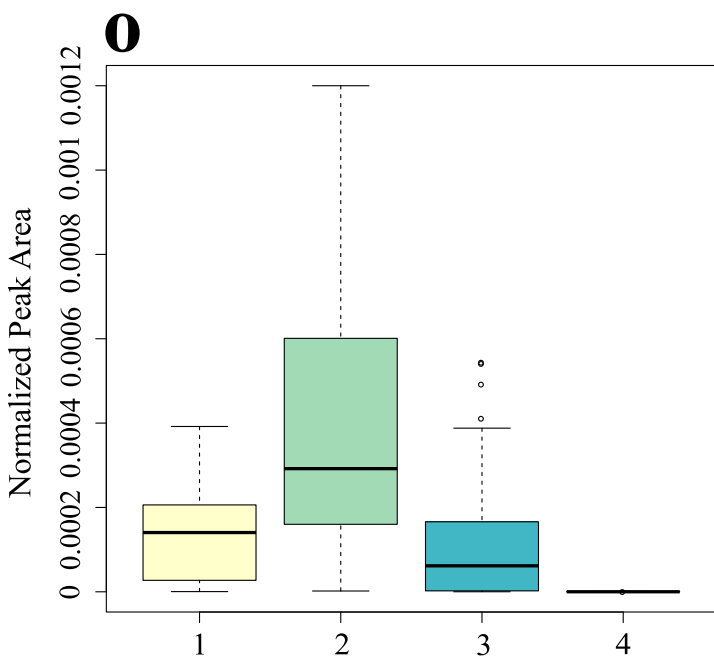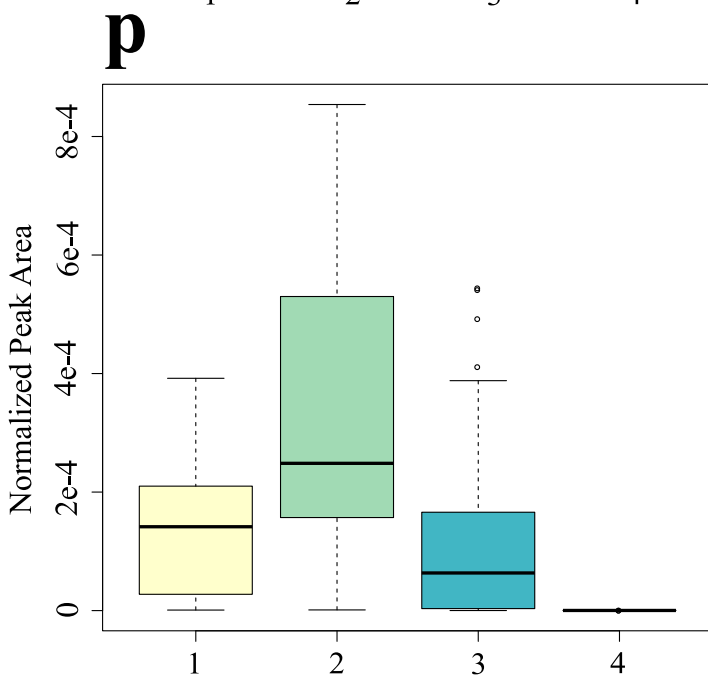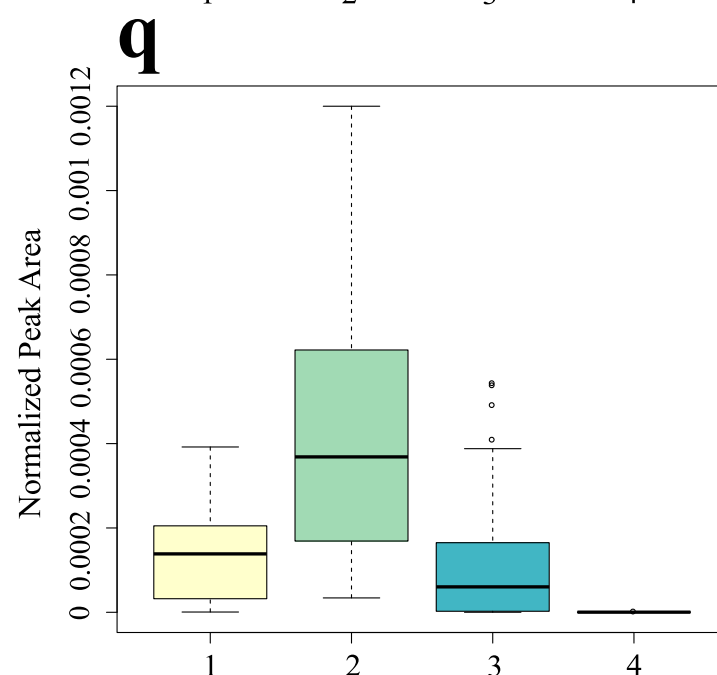

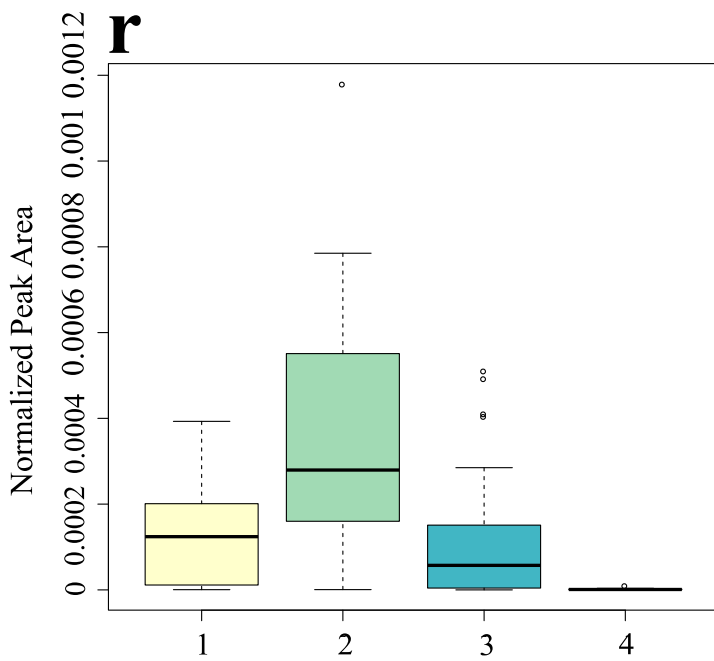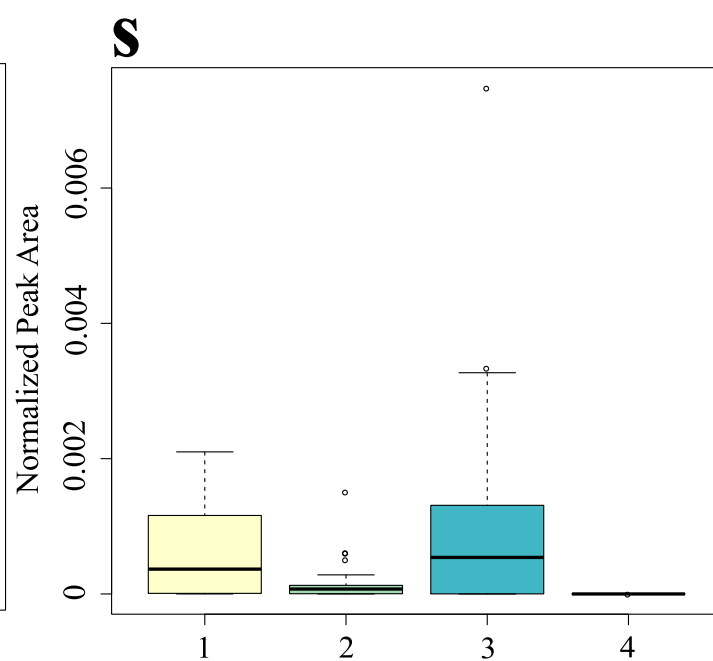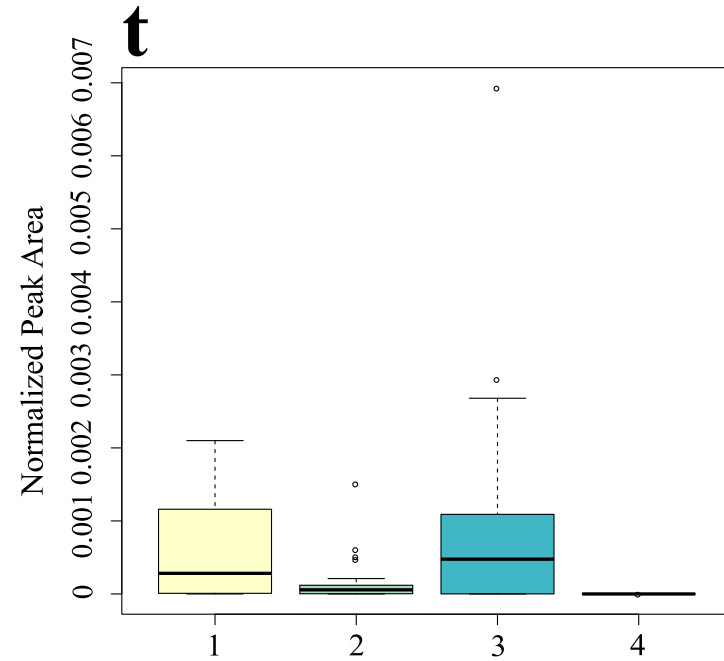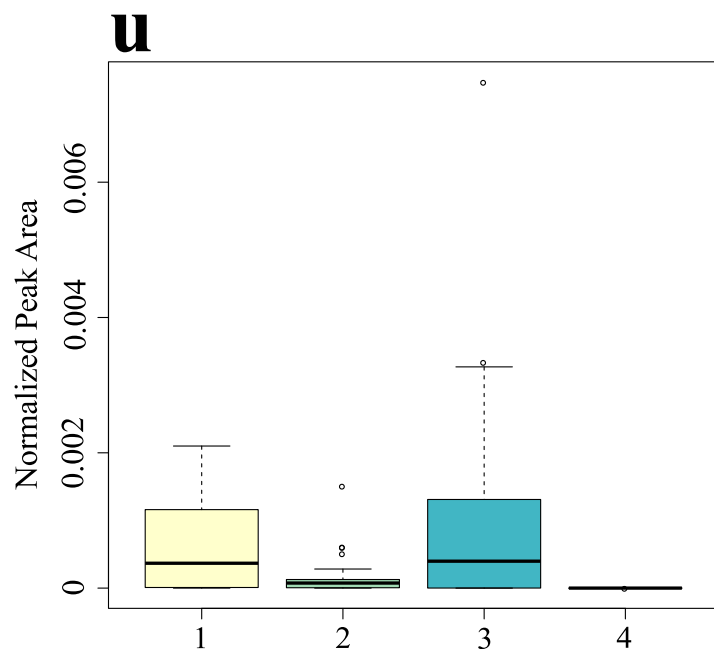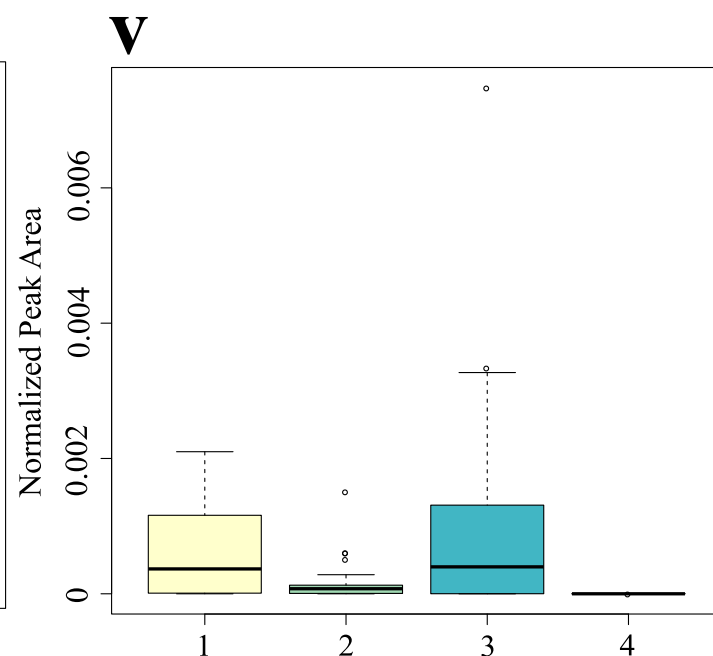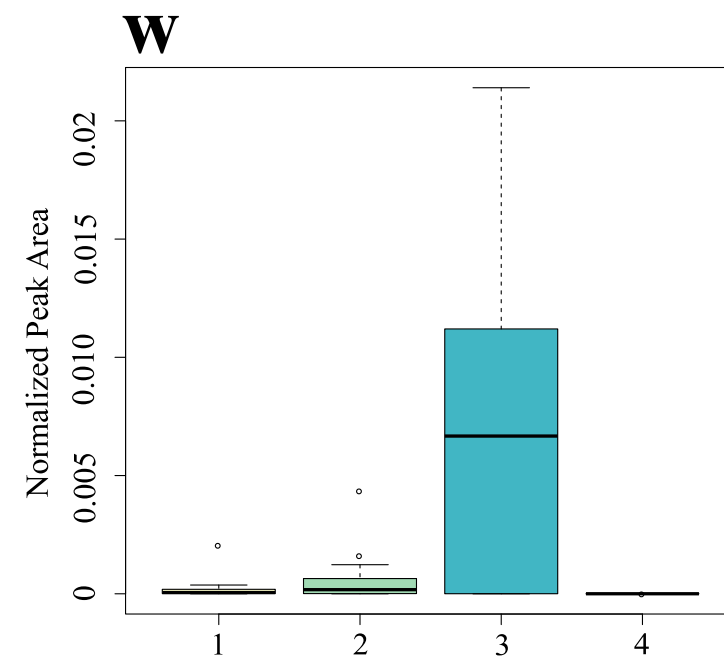

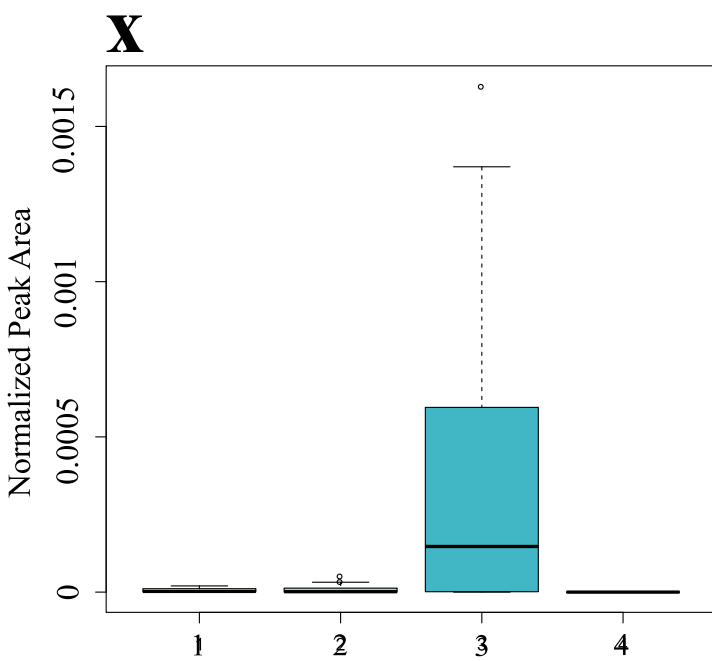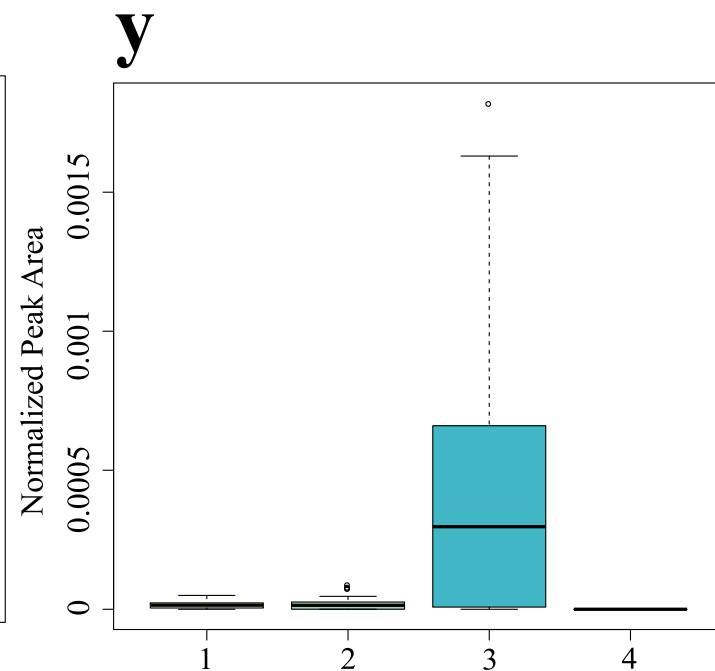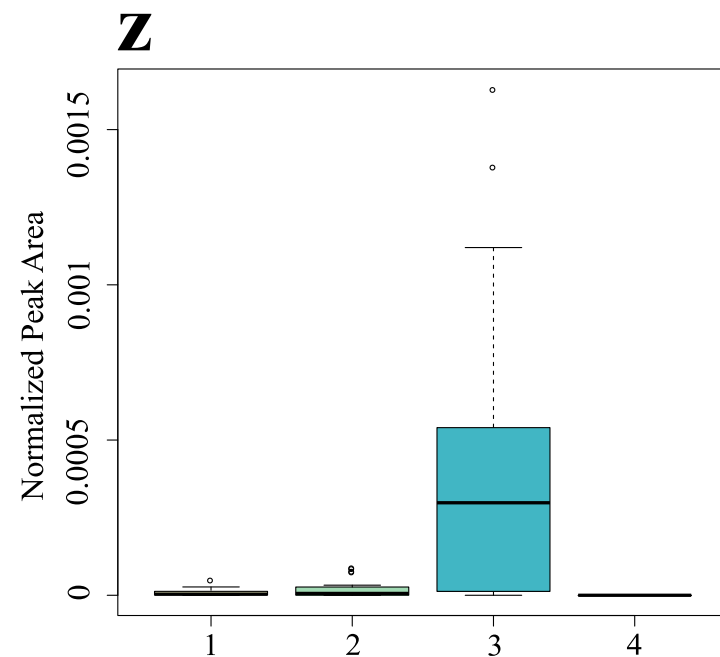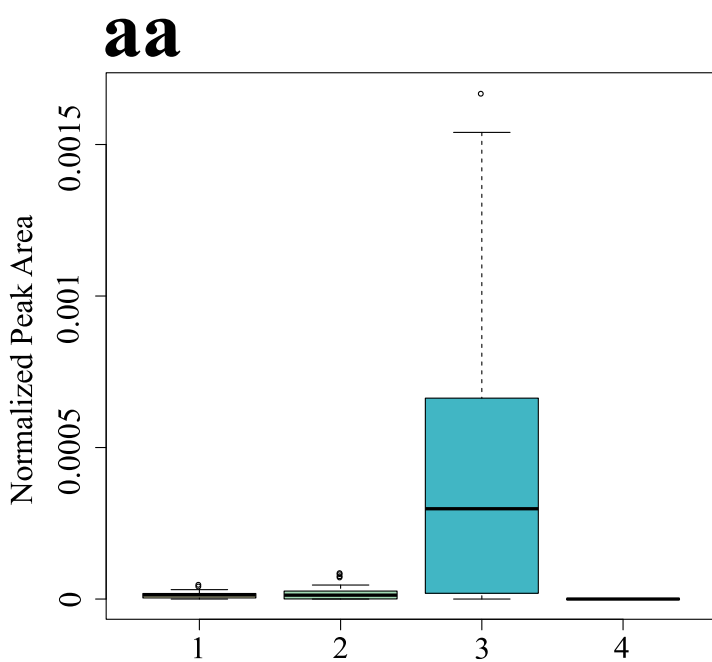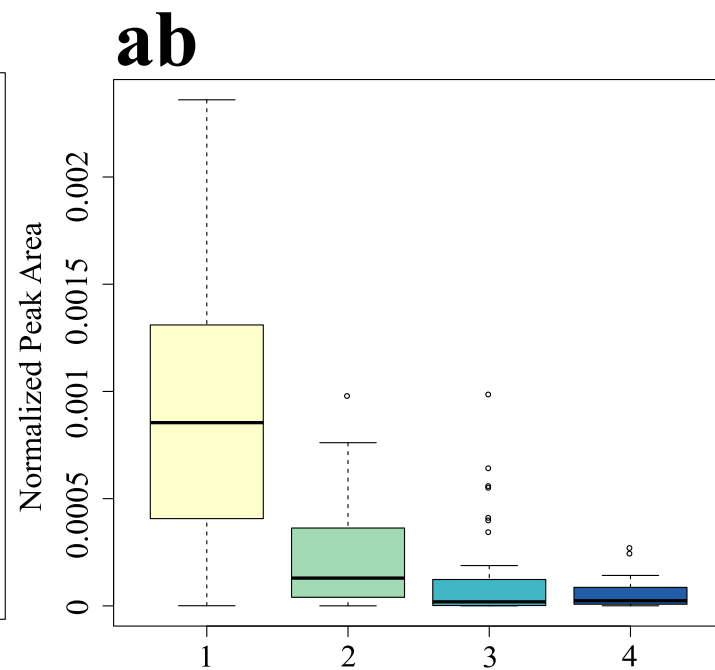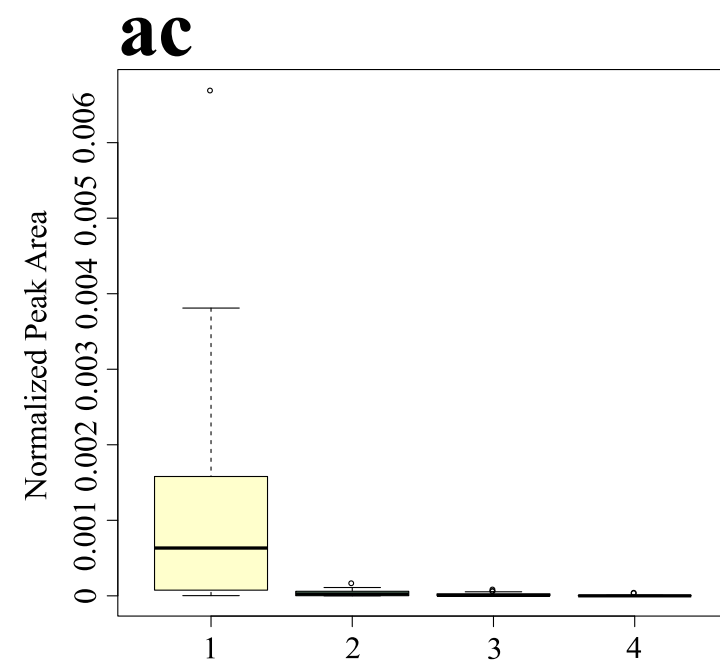

**ad**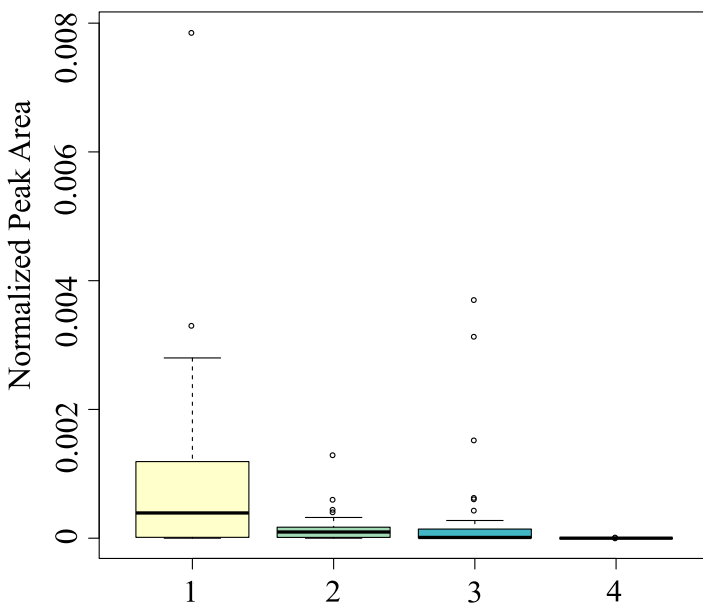**ae**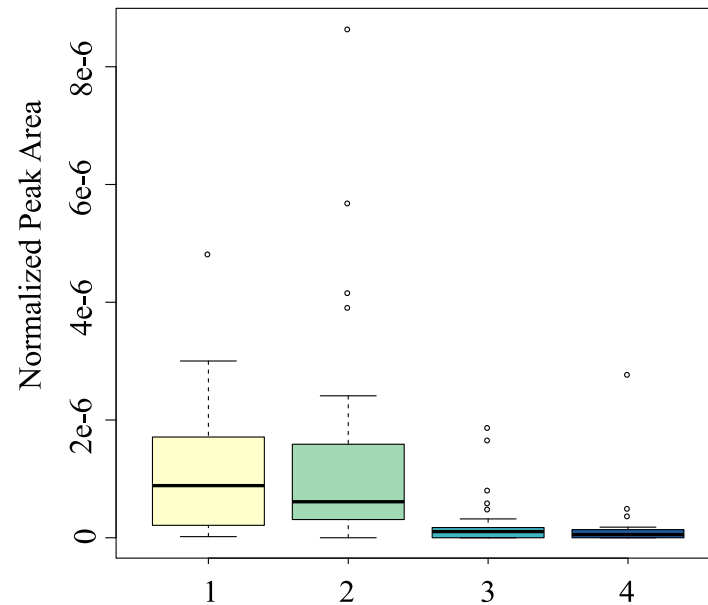**af**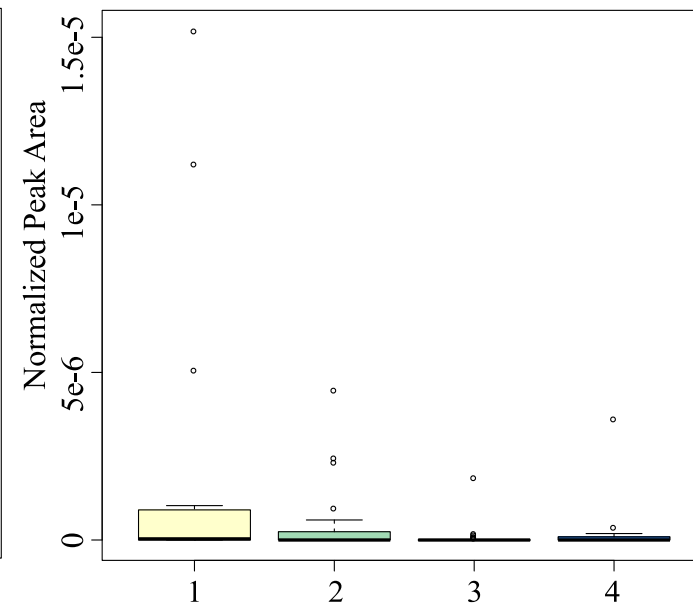**ag**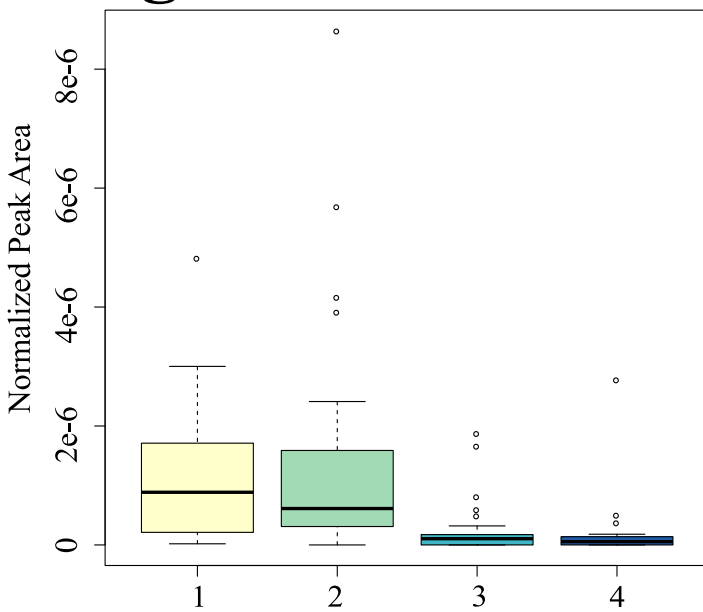**ah**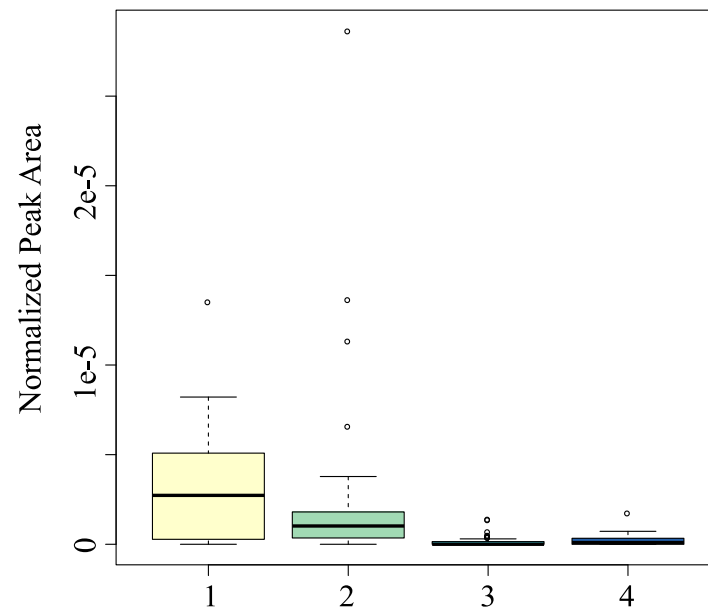**ai**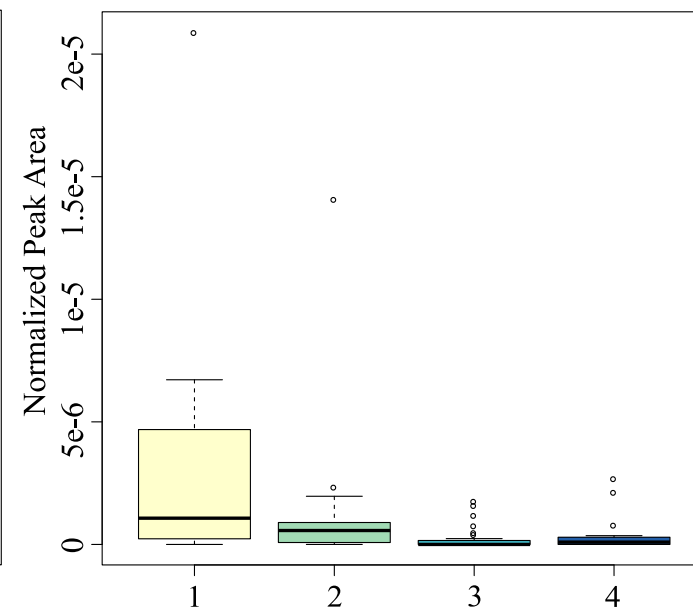

**aj**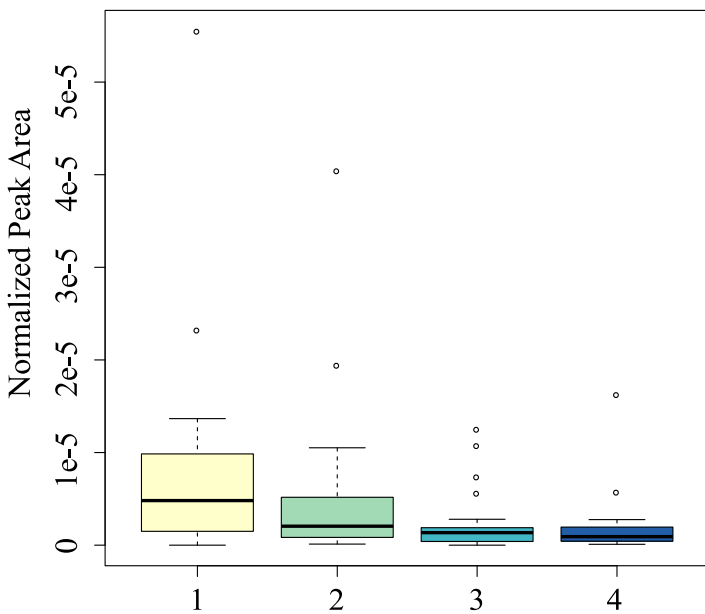**ak**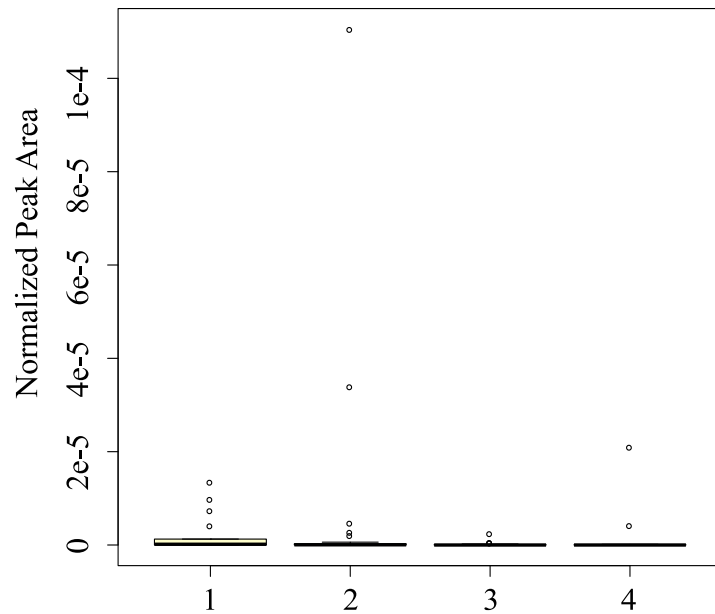**al**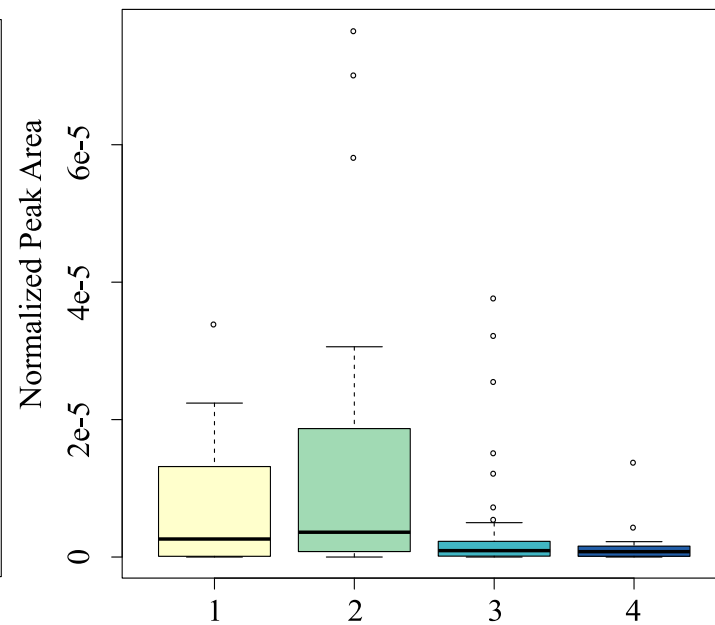**am**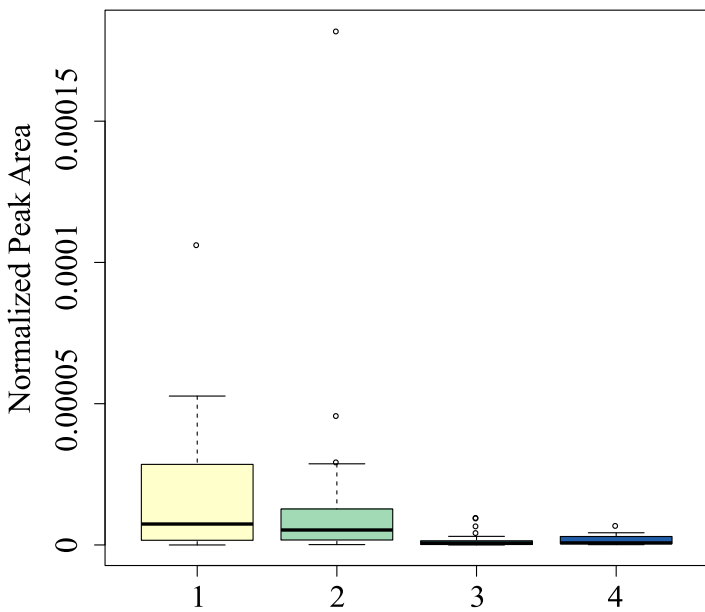**an**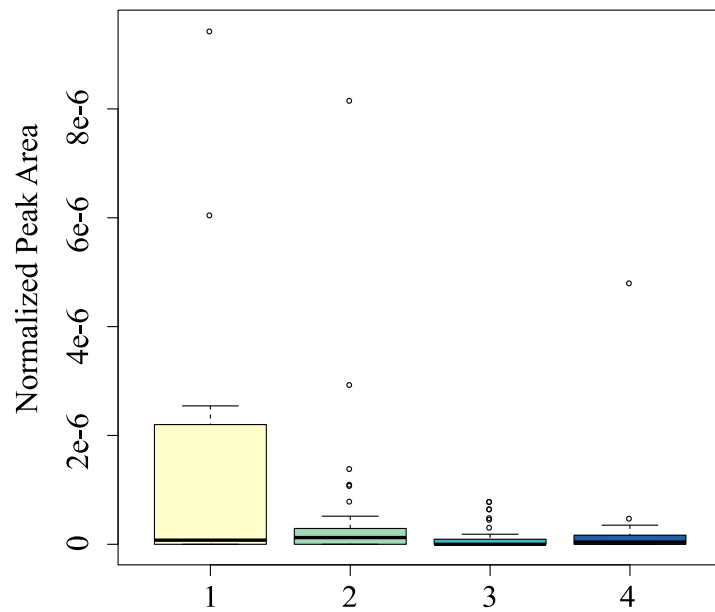**ao**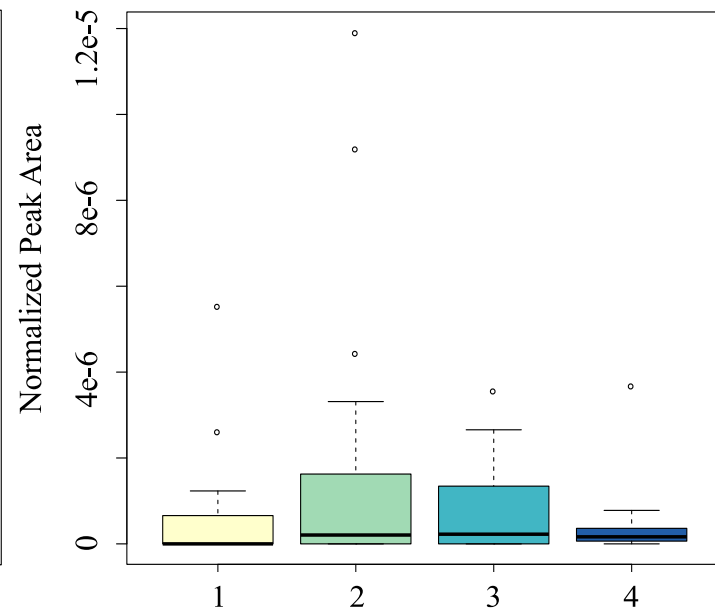

**ap**

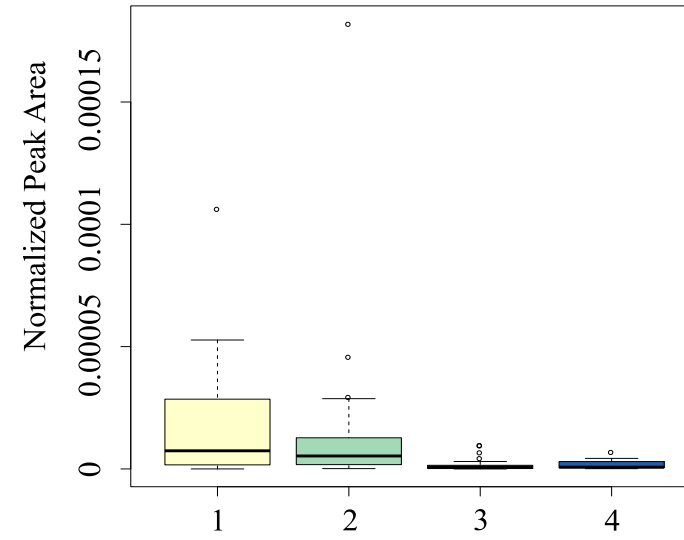

Supplement: FIG S2 [file msystems.00710-22-s0002.pdf]

**a**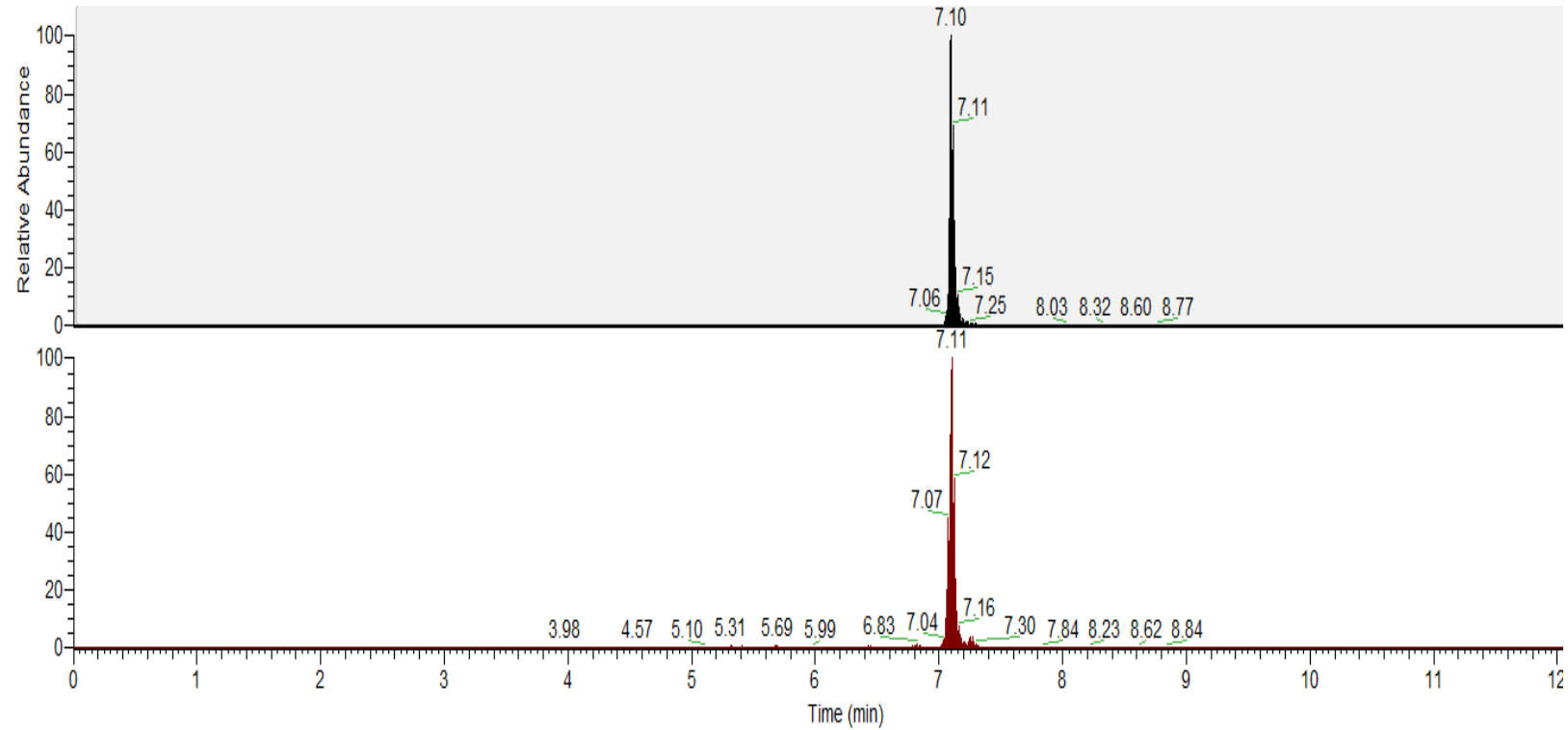**b**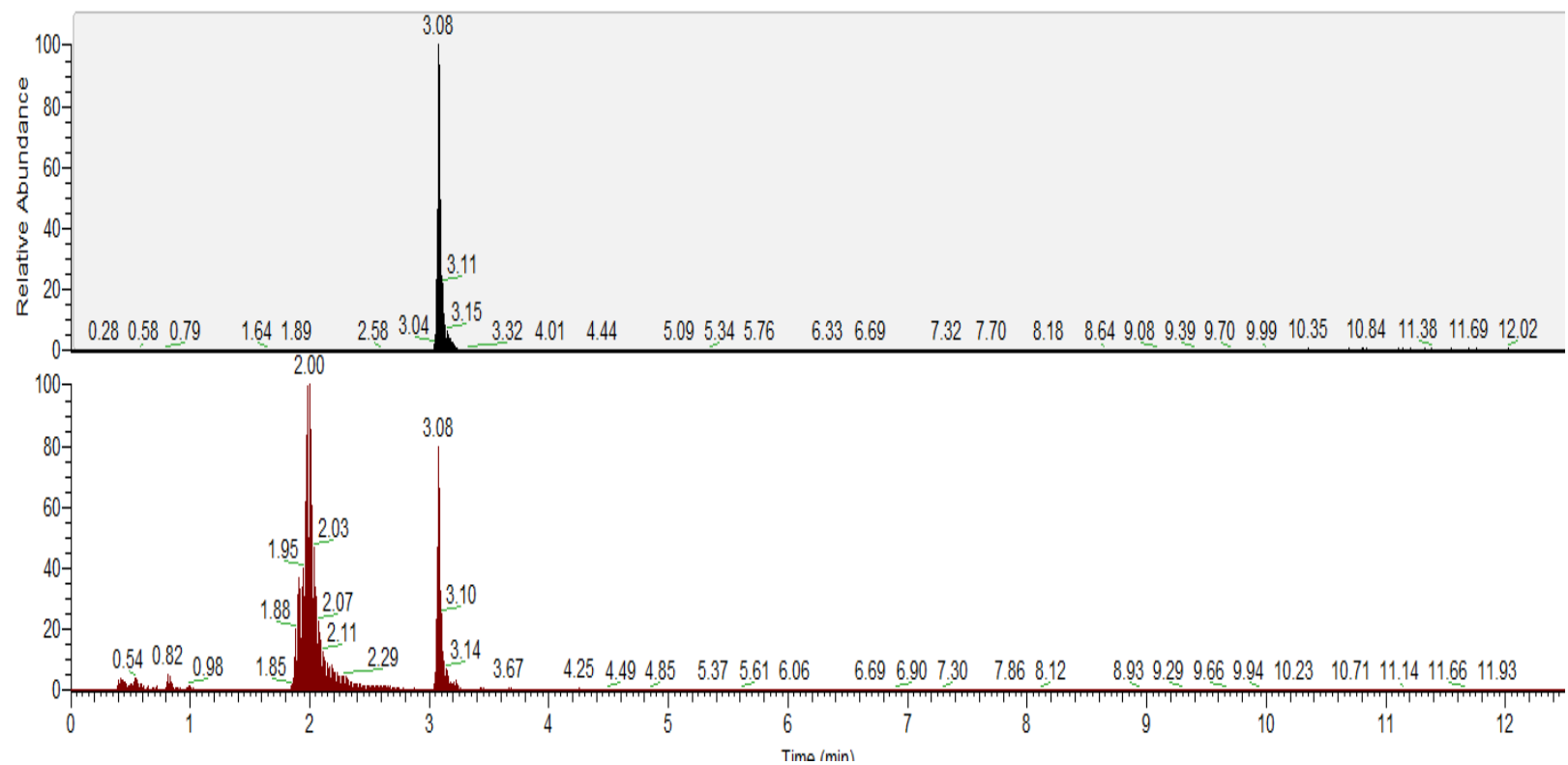

**c**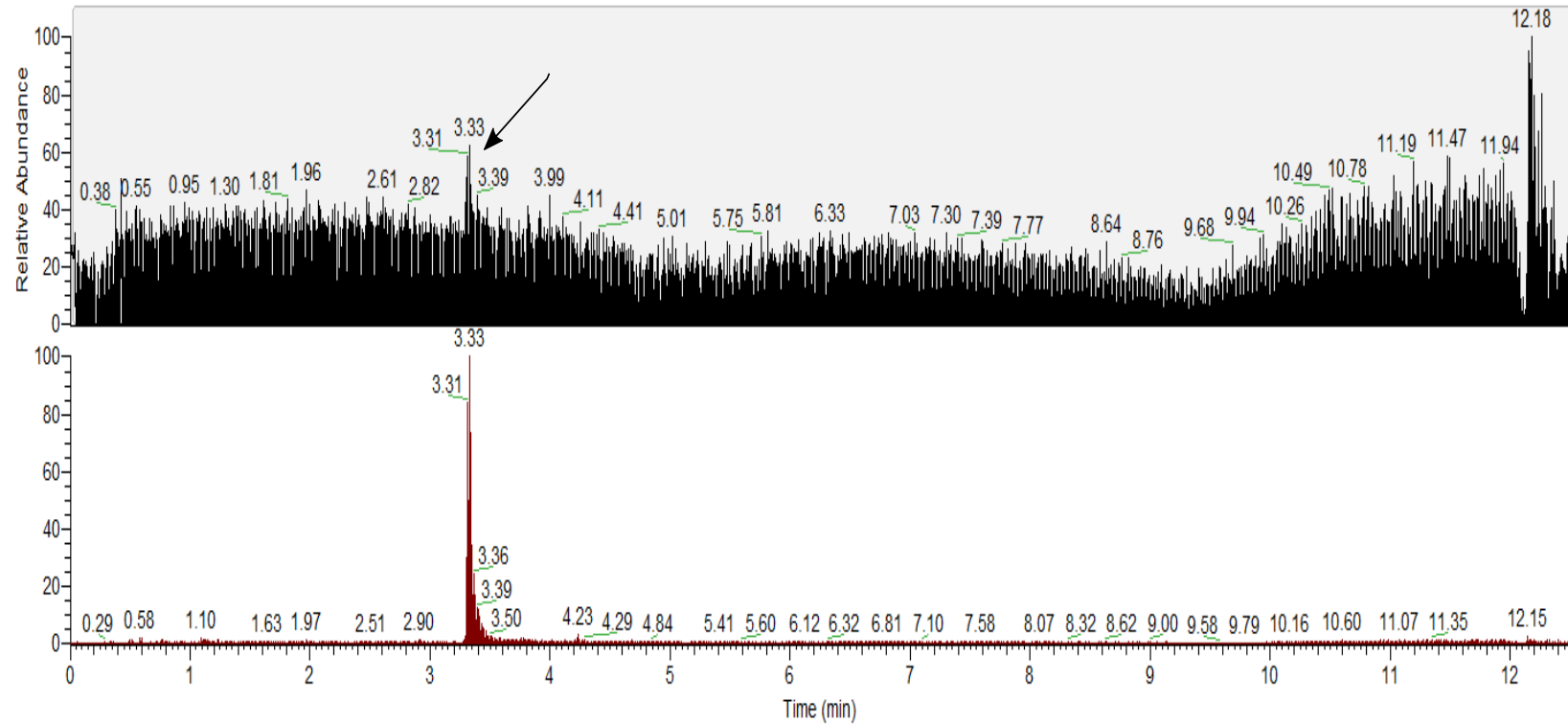**d**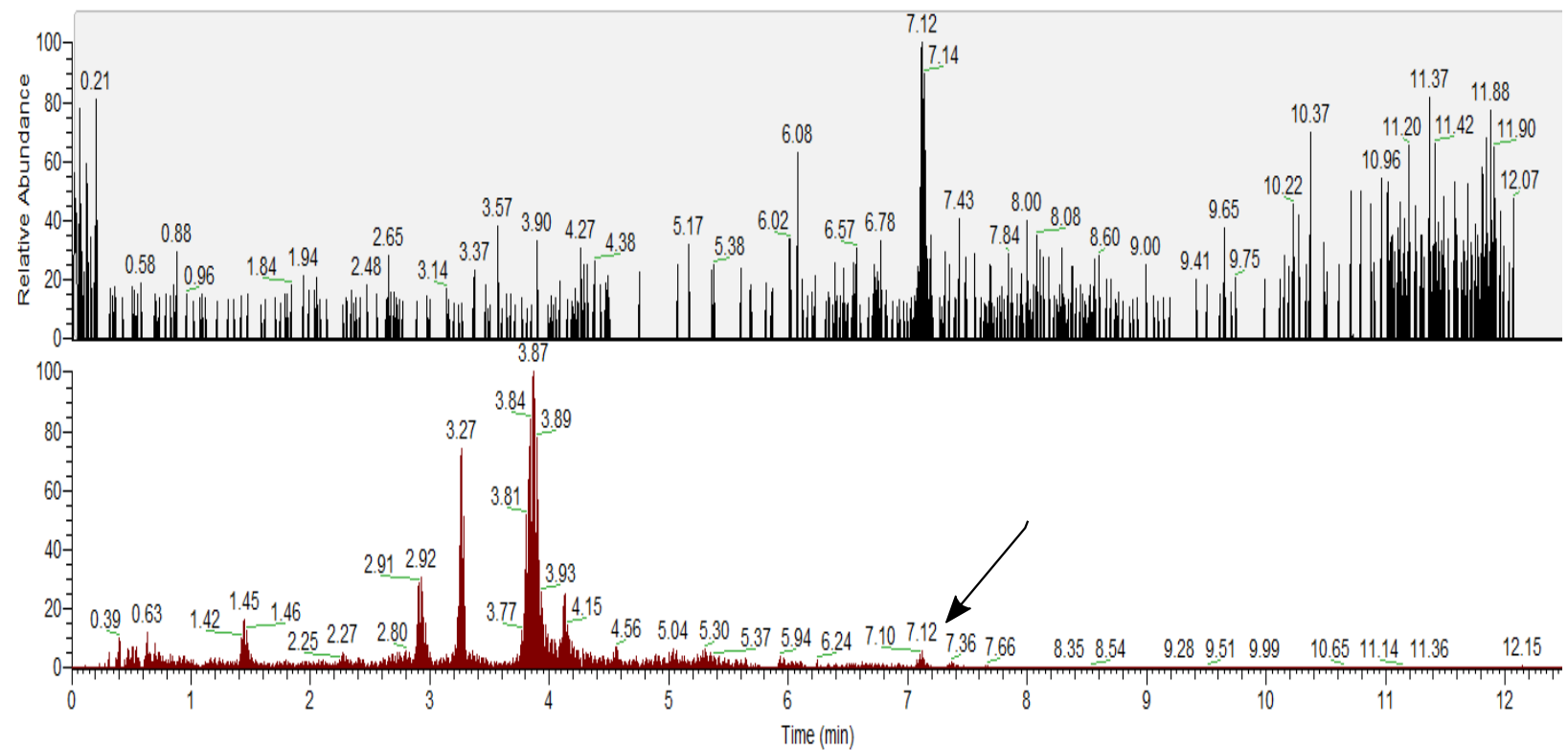

**e**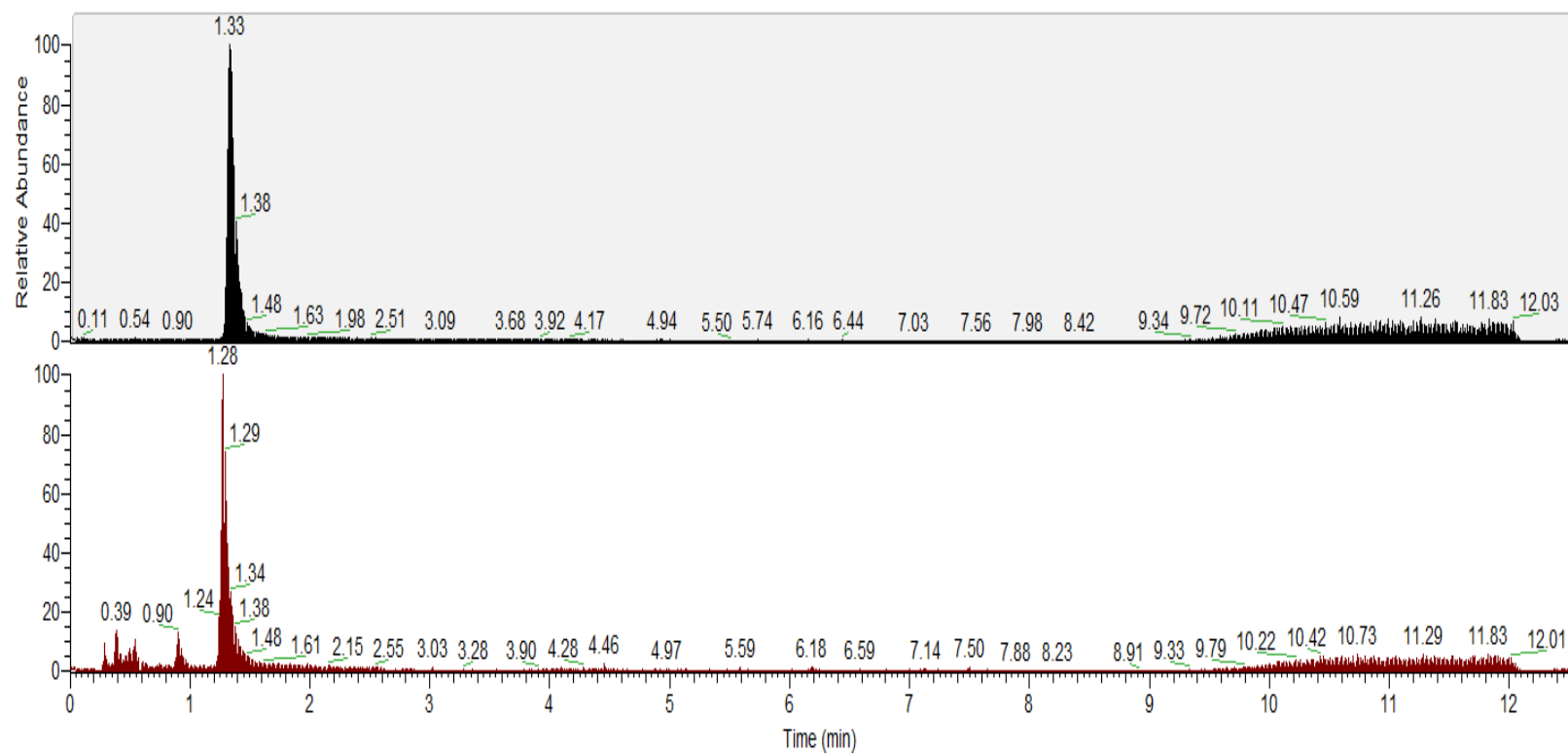**f**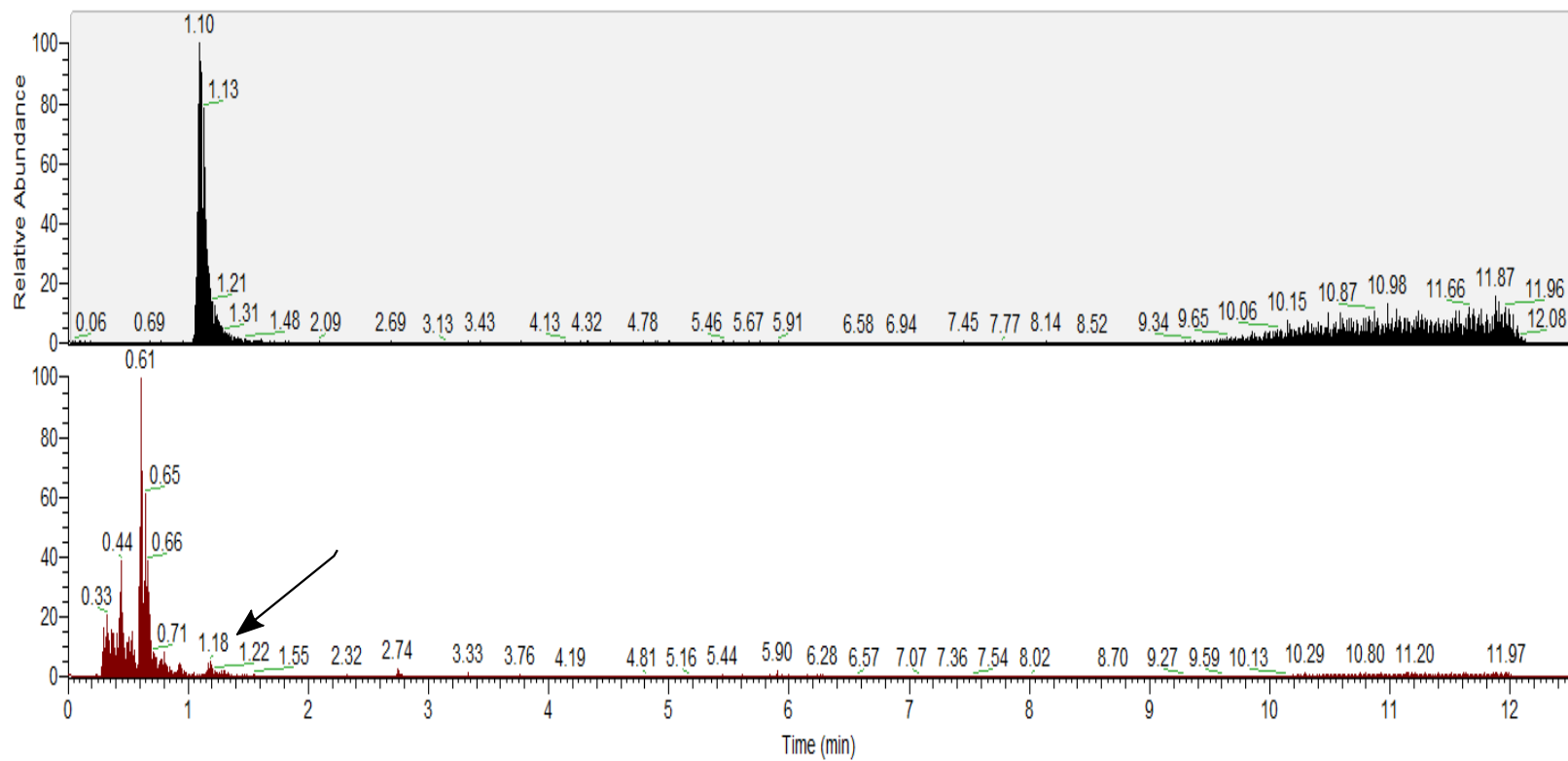

**g**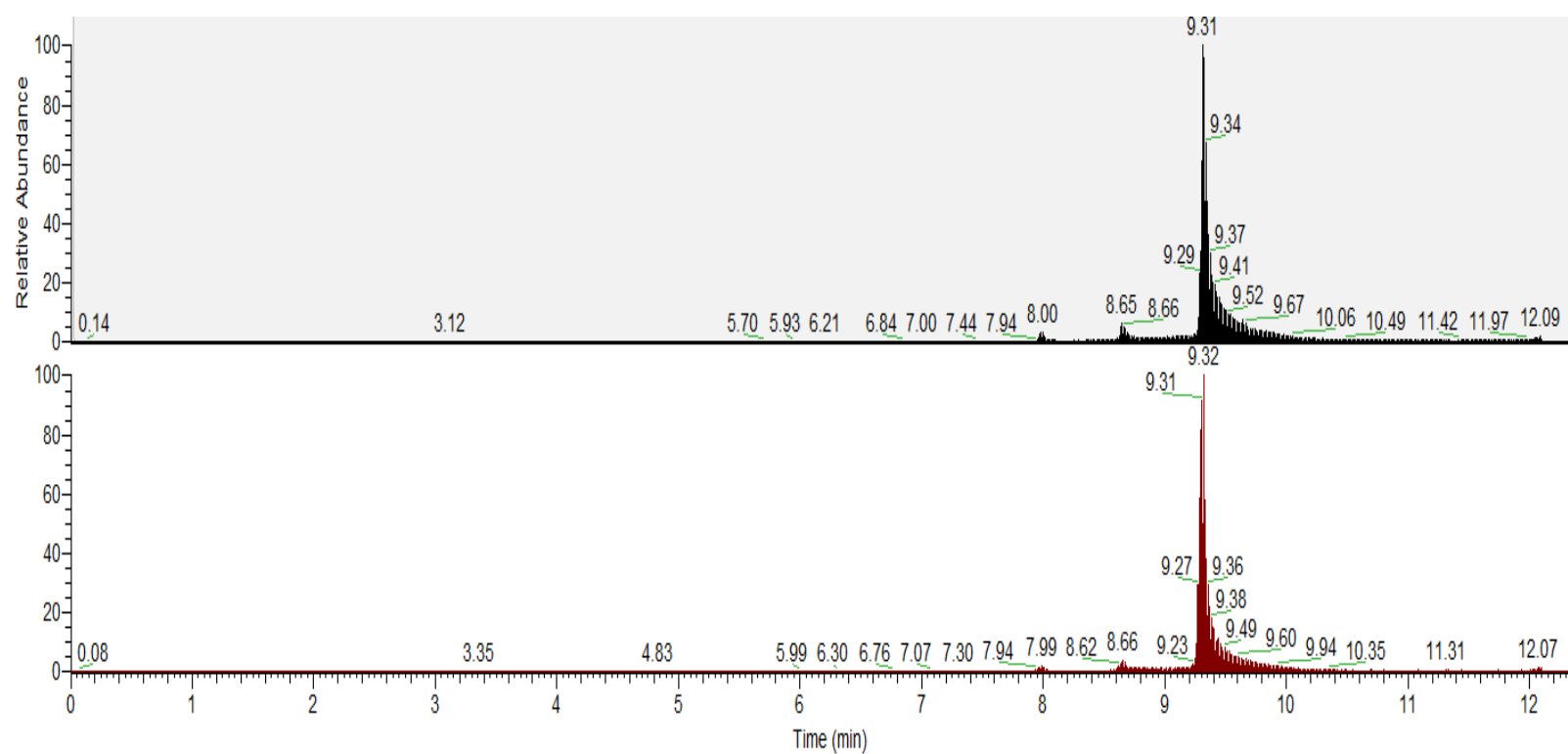**h**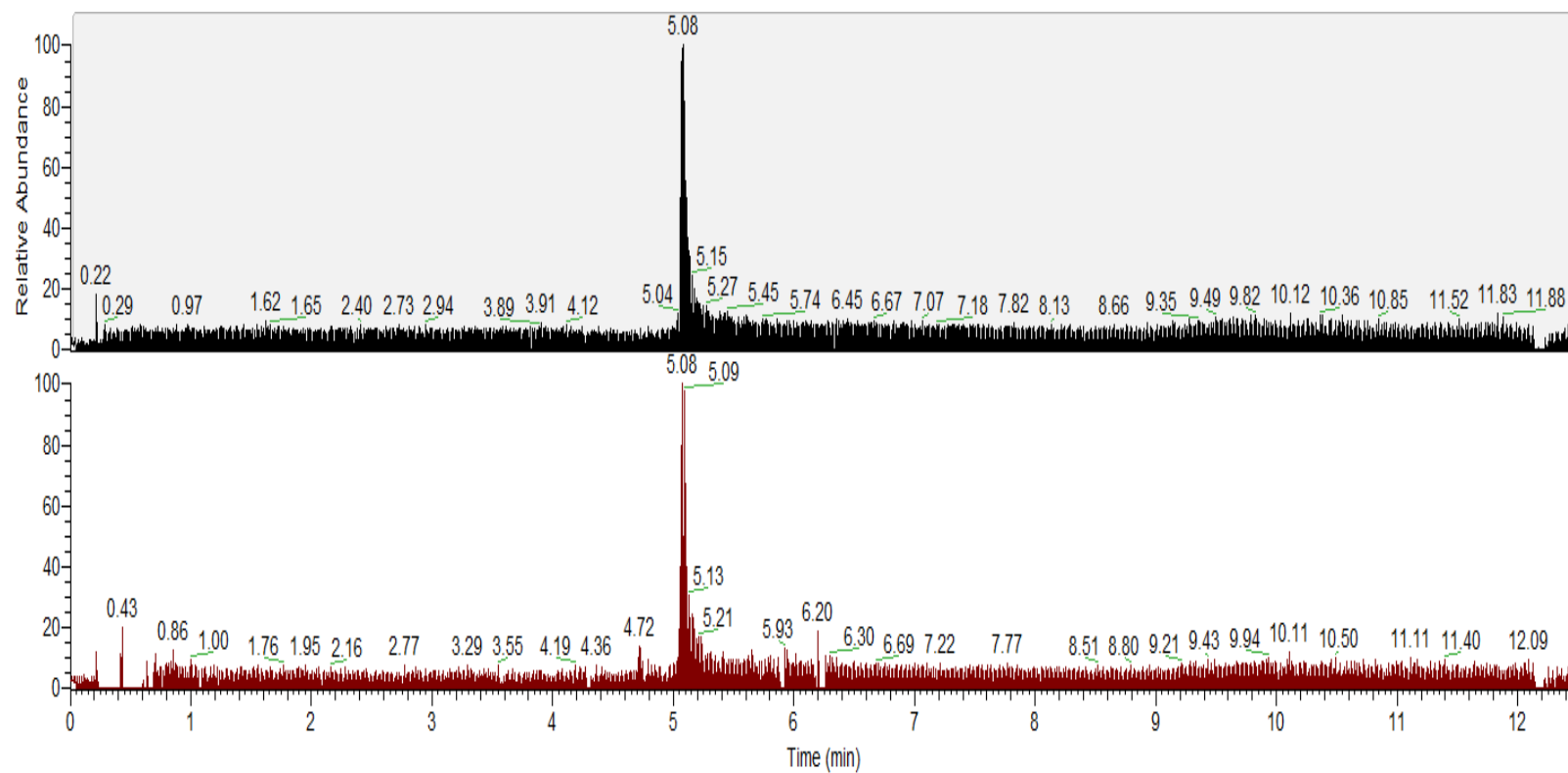

**i**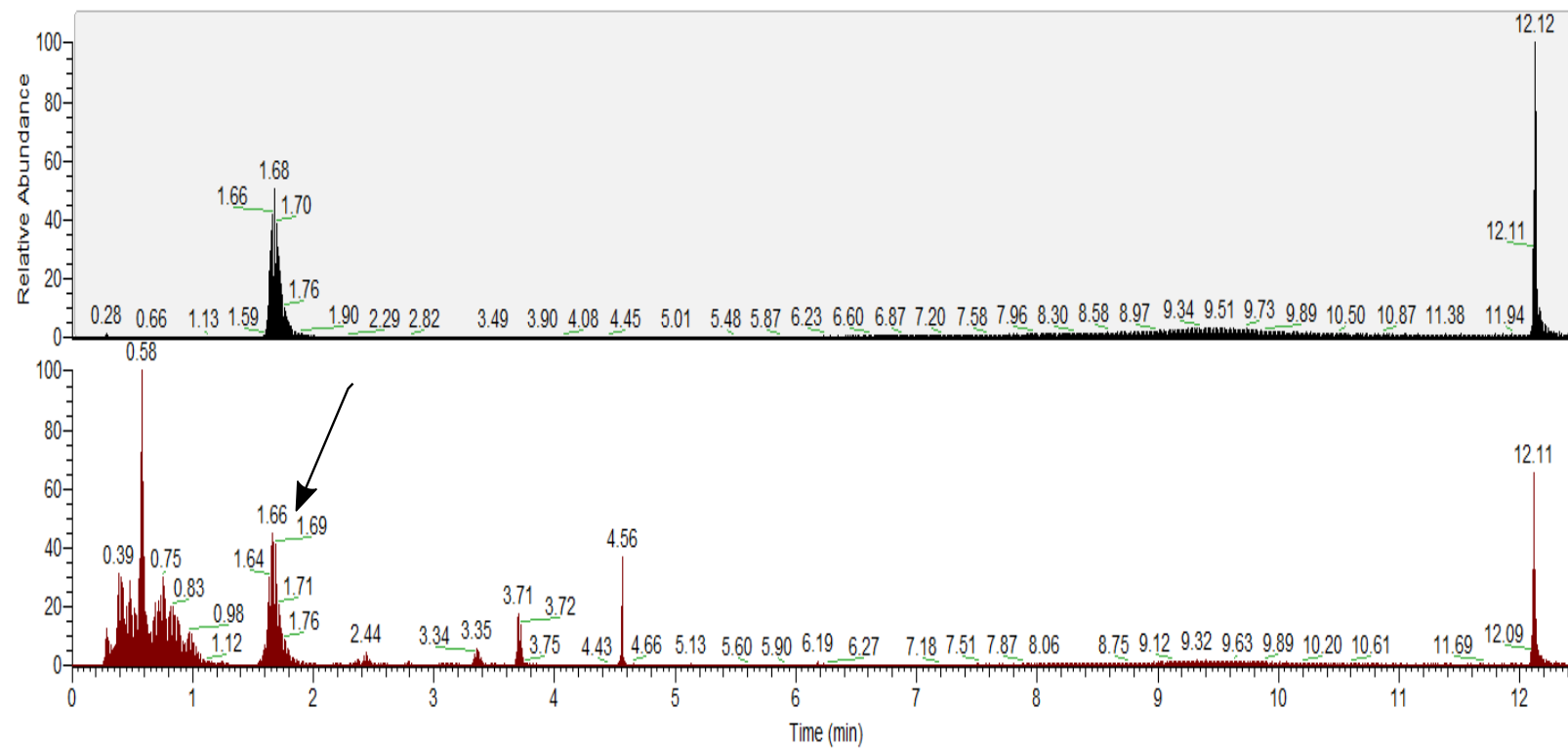**j**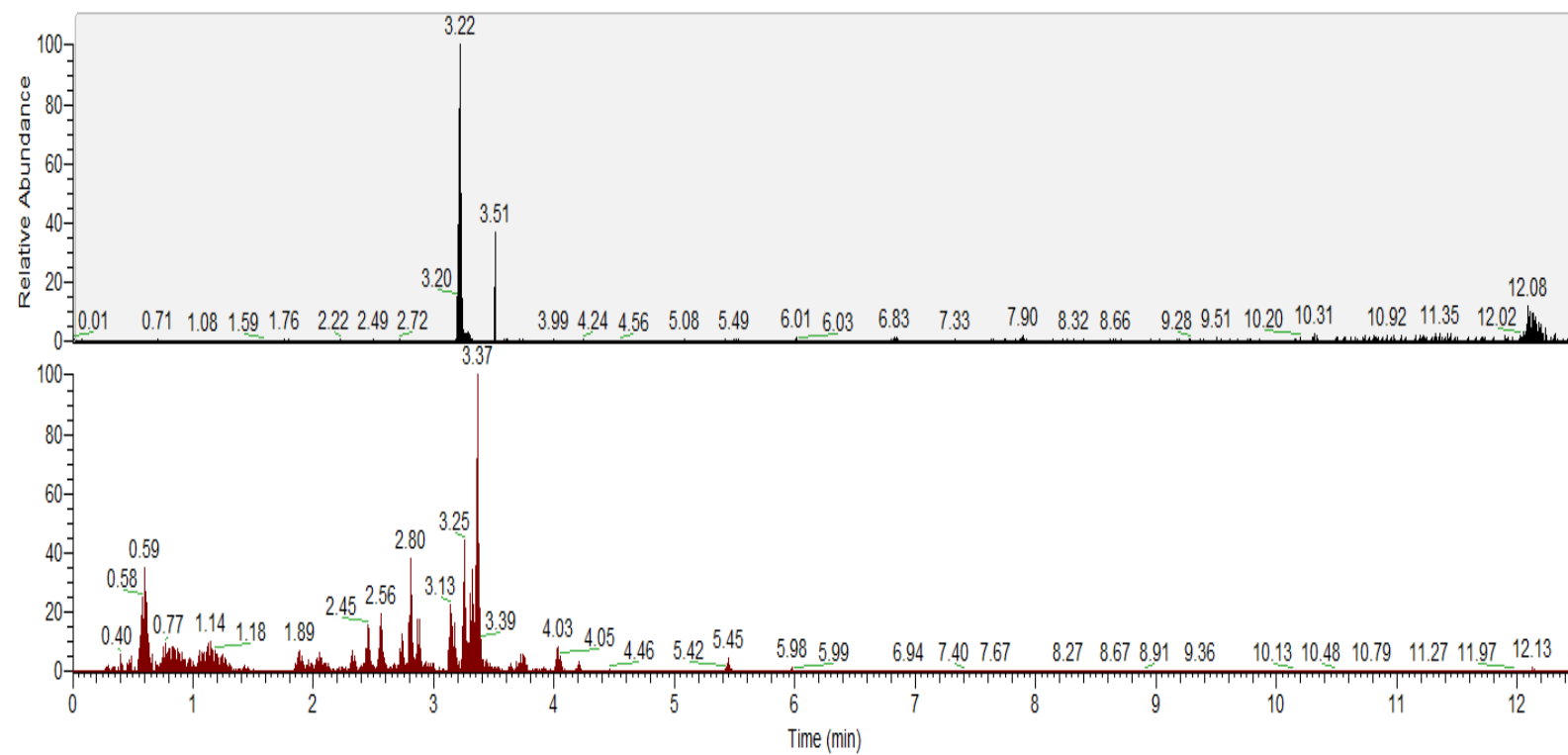

**k**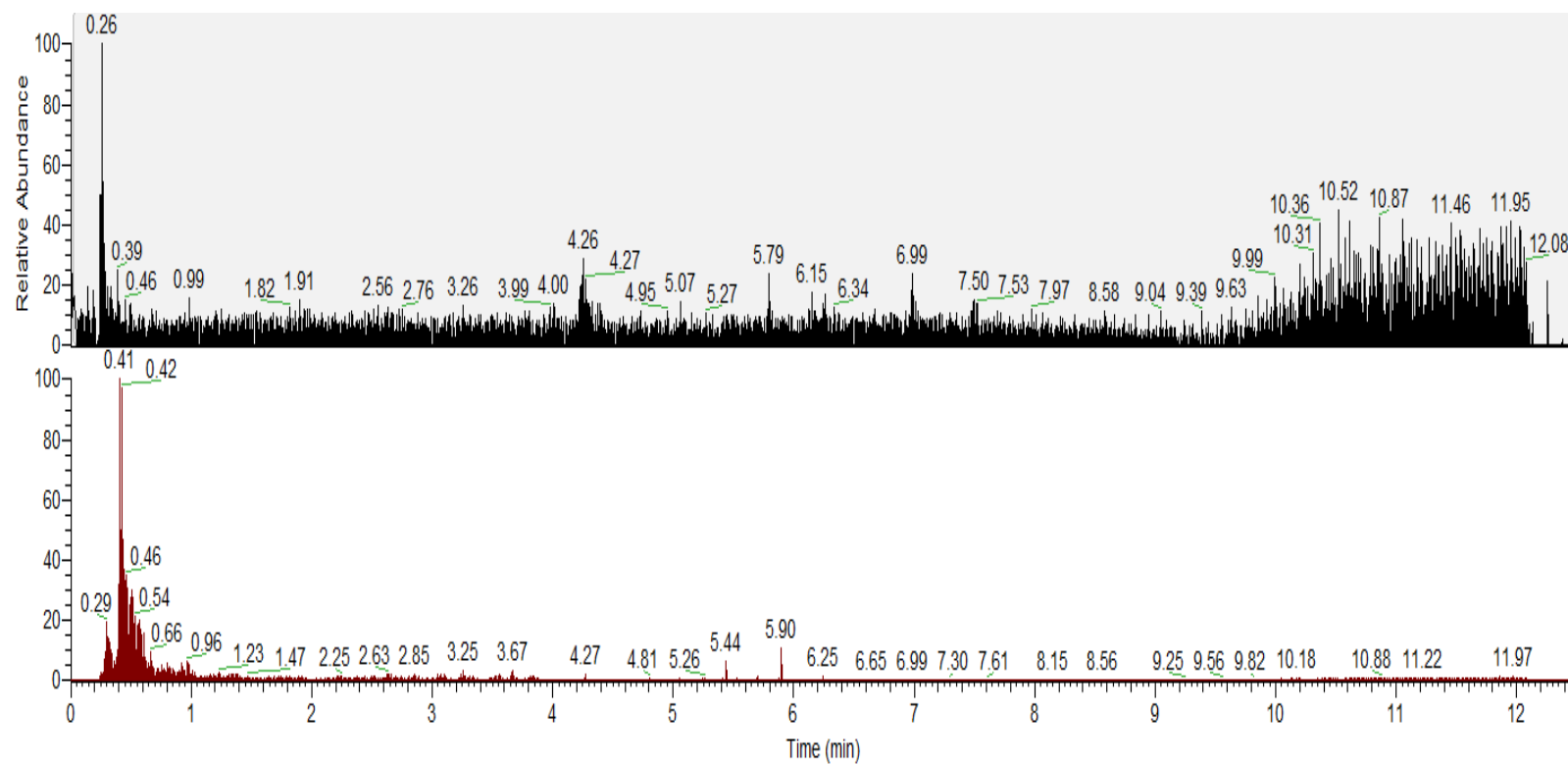**l**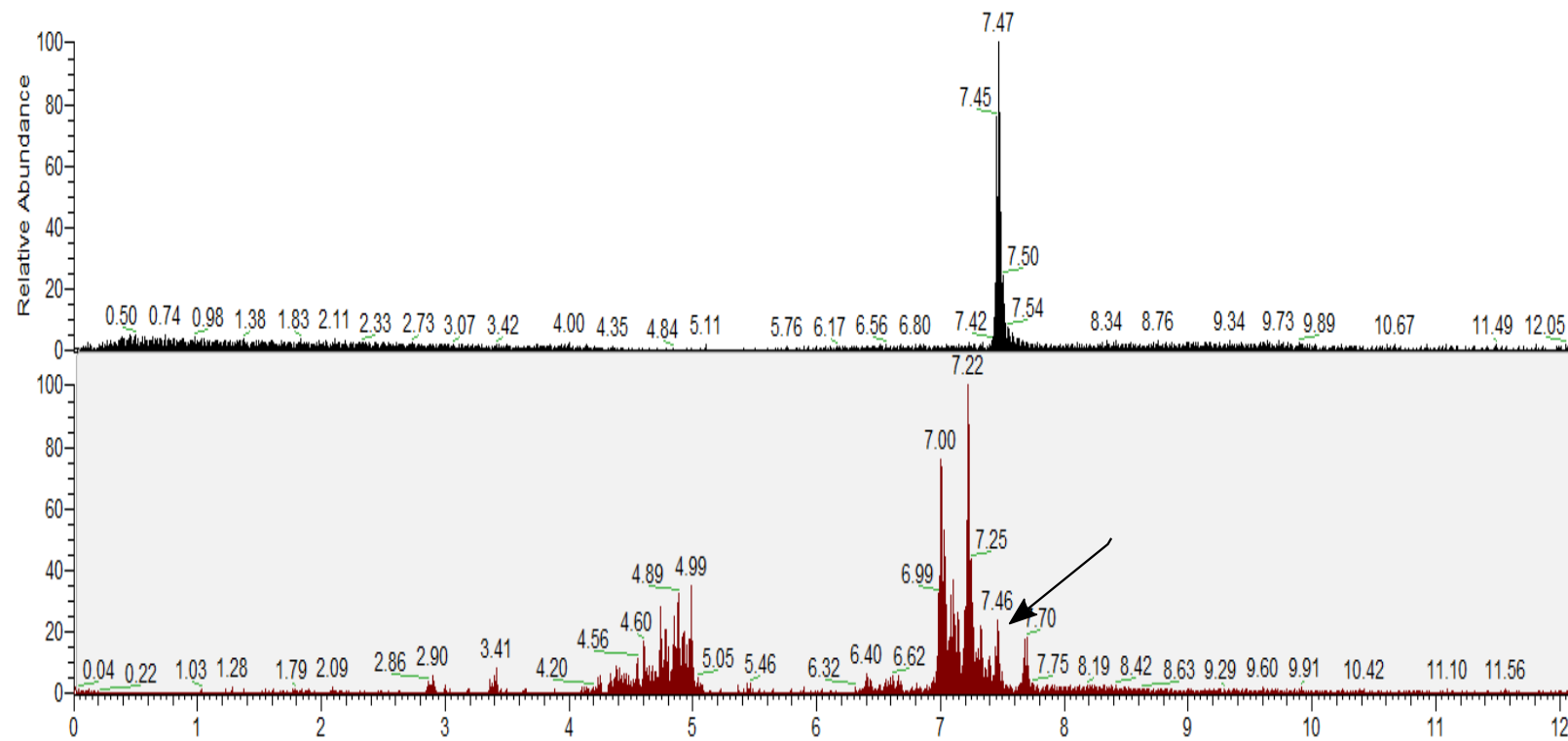

m

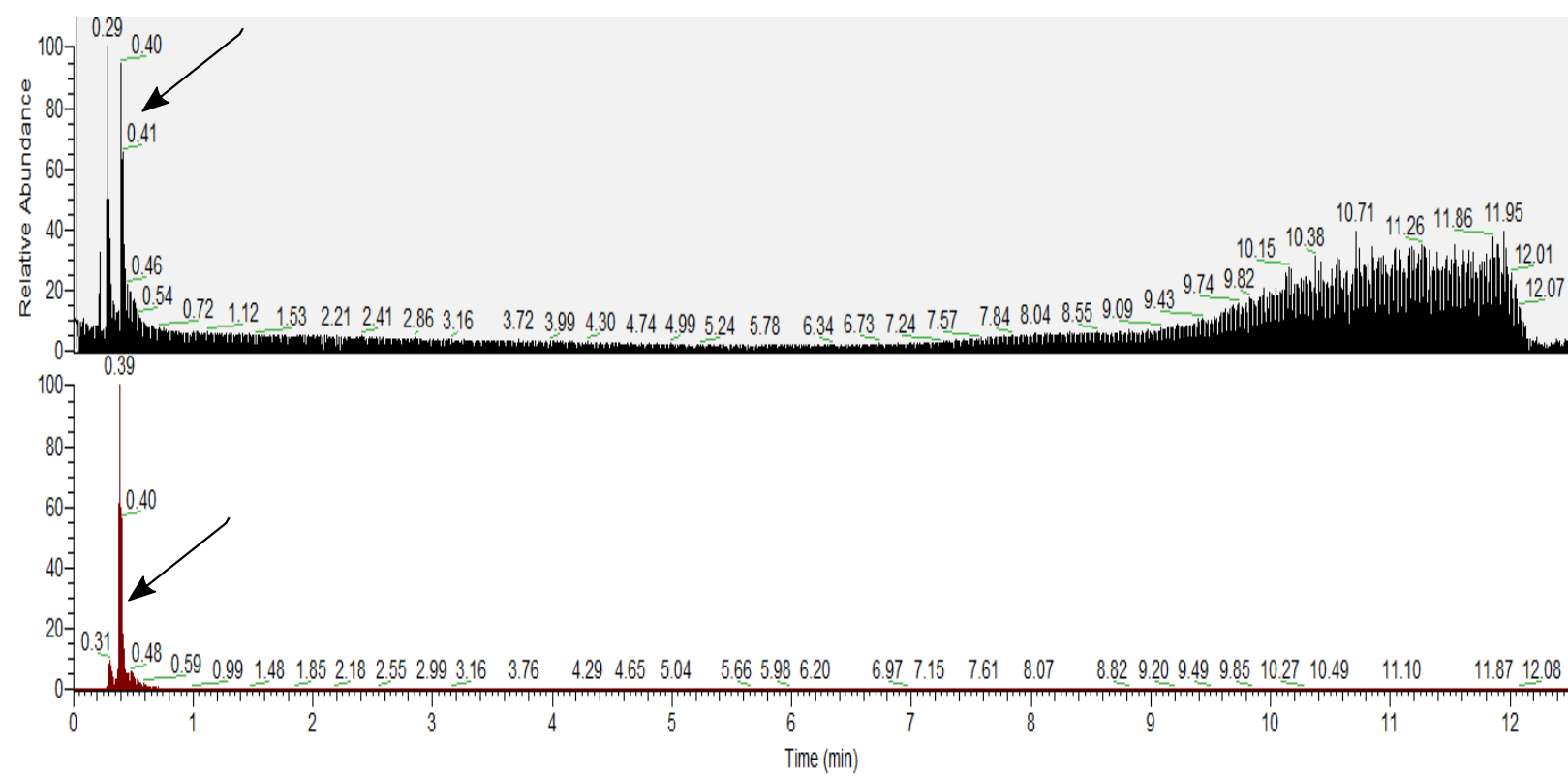

Supplement: FIG S4 [file msystems.00710-22-s0004.pdf]
