# Supplementary material for: Untargeted Fecal Metabolomic Analyses across an Industrialization Gradient Reveal Shared Metabolites and Impact of Industrialization on Fecal Microbiome-Metabolome Interactions
Source: mSystems. 2022 Nov 23;7(6):e00710-22. doi: 10.1128/msystems.00710-22 (PMC9765122; doi:10.1128/msystems.00710-22)
Supplement: TABLE S2 [file msystems.00710-22-s0006.pdf]

**Compound Name**	***m/z***	**RT (min)**	**Cosine Score**	**Detected in 6-sample filtering**	**Detected in half-sample filtering**	**Detected in all-sample filtering**	**Predicted ClassyFire Class**	**Description derived from PubChem and HMDB**	**Present in Human Fecal Metabolome Database**	**ReDU Chemical Explorer Associations**
Hypoxanthine	137.046	0.39	0.98	Yes	Yes	No	-	Purine derivative associated with inosine and uric acid in humans	Yes	Found in bacterial cultures (*Clostridium orbiscindens* CC43_001K)
Nicotinamide N-oxide	139.05	0.31	1	Yes	No	No	-	Precursor to nicotinamide-adenine dinucleotide (NAD+) in animals	No (found in blood)	Associated with *Staphylococcus lugdunensis*. Found in human skin, saliva, and fecal samples
3-methyl-2-oxindole (3-Methyloxindole)	148.076	3.22	0.93	Yes	No	No	Indoles and derivatives (55.56%)	Endogenous product of 3-methylindole	No	Found in bacterial cultures (*Gardnerella vaginalis* and *Collinsella* sp. 4_8_47FAA)
Hyocholic acid	158.154	4.78	0.83	Yes	Yes	No	Steroids and steroid derivatives (100%)	Mammalian bile acid	Yes	Largely found in bacterial culture and humans
Gly-Val (Glycylvaline)	175.107	0.36	0.91	Yes	Yes	Yes	-	Glycine and valine dipeptide	Yes	Found in bacterial cultures, associated with *Staphylococcus aureus*. Also associated with inflammatory bowel disease.

**3-Hydroxy-4-methoxycinnamic acid (Isoferulic acid)**	**177.055**	**4.13**	**0.97**	**Yes**	**Yes**	**No**	**Cinnamic acids and derivatives (9.09%)**	**Endogenous human metabolite; Also potential biomarker for coffee, wheat, sunflowers, etc.**	**Yes**	**Predominantly found in plant, food (such as fruits), and beverage samples. Also found in human caecum and fecal samples.**
**2-Butanone, 4-(2,6,6-trimethyl-2-cyclohexen-1-yl)**	**177.164**	**3.15**	**0.83**	**Yes**	**No**	**No**	**-**	**Also known as 3α,7α,12α-trihydroxycholestanoic acid (THCA); Intermediate bile acid associated with metabolic disorders**	**No**	**Predominantly found in plant samples (*Cucumis melo*), bacterial culture (*Bacteroides dorei*; *Propionibacterium acnes*), and fungal cultures.**
Paraxanthine	181.072	0.81	0.94	Yes	Yes	No	Imidazopyrimidines (21.74%)	Primary metabolite of caffeine; Found in animals and some bacteria	Yes	Not in Chemical Explorer

trans-Ferulic acid	195.065	3.01	0.91	Yes	Yes	No	Cinnamic acids and derivatives (9.09%)	Abundant in plant cell walls	Yes	Predominantly found in plant, food, and beverage samples. Also found in human fecal and caecum samples.
Loliolide	197.117	3.11	0.94	Yes	Yes	No	Benzofurans (13.79%)	Plant metabolite	No	Found in environmental and plant samples (*Cucumis melo*)
3-Hydroxydodecanoic acid	199.169	5.35	0.99	Yes	Yes	No	Lactones (20%)	Medium-chain fatty acid	Yes	Found in environmental samples, plant samples, and some human feces
N-Acetyl-D-mannosamine	204.087	3.27	0.91	Yes	No	No	-	Bacterial metabolite; Precursor to *N*-acetylmannosamine	Yes	Found in a fungal culture (species not specified)

N-acetyl-L-Phenylalanine	208.097	2.84	0.95	Yes	Yes	No	Carboxylic acids and derivatives (10.39%)	Metabolite of phenylalanine; Frequently found in urine of humans with phenylketonuria	Yes	Found in bacterial (*Fusobacterium ulcerans* 12-1B, *Sutterella wadsworthensis* HGA0223, etc.) and some fungal cultures
Thr-Pro (Threonylproline)	217.122	0.46	0.96	Yes	Yes	No	-	Threonine and proline dipeptide	No	Found in bacterial culture, fungal culture, and animal samples (e.g., mice, rats, humans, etc.). Found in human fecal samples. Associated with inflammatory bowel disease samples. Also associated with *Staphylococcus aureus*.
Val-Val (Valylvaline)	217.155	0.45	0.97	Yes	Yes	Yes	Carboxylic acids and derivatives (12.86%)	Valine and valine dipeptide	Yes	Found in fungal cultures and human fecal samples.

Abrine	219.113	0.60	0.88	Yes	Yes	No	-	Associated with *Escherichia coli* metabolism	No	Found in food, beverage, plant, animal, fungal culture, and bacterial culture samples. Also found in human digestive tract, fecal, heart, caecum, skin, and blood samples. Found equally in human rural and urban samples. Associated with many different bacterial species (e.g., *Parabacteroides goldsteinii* CC87F, *Prevotella nigrescens* CC14M, *Bifidobacterium longum*, etc.)
**Pantothenic acid**	**220.118**	**0.56**	**0.81**	**Yes**	**No**	**No**	**-**	**Also called Vitamin B5; essential metabolite for carbohydrate, protein, and fat synthesis**	**Yes**	**Found in animal (human, mouse, rat, etc.) and environmental samples.**
PyroGlu-Pro (Pyroglutamylproline)	227.103	0.44	0.86	Yes	Yes	Yes	-	No information provided	No	Predominantly found in bacterial cultures (*Prevotella nigrescens, Bacteroides stercoris* CC31F, etc.), plant, and human fecal samples

Myristoleic acid	227.201	5.94	0.94	Yes	Yes	Yes	-	Long-chain fatty acid found in all eukaryotes; Potential biomarker for some dairy products and other food (anchovies, dates, sunflowers, chocolate, etc.)	Yes	Predominantly found in environmental samples, such as soil.
Ile-Pro (Isoleucylproline)	229.155	0.7	0.98	Yes	No	No	-	Isoleucine and Proline dipeptide	No (found in blood and sweat)	Predominantly found in bacterial (e.g., *Prevotella denticola; Bacteroides stercoris; Parabacteroides johnsonii*, etc.) and fungal cultures
Val-Ile (Valylisoleucine)	231.171	2.82	0.88	Yes	Yes	Yes	-	Valine and Isoleucine dipeptide	Yes	Found in fungal cultures
**cis-9-Hexadecenoic acid (Palmitoleic acid)**	**237.221**	**6.49**	**0.97**	**Yes**	**Yes**	**Yes**	**-**	**Unsaturated fatty acid**	**Yes**	**Predominantly found in soil samples**
Gly-Tyr (Glycyltyrosine)	239.102	0.44	0.95	Yes	Yes	No	-	Glycine and tyrosine dipeptide	Yes	Found in bacterial cultures (e.g., *Parvimonas micra*, *Fusobacterium nucleatum*, etc.)

Biotin	245.098	2.39	0.87	Yes	No	No	Biotin and derivatives (16.67%)	Also called vitamin H; essential human metabolite	Yes	Found in environmental, animal, and bacterial culture (e.g., *Escherichia coli*, *Staphylococcus aureus*, etc.) samples. Also found in human nasal cavity, skin, and saliva samples.
Leu-Leu (Leucylleucine)	245.186	2.40	0.98	Yes	Yes	Yes	Carboxylic acids and derivatives (12.86%)	Leucine and leucine dipeptide	Yes	Predominantly found in bacterial cultures (*Bacteroides stercoris* CC31F, *Clostridium cadaveris* CC44_001G, etc.) and fungal cultures (not specified). Also found in human fecal samples.
Lenticin	247.145	1.22	0.98	Yes	No	No	-	Found in lentil extracts; Possible lentil biomarker	Yes	Found in food, animal, and bacterial culture samples (e.g., *Staphylococcus aureus*). Also found in human urine, milk, blood, and saliva samples.

Val-Met (Valylmethionine)	249.126	0.56	0.95	Yes	Yes	No	-	Valine and Methionine dipeptide	Yes	Not in Chemical Explorer
Ser-Phe (Serylphenylalanine)	253.118	0.79	0.93	Yes	Yes	Yes	-	Serine and phenylalanine dipeptide	Yes	Found in fungal cultures, bacterial cultures (*Bacteroides stercoris* CC31F, *Clostridium cadaveris* CC88A, etc.) and human fecal samples
**Palmitelaidic acid**	**255.232**	**5.96**	**0.92**	**Yes**	**No**	**No**	**-**	**Trans fatty acid**	**Yes**	**Found in human colon, upper digestive tract, liver, and fecal samples. Also found in soil samples.**
L-Saccharopine	259.129	0.31	0.78	Yes	Yes	No	-	Involved in lysine degradation	Yes	Found in bacterial culture (*Staphylococcus aureus* JE3) and fungal cultures (not specified)

Phe-Pro (Phenylalanylproline)	263.139	2.57	0.99	Yes	Yes	Yes	-	Phenylalanine and Proline dipeptide	Yes	Predominantly found in bacterial cultures (*Bacteroides dore*i CL03T12C01, *Prevotella histicola*, *Parabacteroides goldsteinii* CC87F, etc.) and some fungal cultures
**Conjugated linoleic Acid (10E,12Z)**	**263.237**	**7.52**	**0.85**	**Yes**	**Yes**	**No**	**Prenol lipids (5.36%)**	**Variation of conjugated linoleic acid; Found in meat dairy products of ruminants; Dietary supplement**	**No**	**Found in human digestive tract, liver, colon, and feces. Also found in environmental and fungal samples.**
Conjugated linoleic acid (9E,11E)	263.237	6.68	0.92	Yes	Yes	Yes	Prenol lipids (5.36%)	Variation of conjugated linoleic acid; Found in meat dairy products of ruminants; Dietary supplement	No (found in blood)	Found in environmental samples and fungal cultures. Also found in human upper digestive tract, liver, and feces.
Thr-Phe (Threonylphenylalanine)	267.134	0.48	0.94	Yes	Yes	Yes	-	Threonine and Phenylalanine dipeptide	Yes	Found in fungal cultures
Pro-Arg (Prolylarginine)	272.171	0.32	0.72	Yes	No	No	-	Proline and arginine dipeptide	No	Found in a fungal culture (species not specified)

9-OxoOTrE	275.201	4.42	0.8	Yes	Yes	No	-	Long-chain fatty acid	No	Found in fungal cultures and plant samples (*Zea mays* L. and *Cucumis melo*)
Phe-Leu (Phenylalanylleucine)	279.171	2.59	0.99	Yes	Yes	Yes	Carboxylic acids and derivatives (12.86%)	Phenylalanine and leucine dipeptide	No	Found in human upper digestive tract, colon, and fecal samples. Also found in bacterial cultures (*Parabacteroides goldsteinii* CC87F, *Prevotella nigrescens* CC14M, etc.) and fungal cultures (not specified)
Leu-Phe (Leucylphenylalanine)	279.171	3.47	0.98	Yes	Yes	Yes	-	Leucine and phenylalanine dipeptide	Yes	Found in animal, fungal culture, and bacterial culture (e.g., *Bacteroides dorei*, *Staphylococcus aureus*, *Propionibacterium acidifaciens*, etc.) samples. Also found in human intestinal, vaginal, and fecal samples
N-Tetracosenoyl-4-sphingenine	282.279	6.09	0.95	Yes	Yes	No	Fatty Acyls (11.54%)	Ceramide associated with cell physiology and some human pathologies	Yes	Not in Chemical Explorer

Octadecanamide	284.295	8.55	0.77	Yes	No	No	-	Metabolite derived from stearic acid, found in plant and animal fats	Yes	Found in environmental and plant samples
Xanthosine	285.083	0.40	0.9	Yes	Yes	No	-	Purine nucleoside	Yes	Found in built environment, animal, and bacterial culture samples.
Arg-Ile (Arginylisoleucine)	288.203	0.36	0.96	Yes	Yes	No	-	Arginine and isoleucine dipeptide	Yes	Found in food and animal samples. Also found in human stomach, saliva, spleen, and fecal samples.
cis-11,14-Eicosadienoic acid	291.268	5.54	0.79	Yes	No	No	Prenol lipids (5.36%)	Omega-6 fatty acid found in human milk	Yes	Found in bacterial culture (*Propionibacterium acnes*) and plant samples
N-Acetylmuramic Acid	294.119	0.38	0.84	Yes	Yes	Yes	Carboxylic acids and derivatives (4.08%)	Component of bacterial cell walls	Yes	Found in animal samples. In humans, found in caecum and fecal samples.

**Tyr-Leu (Tyrosylleucine)**	**295.165**	**2.71**	**0.97**	**Yes**	**Yes**	**Yes**	**Carboxylic acids and derivatives (31.25%)**	**Tyrosyl and leucine dipeptide**	**Yes**	**Found in fungal and bacterial cultures (*Prevotella nigrescens* CC14M). Also found in human feces**
Phe-Met (Phenylalanylmethionine)	297.126	2.18	0.93	Yes	Yes	No	-	Phenylalanine and methionine dipeptide	Yes	Found in bacterial cultures (*Bacteroides stercoris* CC31F, *Prevotella bivia*, etc.).
Ile-Gly-Ile (Isoleucylglycylisoleucine)	302.205	3.04	0.94	Yes	Yes	No	-	Isoleucine, glycine, and isoleucine tripeptide	No	Found in animal and bacterial culture samples (*Staphylococcus aureus*, *Bacteroides dorei*, *Bacteroides stercoris*, etc.). Also found in human samples collected from different body parts (duodenum, jejunum, urine, ileum, colon, saliva, stomach, fecal, etc.)

Val-Trp (Valyltryptophan)	304.167	3.02	0.97	Yes	Yes	No	-	Valine and tryptophan dipeptide	Yes	Found in fungal culture, food, and animal samples. Also found in human jejunum, stomach, duodenum, vagina, spleen, ileum, and fecal samples
Fructoselysine	309.164	0.31	0.85	Yes	No	No	Carboxylic acids and derivatives (4.08%)	Potential biomarker for milk and milk products	No (found in blood)	Found in animal samples. Small percentage also found in food samples. Found in human jejunum, kidney, ileum, fecal, and blood samples
N-Palmitoylglycine	314.27	7.34	0.86	Yes	No	No	-	Human metabolite with fatty acid group	No	Found in environmental and animal samples.
Ile-Trp (Iseoleucyltryptophan)	318.167	2.27	0.97	Yes	Yes	Yes	-	Isoleucine and tryptophan dipeptide	Yes	Found in bacterial cultures (*Bacteroides stercoris* CC31F, *Selenomonas noxia*, etc.). Small percentage also in animal and fungal cultures. Also found in human intestinal samples

**Lithocholic acid**	**323.273**	**6.84**	**0.97**	**Yes**	**Yes**	**Yes**	**Prenol lipids (11.11%)**	**Secondary bile acid**	**Yes**	**Found in human feces.**
Leucine enkephalin	336.192	3.22	0.77	Yes	Yes	Yes	-	Enkephalin peptide; Produced in brain	No (found in blood)	Found in bacterial cultures (*Parabacteroides goldsteinii* CC87F, *Parabacteroides merdae*, etc.)
13-Docosenamide, (Z)- (Erucamide)	338.342	9.19	0.83	Yes	Yes	Yes	-	Fatty amide	No	Found in human colon, upper digestive tract, liver, and fecal samples. Also found in bacterial cultures (*Peptostreptococcus sp*. CC14N, *Bifidobacterium longum subsp. longum* 44B, etc.) and fungal cultures
Phe-Trp (Phenylalanyltryptophan)	352.166	3.28	0.96	Yes	Yes	No	-	Phenylalanine and tryptophan dipeptide	Yes	Found in fungal cultures (species not specified)
Ile-Val-Lys (Isoleucylvalyllysine)	359.266	0.60	0.91	Yes	Yes	Yes	-	Isoleucine, valine, and lysine tripeptide	No	Found in animal and bacterial culture samples. Also found in human jejunum, duodenum, ileum, caecum, colon, vaginal, and fecal samples

**Cholesterol**	**369.352**	**10.50**	**0.97**	**Yes**	**Yes**	**Yes**	**-**	**Animal sterol from body tissues and plasma**	**Yes**	**Found in plant and environmental samples.**
**(R)-4-((3R,5S,8R,9S,10S,13R,14S,17R)-3-hydroxy-10,13-dimethyl-7,12-dioxohexadecahydro-1H-cyclopenta[a]phenanthren-17-yl)pentanoic acid**	**405.264**	**4.81**	**0.92**	**Yes**	**Yes**	**No**	**Steroids and steroid derivatives (46.15%)**	**NA**	**No**	**Predominantly found in bacterial cultures (*Bacteroides caccae, Bacteroides ovatus,Clostridium orbiscindens*, etc.). Also found in mouse and human digestive tract and fecal samples.**
**Glycoursodeoxycholic acid**	**414.301**	**5.01**	**0.9**	**Yes**	**Yes**	**No**	**Steroids and steroid derivatives (36.71%)**	**Secondary bile acid**	**Yes**	**Found in animal samples (rats and humans). Also found in human blood plasma, blood serum, urine, skin, and fecal samples.**
**Cholic acid**	**426.318**	**5.19**	**0.98**	**Yes**	**Yes**	**Yes**	**Steroids and steroid derivatives (8%)**	**Bile acid produced in liver**	**Yes**	**Found in human GI tract and feces. Associated with urban samples. Also found in bacterial cultures**

6R)-2-(hydroxymethyl)-6-((3R,5R,7R,8R,9S,10S,12S,13R,14S,17R)-3,7,12-trihydroxy-10,13-dimethylhexadecahydro-1H-cyclopenta[a]phenanthren-17-yl)heptanoic acid	431.318	5.25	0.86	Yes	Yes	No	Steroids and steroid derivatives (6.12%)	NA	No	Found in human feces (<18 years old)
Oleanolic acid	439.359	7.62	0.92	Yes	No	No	-	Plant metabolite	Yes	Found in fungal culture, food, plant, environment, and beverage samples. In humans, found in caecum and fecal samples.
**Glycocholic acid**	**466.32**	**4.69**	**0.96**	**Yes**	**Yes**	**No**	**Steroids and steroid derivatives (13.04%)**	**Secondary bile acid**	**Yes**	**Found in bacterial culture (e.g., *Enterococcus faecium*, *Prevotella oralis*, etc.) and food samples. In humans, found in urine, jejunum, digestive tract, and fecal samples.**

**Enoxolone**	**471.347**	**6.92**	**0.86**	**Yes**	**No**	**No**	**-**	**Derived from plant metabolite; Commonly used as artificial sweetener**	**No (found in blood)**	**Found in human feces (largely individuals <18 years) and fungal cultures**
**Bilirubin**	**585.272**	**8.90**	**0.95**	**Yes**	**Yes**	**No**	**-**	**Bile pigment produced during heme breakdown**	**Yes**	**Found in upper digestive tract and feces of humans.**
**Urobilin**	**591.318**	**4.07**	**0.96**	**Yes**	**Yes**	**Yes**	**-**	**Responsible for yellow coloring of urine**	**Yes**	**Found in urban human colon and fecal samples.**
**Stercobilin**	**595.349**	**4.05**	**0.94**	**Yes**	**Yes**	**No**	**Tetrapyrroles and derivatives (53.85%)**	**Responsible for brown coloring of feces**	**Not available**	**Found in human fecal samples.**
