# Supplementary material for: Untargeted Fecal Metabolomic Analyses across an Industrialization Gradient Reveal Shared Metabolites and Impact of Industrialization on Fecal Microbiome-Metabolome Interactions
Source: mSystems. 2022 Nov 23;7(6):e00710-22. doi: 10.1128/msystems.00710-22 (PMC9765122; doi:10.1128/msystems.00710-22)
Supplement: FIG S3 [file msystems.00710-22-s0003.pdf]

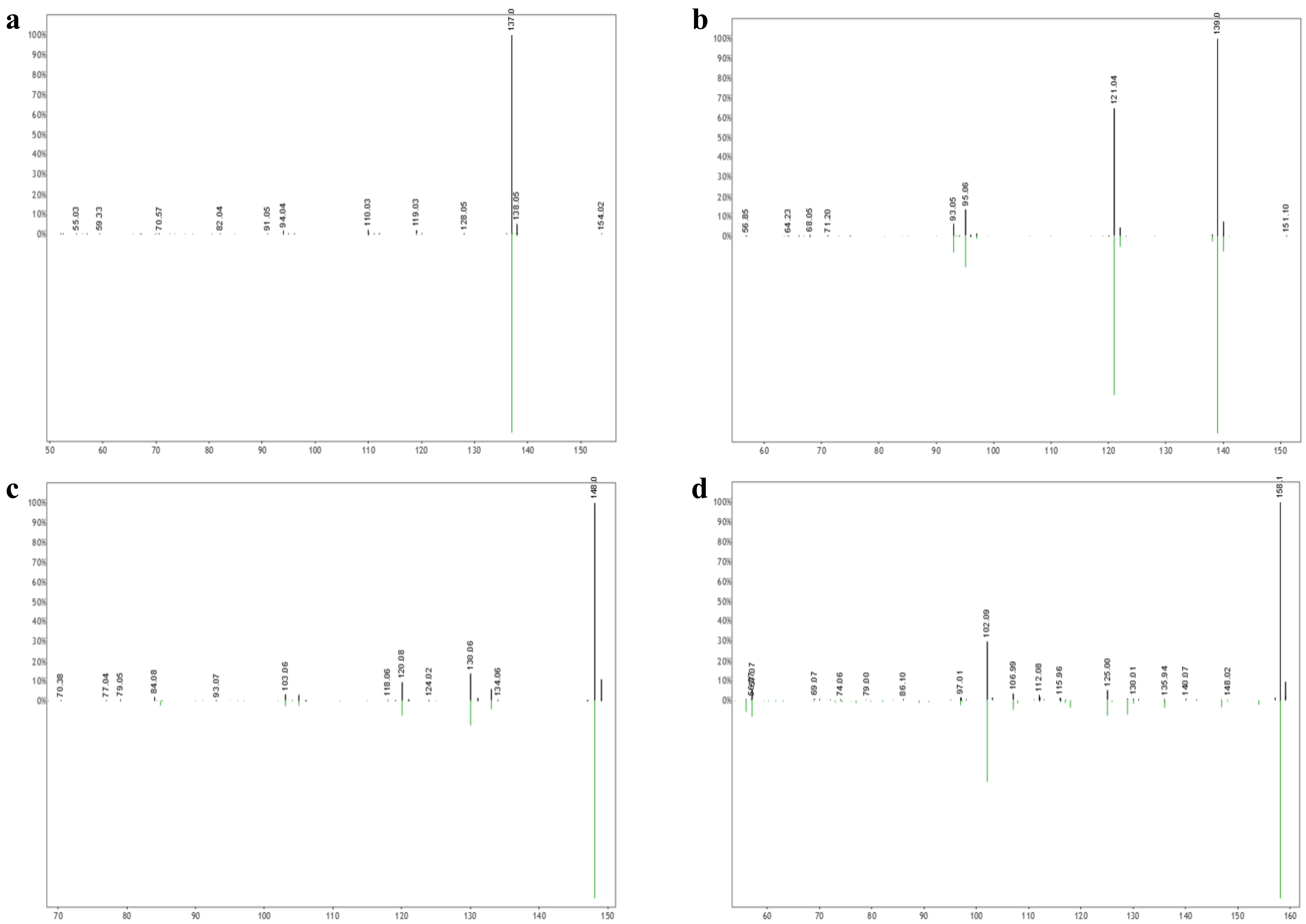

a
137.0
138.05
55.03
59.33
70.57
82.04
91.05
94.04
110.03
119.03
128.05
154.02
b
139.0
121.04
93.05
95.06
56.85
64.23
68.05
71.20
151.10
c
148.0
70.38
77.04
79.05
84.08
93.07
103.06
118.06
120.08
124.02
130.06
134.06
d
158.1
102.09
69.07
74.06
79.00
86.10
97.01
106.99
112.08
115.96
125.00
130.01
135.94
140.07
148.02

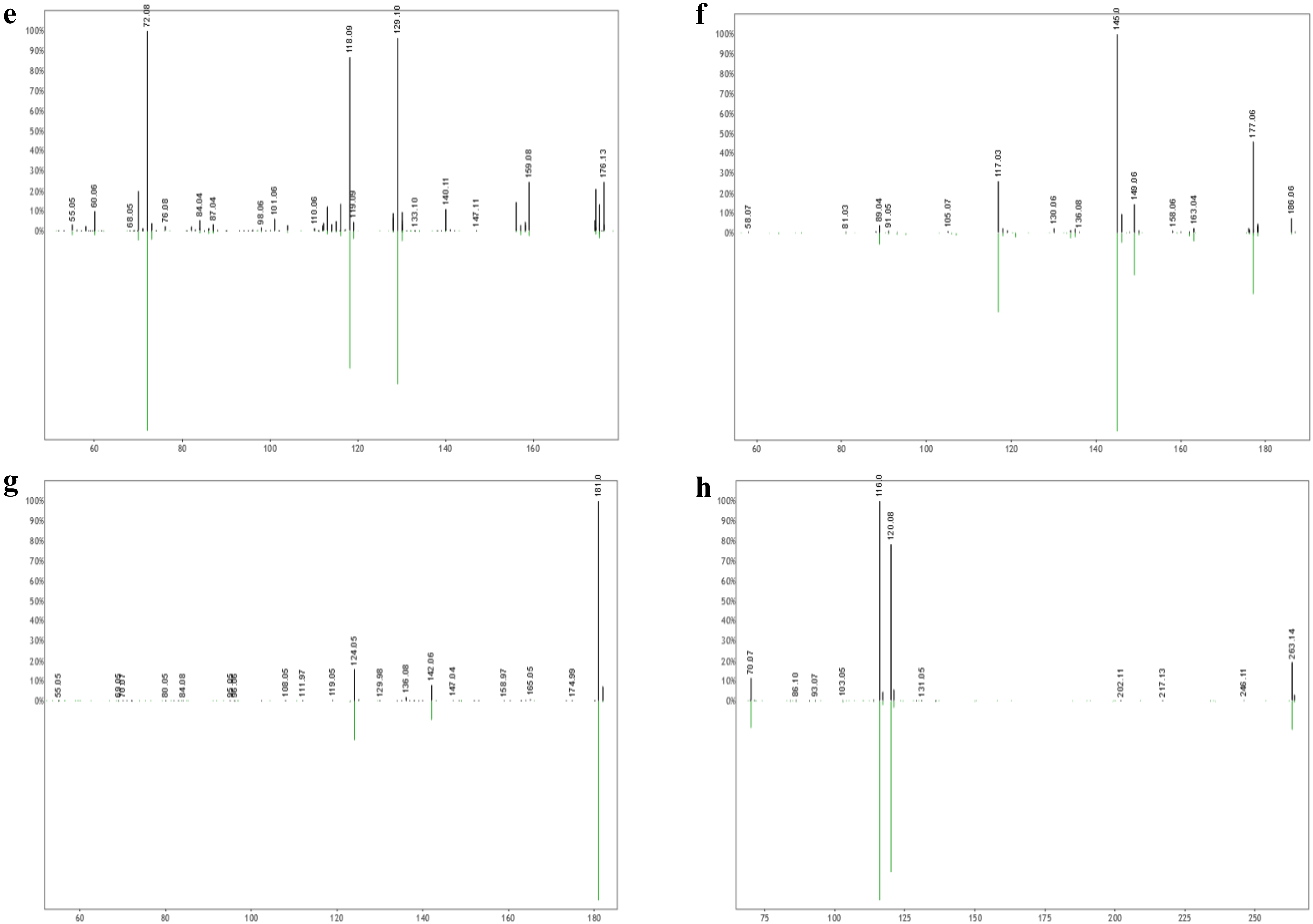
e
72.08
118.09
129.10
55.05
60.06
68.05
76.08
84.04
87.04
98.06
101.06
110.06
119.09
133.10
140.11
147.11
159.08
176.13
f
145.0
177.06
117.03
149.06
58.07
81.03
89.04
91.05
105.07
130.06
136.08
158.06
163.04
186.06
g
181.0
124.05
142.06
55.05
80.05
84.08
108.05
111.97
119.05
129.98
136.08
147.04
158.97
165.05
174.99
h
116.0
120.08
263.14
70.07
86.10
93.07
103.05
131.05
202.11
217.13
246.11
100%
90%
80%
70%
60%
50%
40%
30%
20%
10%
0%

i j

k l

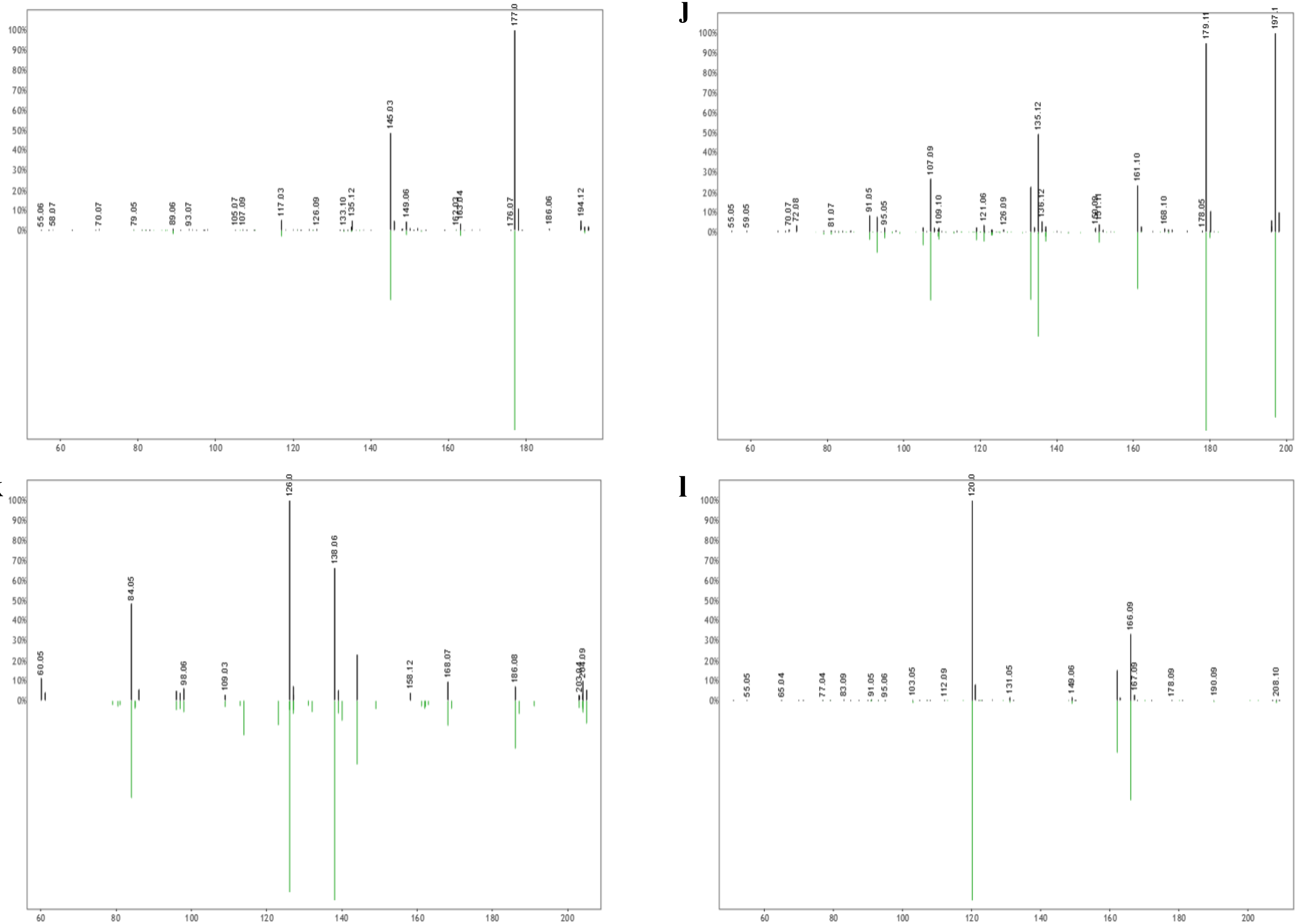

177.0
145.03
55.06
58.07
70.07
79.05
89.06
93.07
105.07
107.09
117.03
126.09
133.10
135.12
149.06
176.07
186.06
194.12
179.11
197.1
135.12
107.09
161.10
55.05
59.05
70.07
72.08
81.07
91.05
95.05
109.10
121.06
126.09
136.12
168.10
178.05
126.0
138.06
84.05
60.05
98.06
109.03
158.12
168.07
186.08
204.09
120.0
166.09
55.05
65.04
77.04
83.09
91.05
95.06
103.05
112.09
131.05
149.06
167.09
178.09
190.09
208.10

m

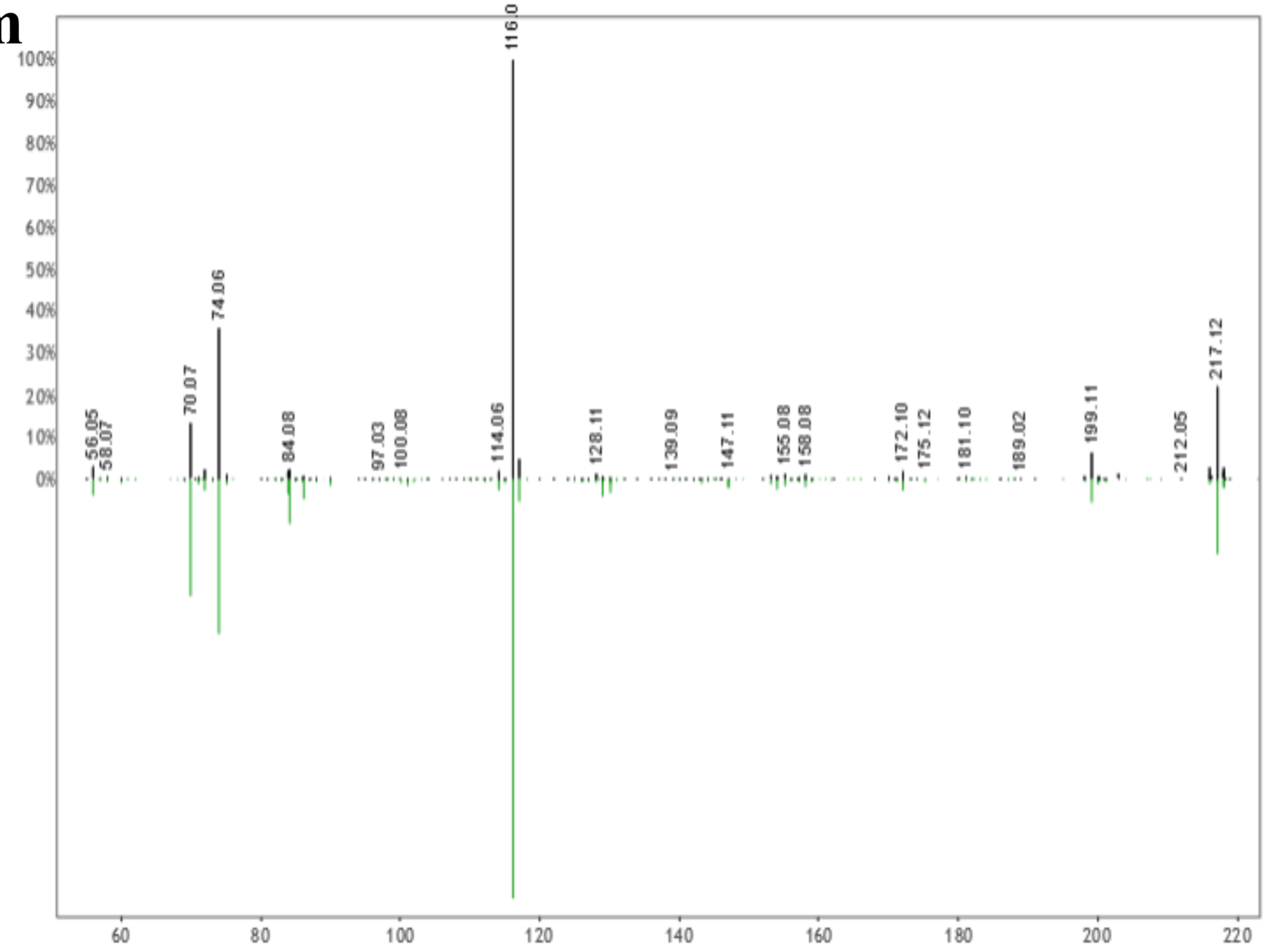
56.05
58.07
70.07
74.06
84.08
97.03
100.08
114.06
116.0
128.11
139.09
147.11
155.08
158.08
172.10
175.12
181.10
189.02
199.11
212.05
217.12

n

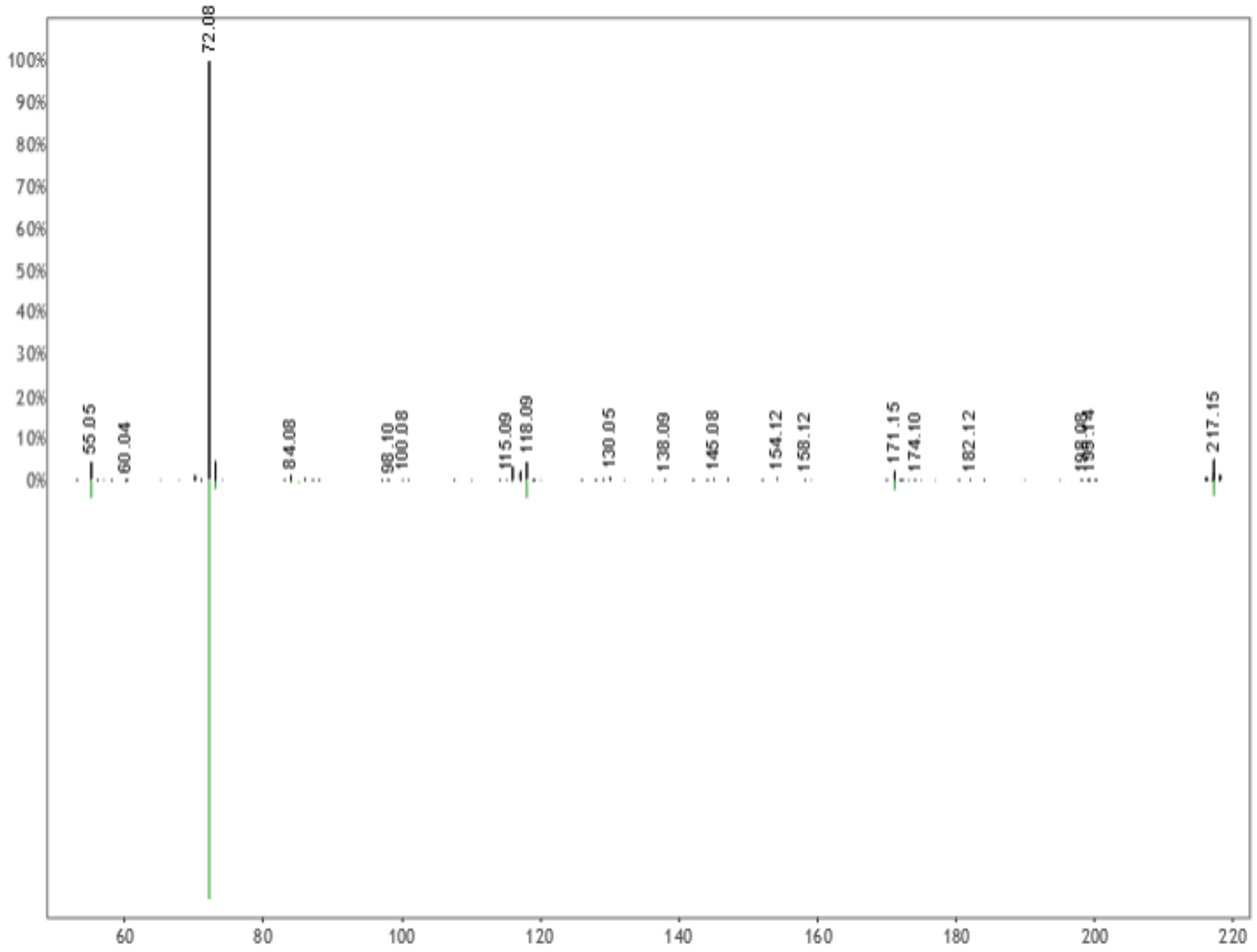
55.05
60.04
72.08
84.08
98.10
100.08
115.09
118.09
130.05
138.09
145.08
154.12
158.12
171.15
174.10
182.12
217.15

o

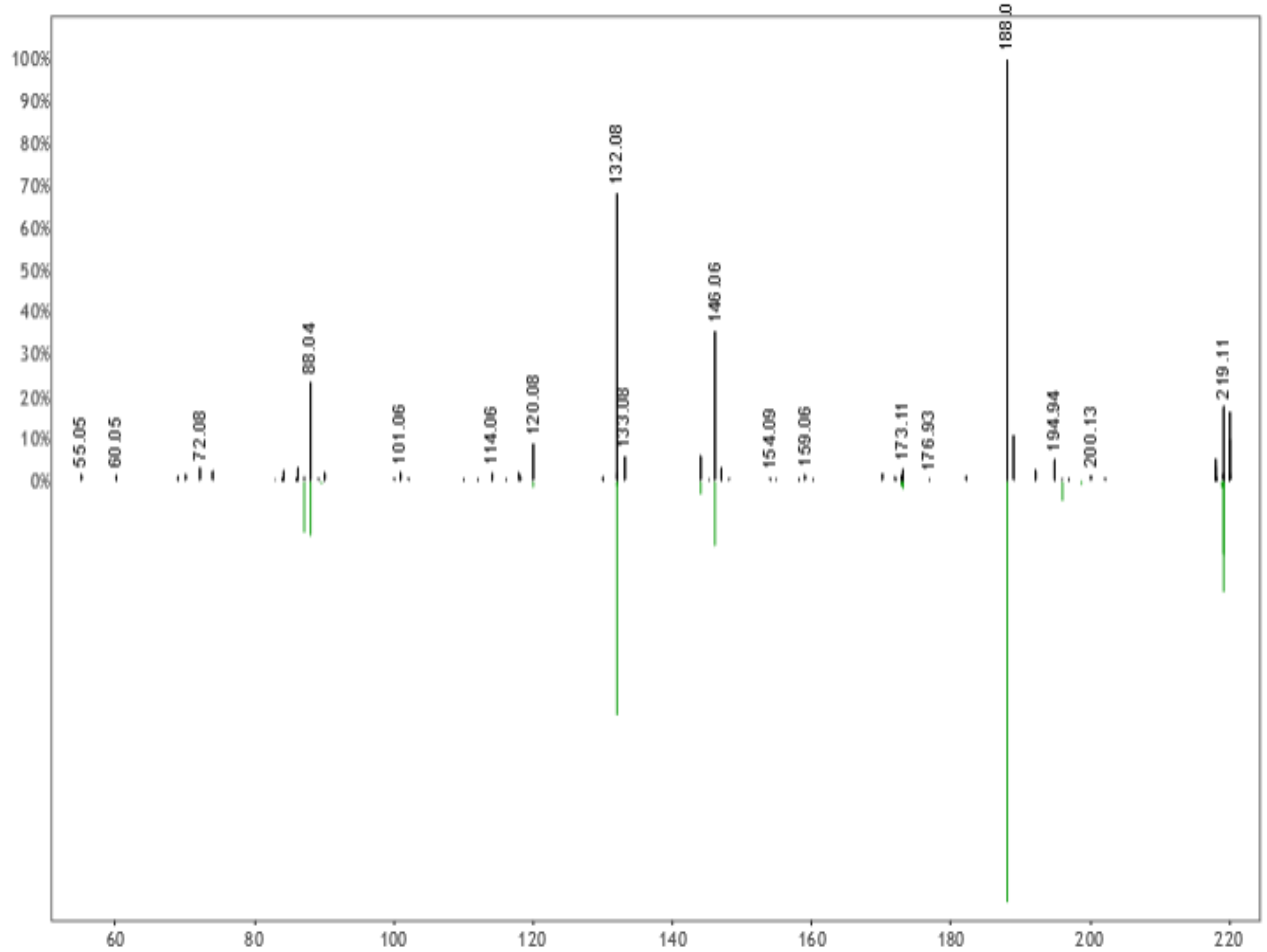
55.05
60.05
72.08
88.04
101.06
114.06
120.08
132.08
133.08
146.06
154.09
159.06
173.11
176.93
188.0
194.94
200.13
219.11

p

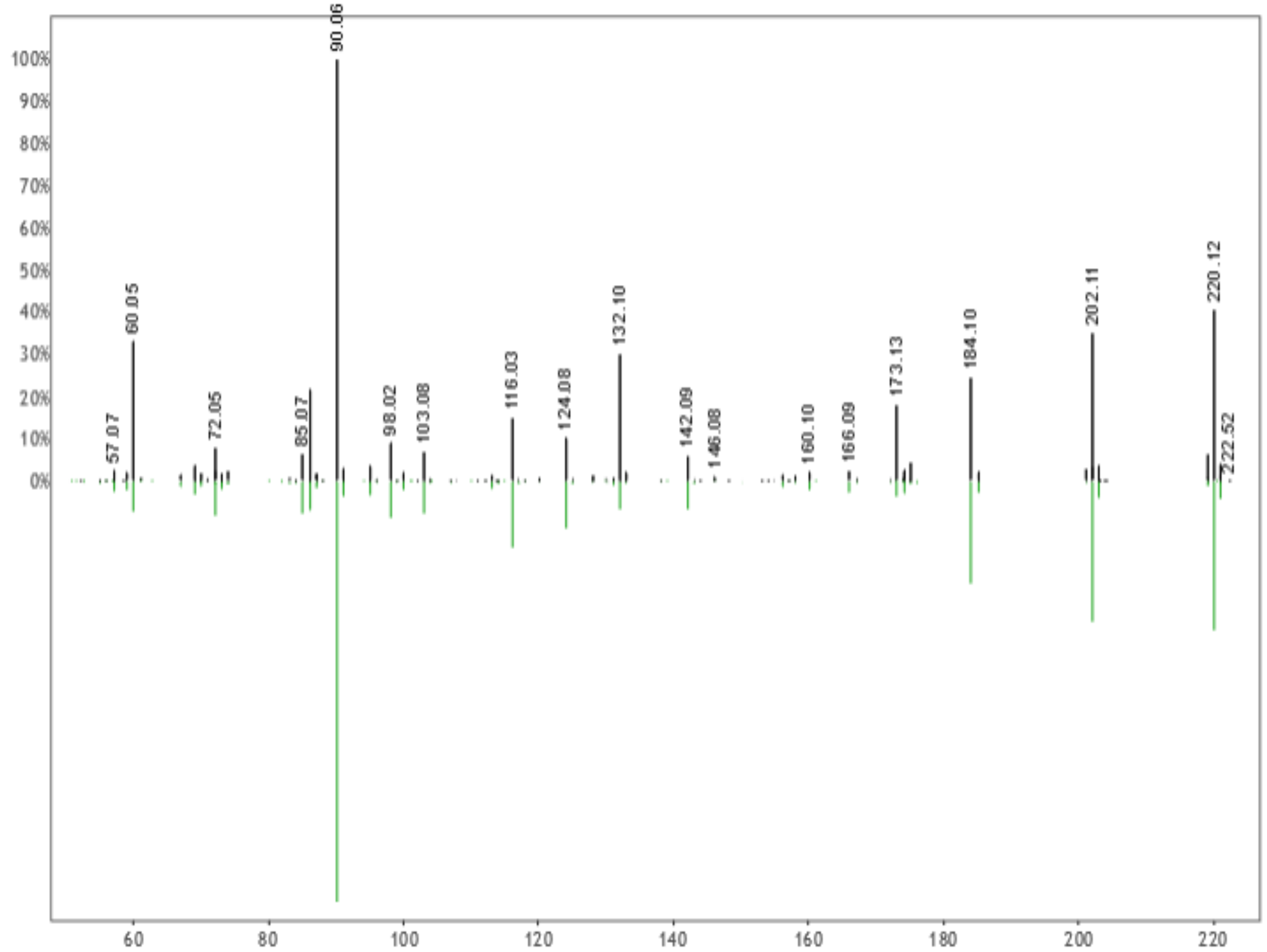
57.07
60.05
72.05
85.07
90.06
98.02
103.08
116.03
124.08
132.10
142.09
146.08
160.10
166.09
173.13
184.10
202.11
220.12
222.52

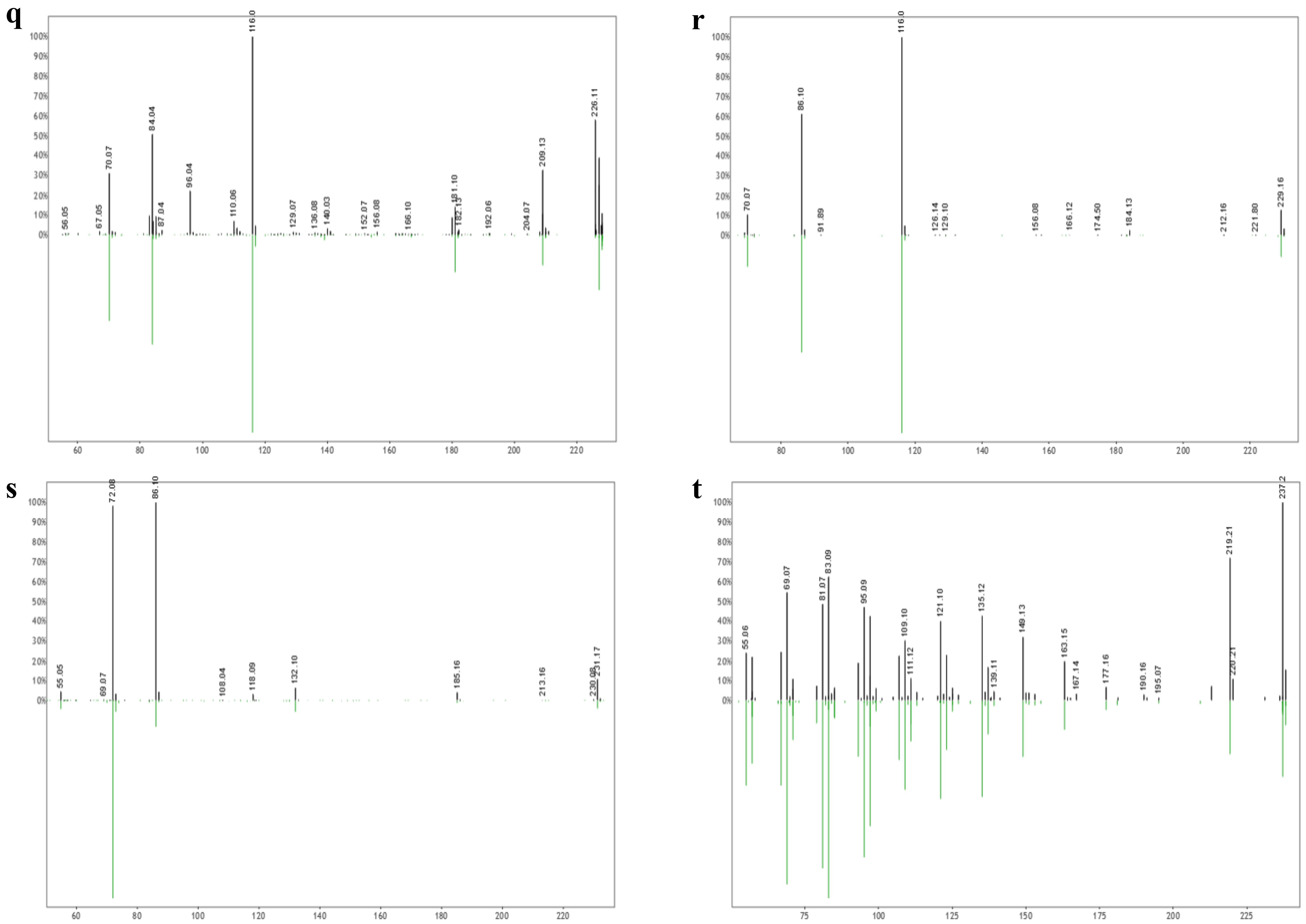

q
116.0
84.04
70.07
96.04
110.06
56.05
67.05
87.04
129.07
136.08
140.03
152.07
156.08
166.10
181.10
182.13
192.06
204.07
209.13
226.11
r
116.0
86.10
70.07
91.89
126.14
129.10
156.08
166.12
174.50
184.13
212.16
221.80
229.16
s
72.08
86.10
55.05
69.07
108.04
118.09
132.10
185.16
213.16
230.08
231.17
t
237.2
219.21
83.09
69.07
81.07
95.09
109.10
121.10
135.12
149.13
55.06
111.12
139.11
163.15
167.14
177.16
190.16
195.07
220.21

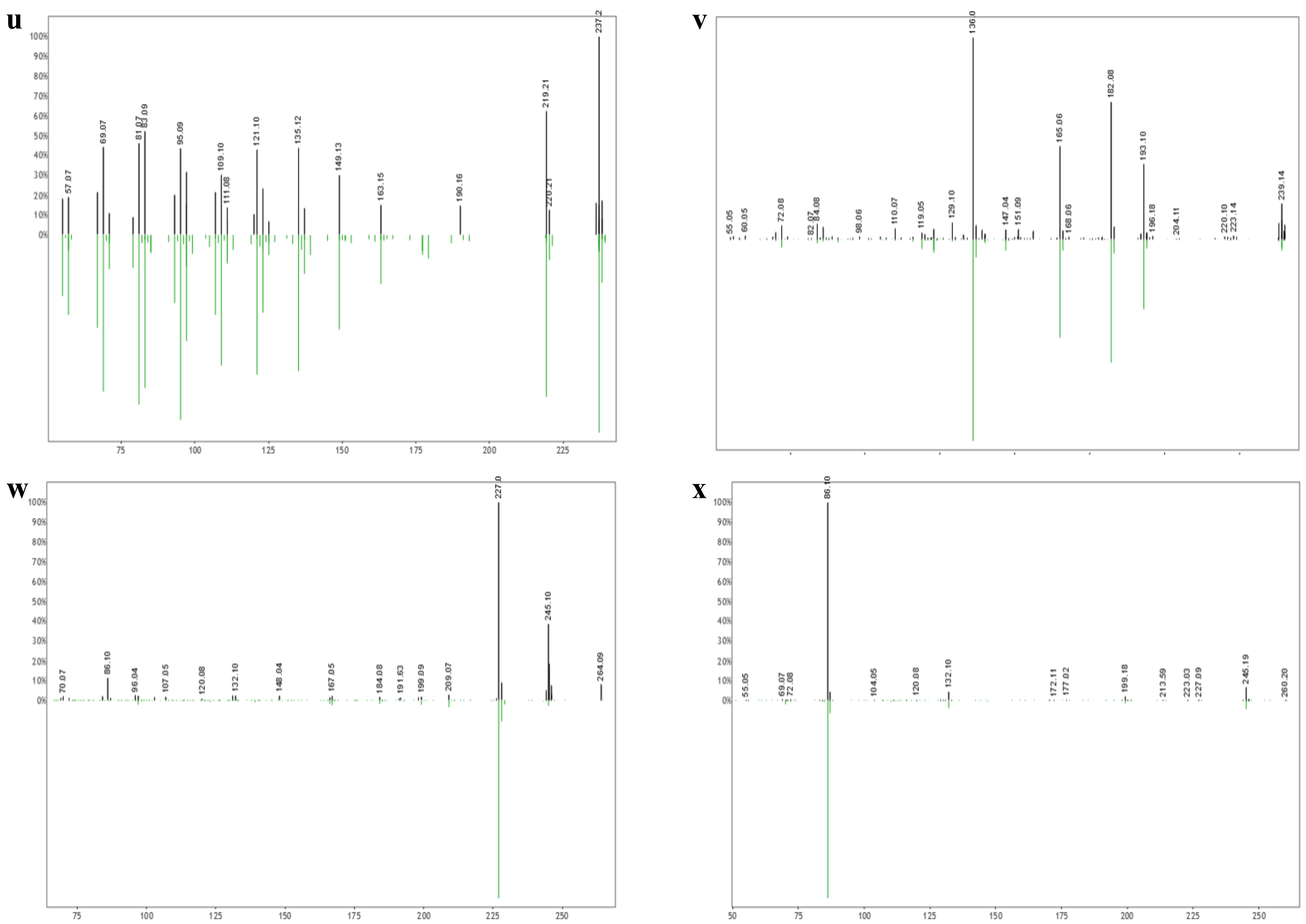
u
100%
90%
80%
70%
60%
50%
40%
30%
20%
10%
0%
57.07
69.07
81.07
83.09
95.09
109.10
111.08
121.10
135.12
149.13
163.15
190.16
219.21
220.21
237.2
75
100
125
150
175
200
225
v
55.05
60.05
72.08
82.07
84.08
98.06
110.07
119.05
129.10
136.0
147.04
151.09
165.06
168.06
182.08
193.10
196.18
204.11
220.10
223.14
239.14
w
100%
90%
80%
70%
60%
50%
40%
30%
20%
10%
0%
70.07
86.10
96.04
107.05
120.08
132.10
148.04
167.05
184.08
191.63
199.09
209.07
227.0
245.10
264.09
75
100
125
150
175
200
225
250
x
100%
90%
80%
70%
60%
50%
40%
30%
20%
10%
0%
55.05
69.07
72.08
86.10
104.05
120.08
132.10
172.11
177.02
199.18
213.59
223.03
227.09
245.19
260.20
50
75
100
125
150
175
200
225
250

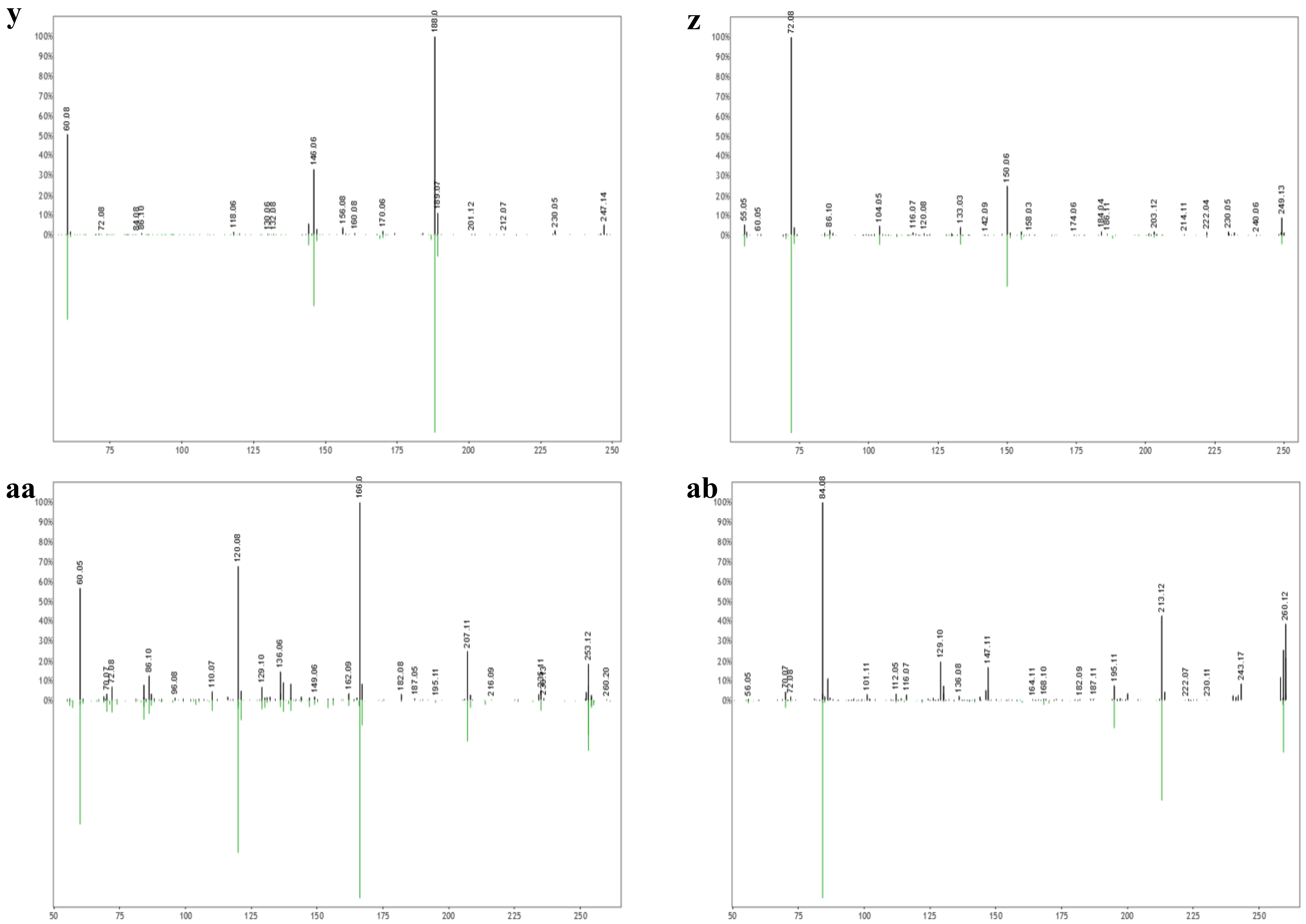

y
188.0
60.08
146.06
189.07
72.08
84.08
86.10
118.06
130.06
132.08
156.08
160.08
170.06
201.12
212.07
230.05
247.14
z
72.08
150.06
55.05
60.05
86.10
104.05
116.07
120.08
133.03
142.09
158.03
174.06
184.04
186.11
203.12
214.11
222.04
230.05
240.06
249.13
aa
166.0
120.08
60.05
70.07
72.08
86.10
96.08
110.07
129.10
136.06
149.06
162.09
182.08
187.05
195.11
207.11
216.09
253.12
260.20
ab
84.08
213.12
260.12
129.10
147.11
56.05
70.07
72.08
101.11
112.05
116.07
136.08
164.11
168.10
182.09
187.11
195.11
222.07
230.11
243.17

ac

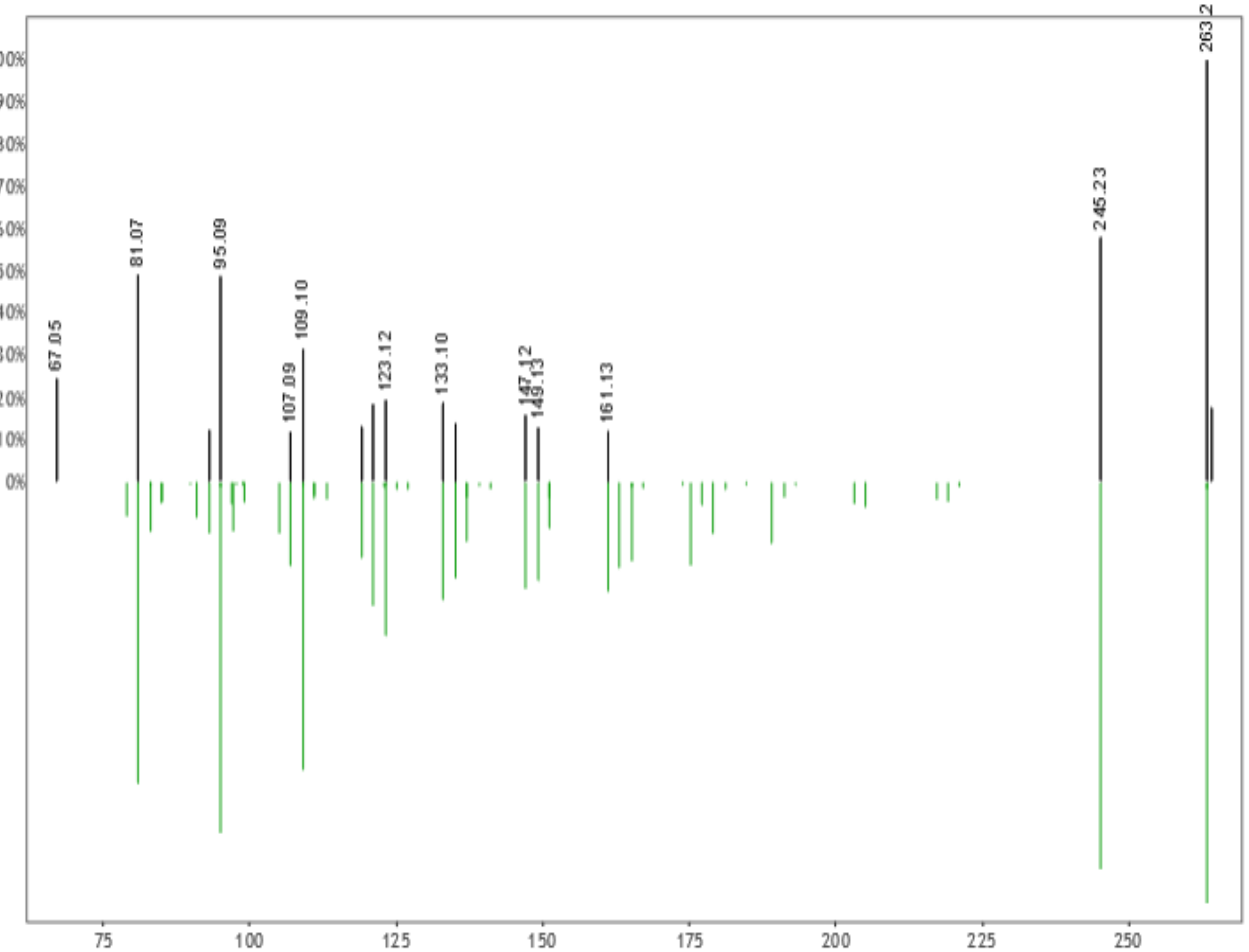
263.2
245.23
81.07
95.09
109.10
67.05
107.09
123.12
133.10
147.12
149.13
161.13
100%
90%
80%
70%
60%
50%
40%
30%
20%
10%
0%
75
100
125
150
175
200
225
250

ad

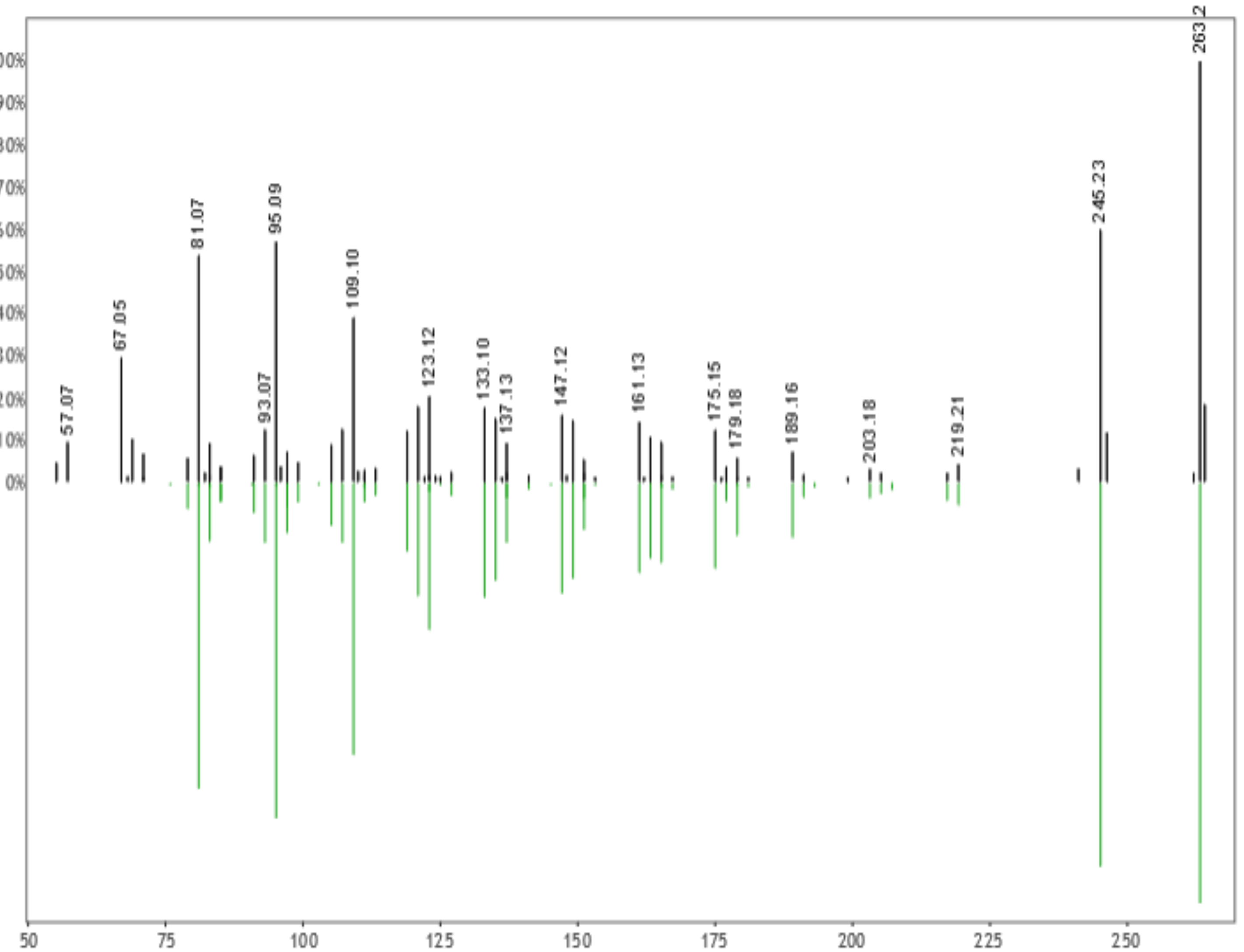
263.2
245.23
81.07
95.09
109.10
67.05
57.07
93.07
123.12
133.10
137.13
147.12
161.13
175.15
179.18
189.16
203.18
219.21
100%
90%
80%
70%
60%
50%
40%
30%
20%
10%
0%
50
75
100
125
150
175
200
225
250

ae

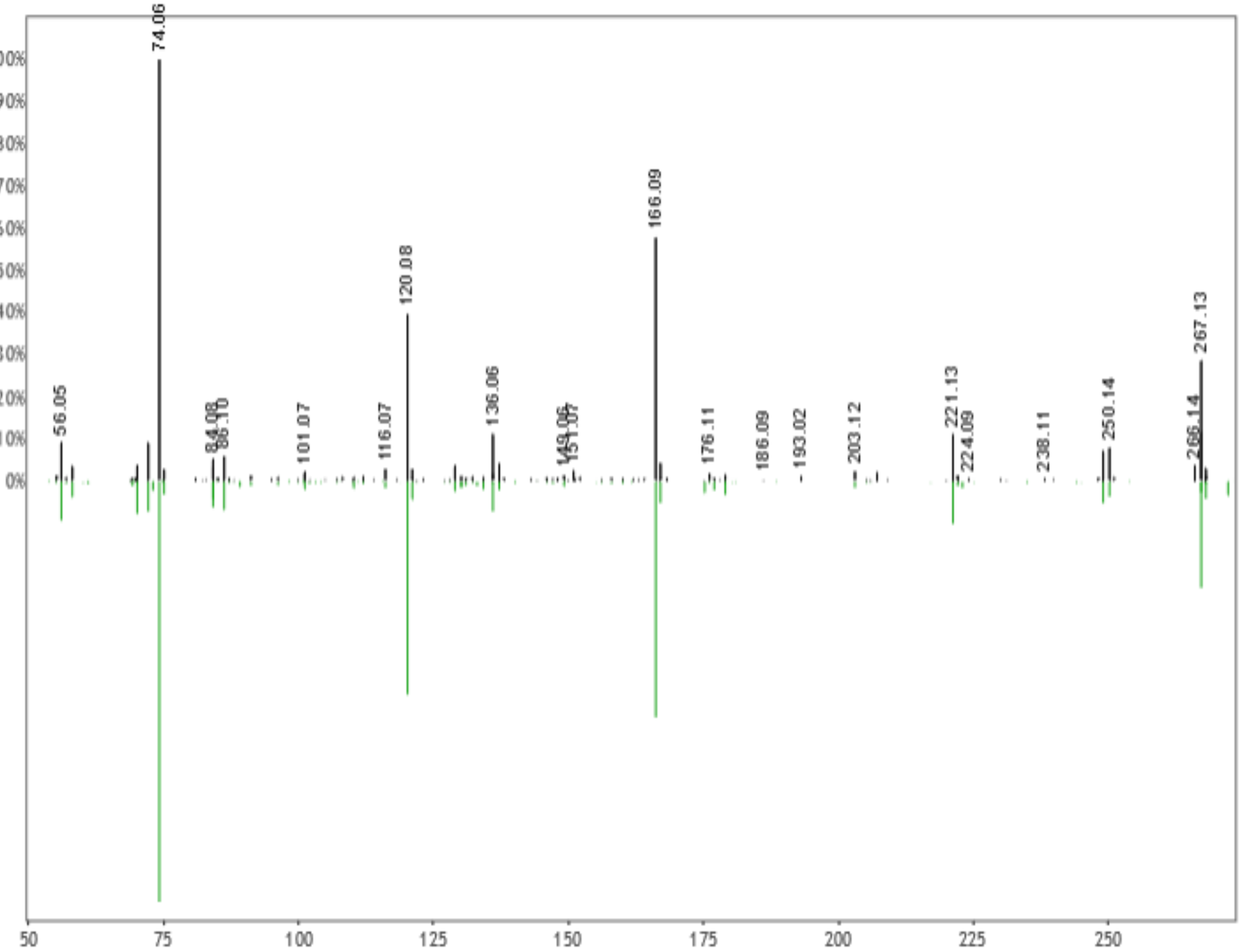
74.06
166.09
120.08
267.13
136.06
56.05
84.08
86.10
101.07
116.07
149.06
151.07
176.11
186.09
193.02
203.12
221.13
224.09
238.11
250.14
266.14
100%
90%
80%
70%
60%
50%
40%
30%
20%
10%
0%
50
75
100
125
150
175
200
225
250

af

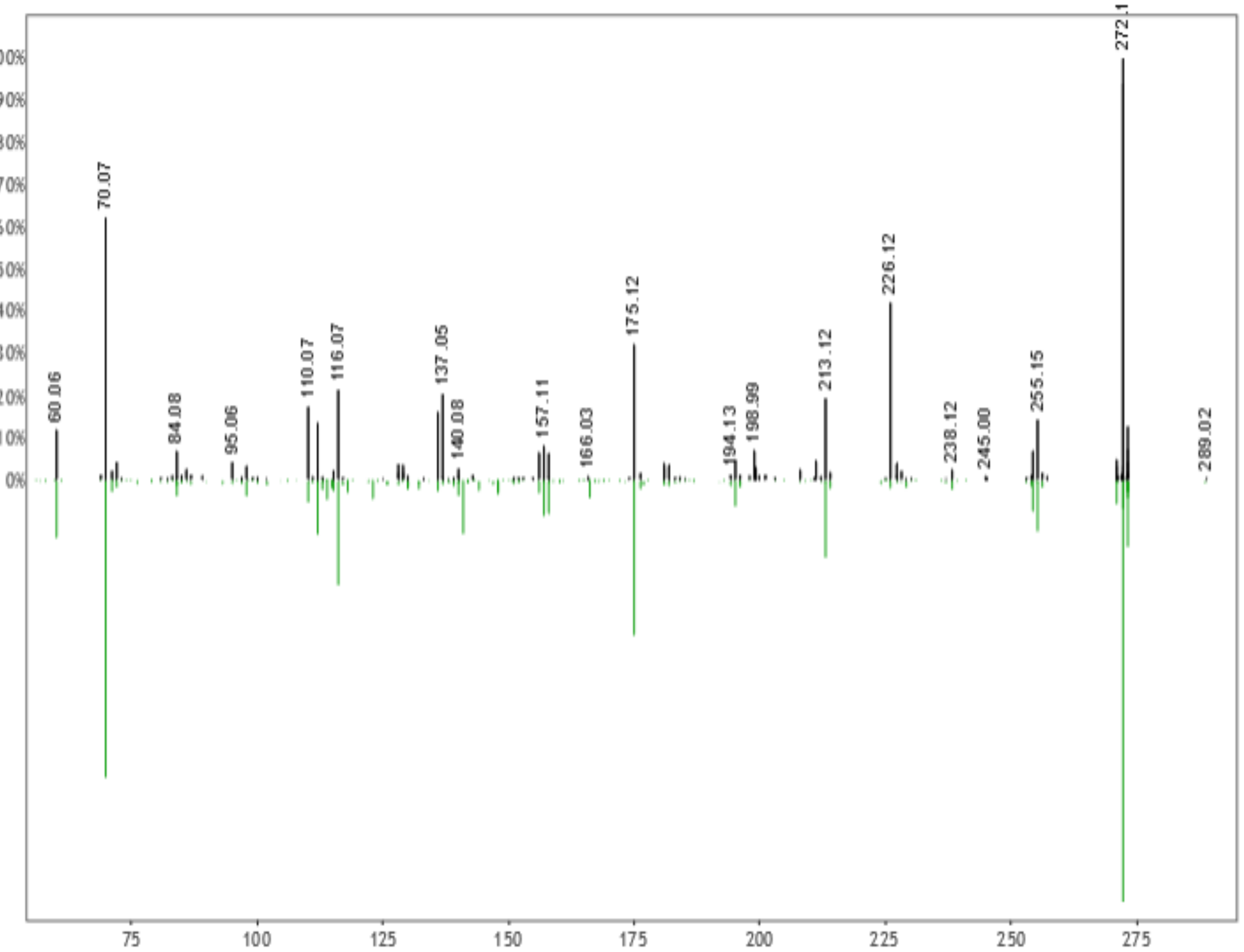
272.1
70.07
226.12
175.12
137.05
116.07
110.07
213.12
255.15
60.06
84.08
95.06
140.08
157.11
166.03
194.13
198.99
238.12
245.00
289.02
100%
90%
80%
70%
60%
50%
40%
30%
20%
10%
0%
75
100
125
150
175
200
225
250
275

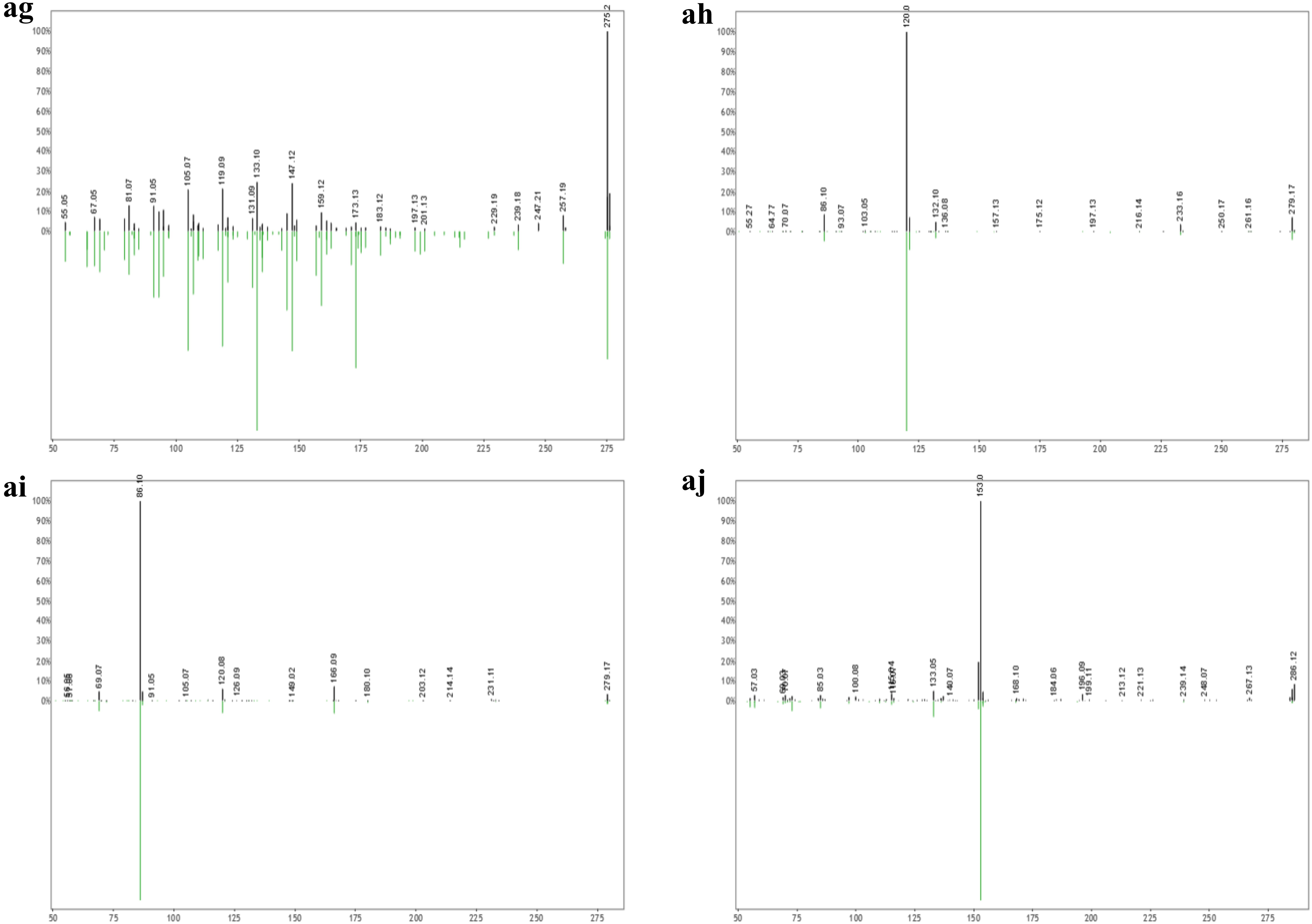
ag
275.2
105.07
119.09
133.10
147.12
131.09
55.05
67.05
81.07
91.05
159.12
173.13
183.12
197.13
201.13
229.19
239.18
247.21
257.19
ah
120.0
55.27
64.77
70.07
86.10
93.07
103.05
132.10
136.08
157.13
175.12
197.13
216.14
233.16
250.17
261.16
279.17
ai
86.10
69.07
91.05
105.07
120.08
126.09
149.02
166.09
180.10
203.12
214.14
231.11
279.17
aj
153.0
57.03
85.03
100.08
133.05
140.07
168.10
184.06
196.09
199.11
213.12
221.13
239.14
248.07
267.13
286.12

ak

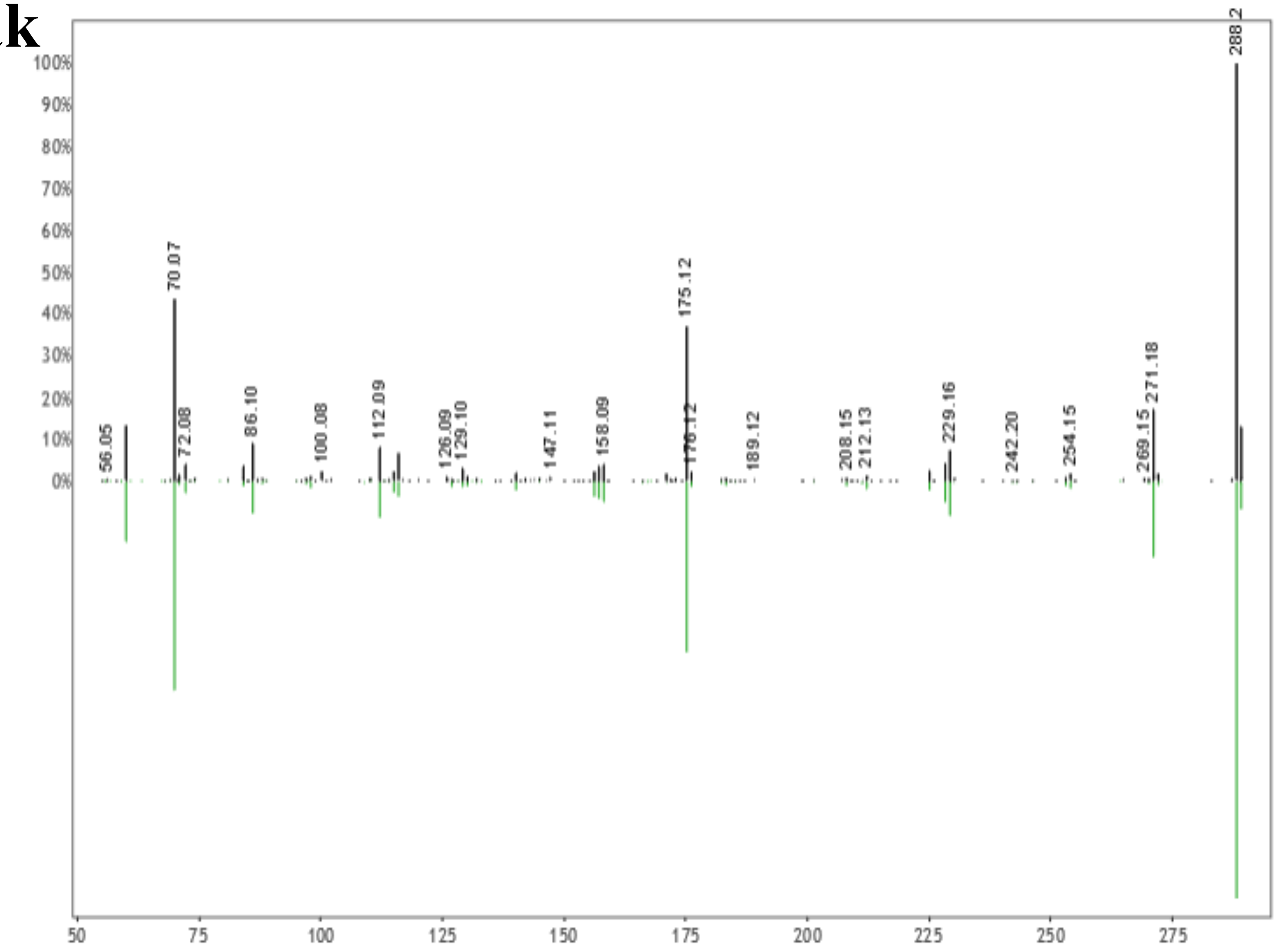
288.2
70.07
175.12
271.18
229.16
112.09
158.09
86.10
72.08
100.08
126.09
129.10
147.11
189.12
208.15
212.13
242.20
254.15
269.15
56.05
100%
90%
80%
70%
60%
50%
40%
30%
20%
10%
0%
50
75
100
125
150
175
200
225
250
275

al

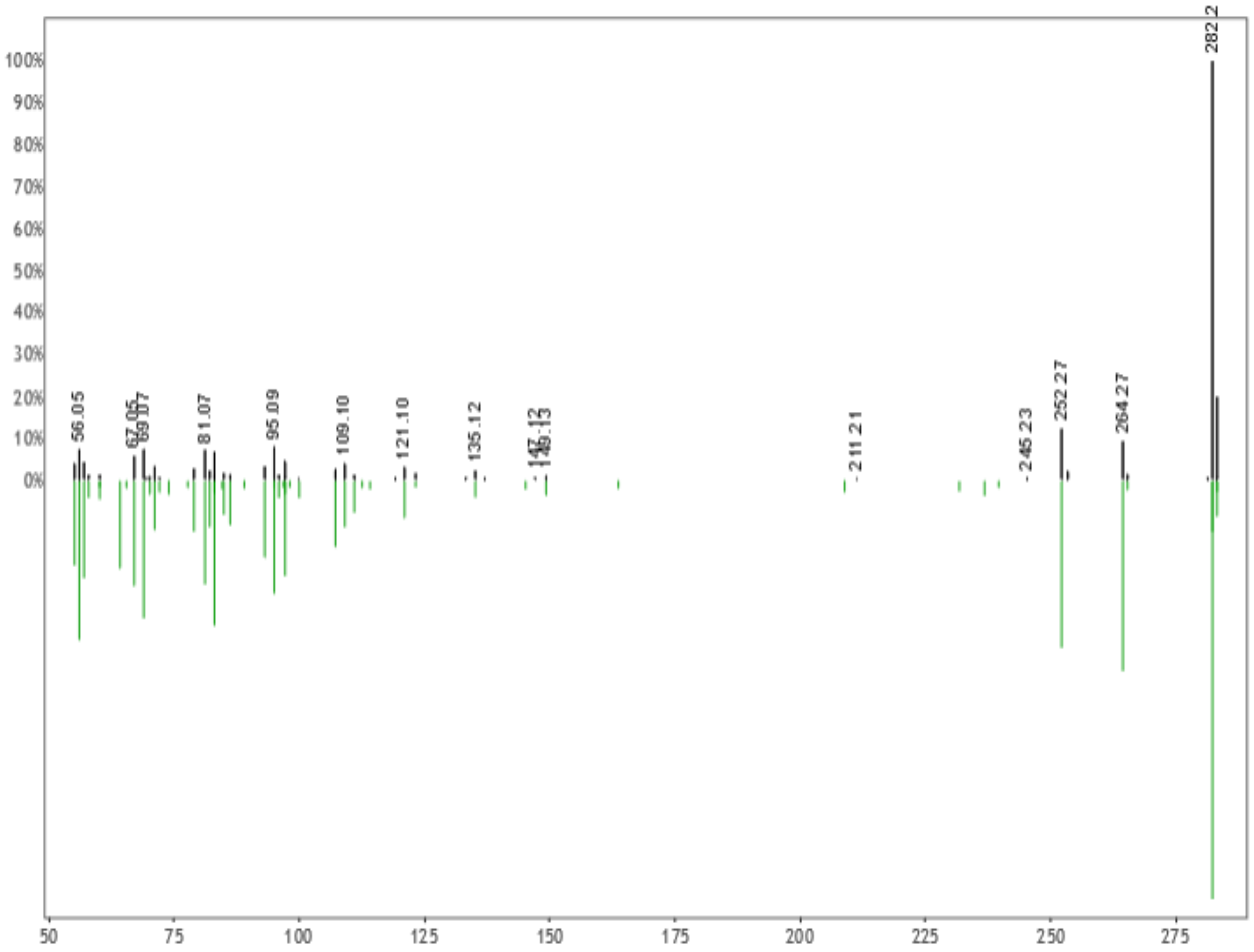
282.2
252.27
264.27
56.05
81.07
95.09
109.10
121.10
135.12
211.21
245.23
100%
90%
80%
70%
60%
50%
40%
30%
20%
10%
0%
50
75
100
125
150
175
200
225
250
275

am

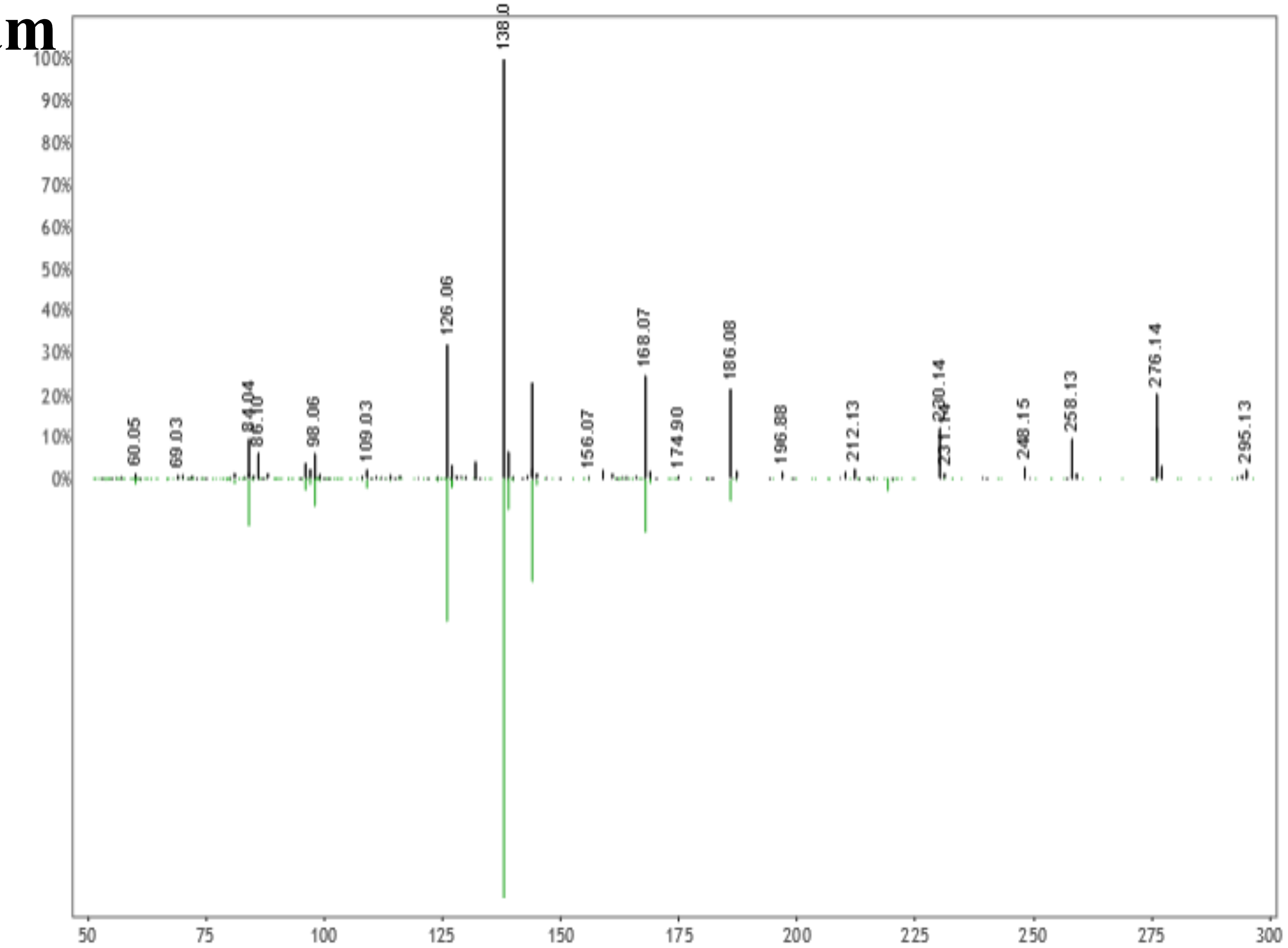
138.0
126.06
168.07
186.08
276.14
258.13
248.15
212.13
196.88
174.90
156.07
109.03
98.06
60.05
69.03
295.13
100%
90%
80%
70%
60%
50%
40%
30%
20%
10%
0%
50
75
100
125
150
175
200
225
250
275
300

an

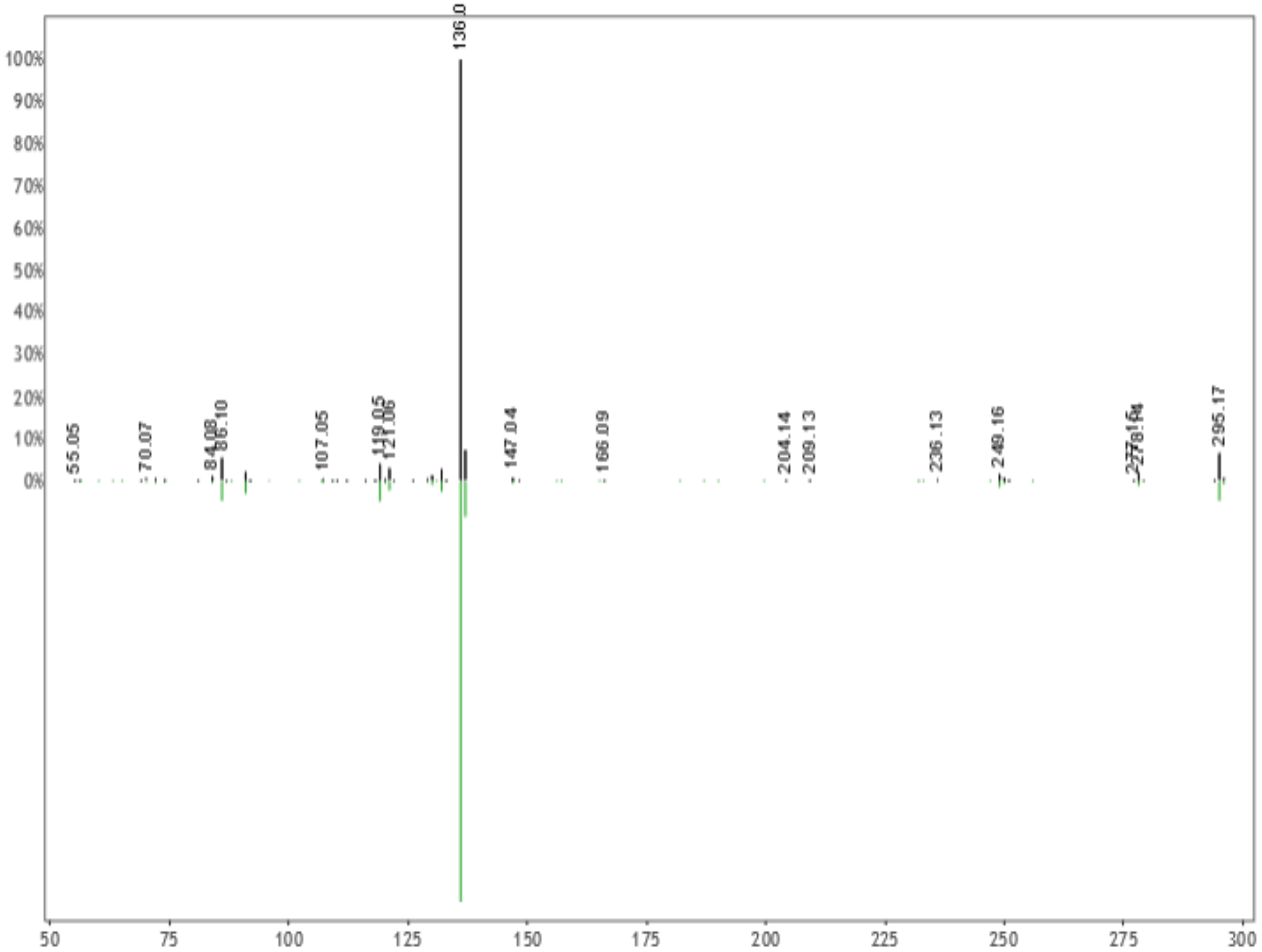
136.0
295.17
55.05
70.07
107.05
147.04
166.09
204.14
209.13
236.13
100%
90%
80%
70%
60%
50%
40%
30%
20%
10%
0%
50
75
100
125
150
175
200
225
250
275
300

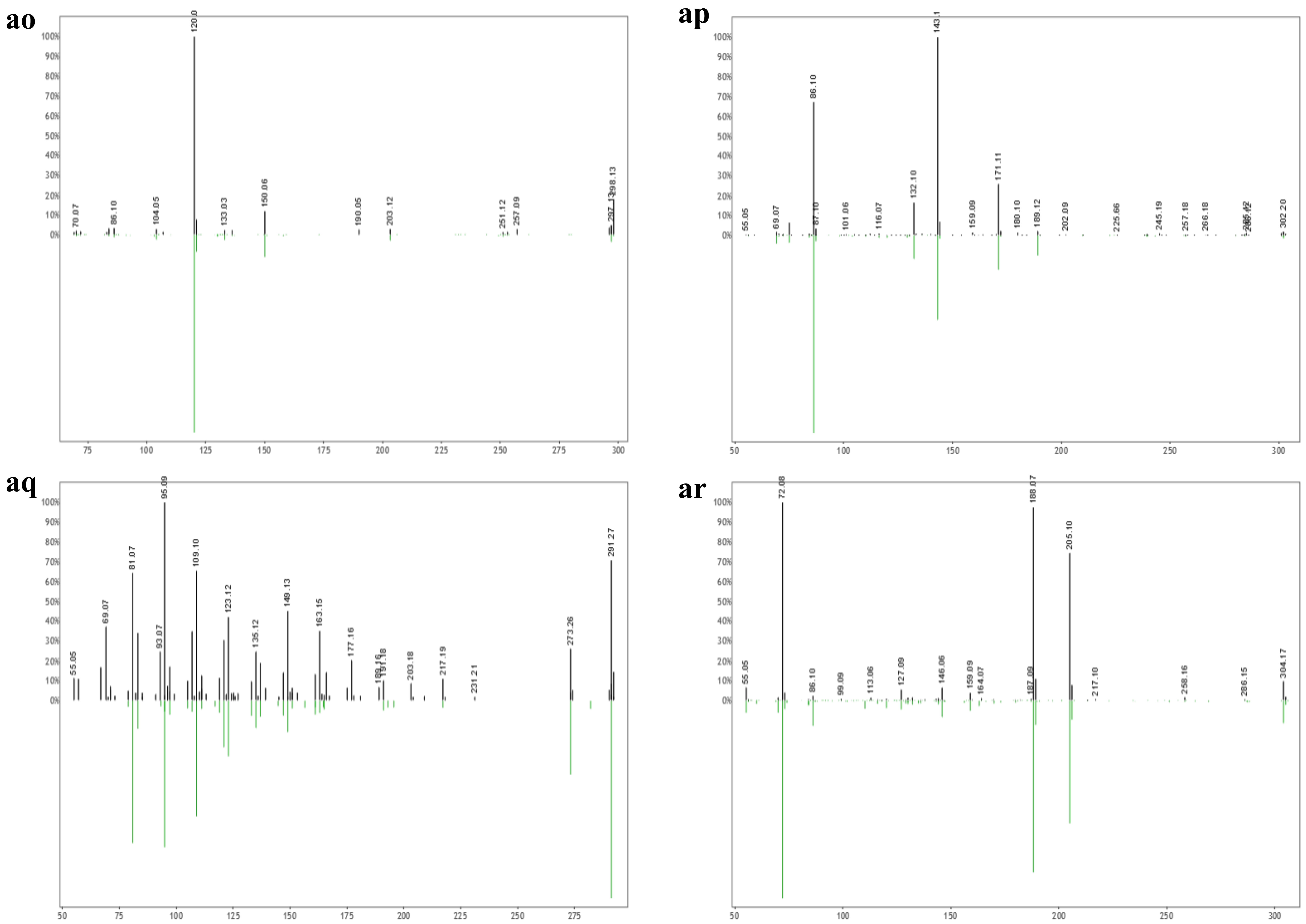
ao
120.0
70.07
86.10
104.05
133.03
150.06
190.05
203.12
251.12
257.09
298.13
ap
143.1
86.10
55.05
69.07
87.10
101.06
116.07
132.10
159.09
171.11
180.10
189.12
202.09
225.66
245.19
257.18
266.18
302.20
aq
95.09
55.05
69.07
81.07
93.07
109.10
123.12
135.12
149.13
163.15
177.16
191.18
203.18
217.19
231.21
273.26
291.27
ar
72.08
188.07
205.10
55.05
86.10
99.09
113.06
127.09
146.06
159.09
164.07
187.09
217.10
258.16
286.15
304.17

as

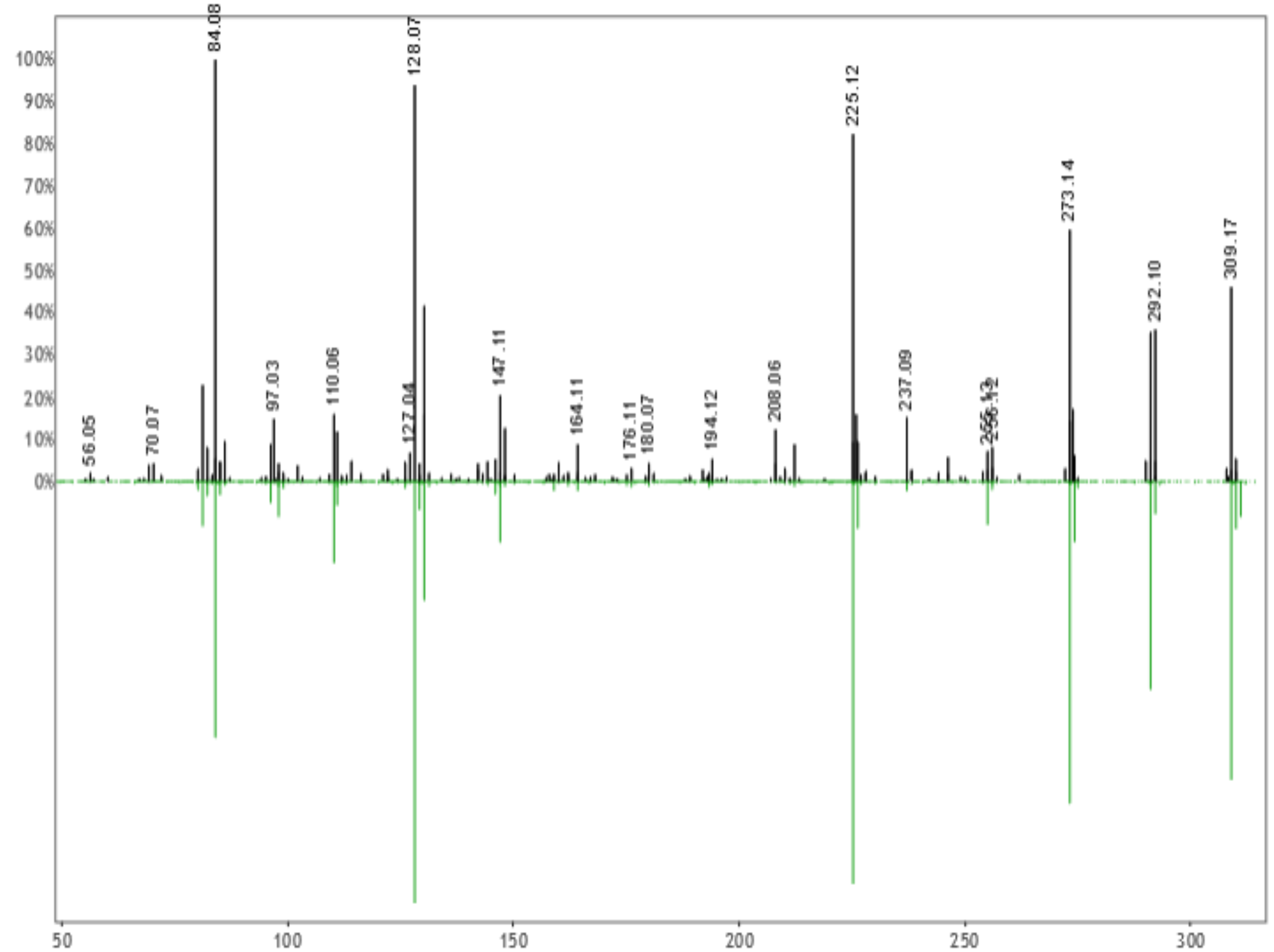
56.05
70.07
84.08
97.03
110.06
127.04
128.07
147.11
164.11
176.11
180.07
194.12
208.06
225.12
237.09
273.14
292.10
309.17
100%
90%
80%
70%
60%
50%
40%
30%
20%
10%
0%
50
100
150
200
250
300

at

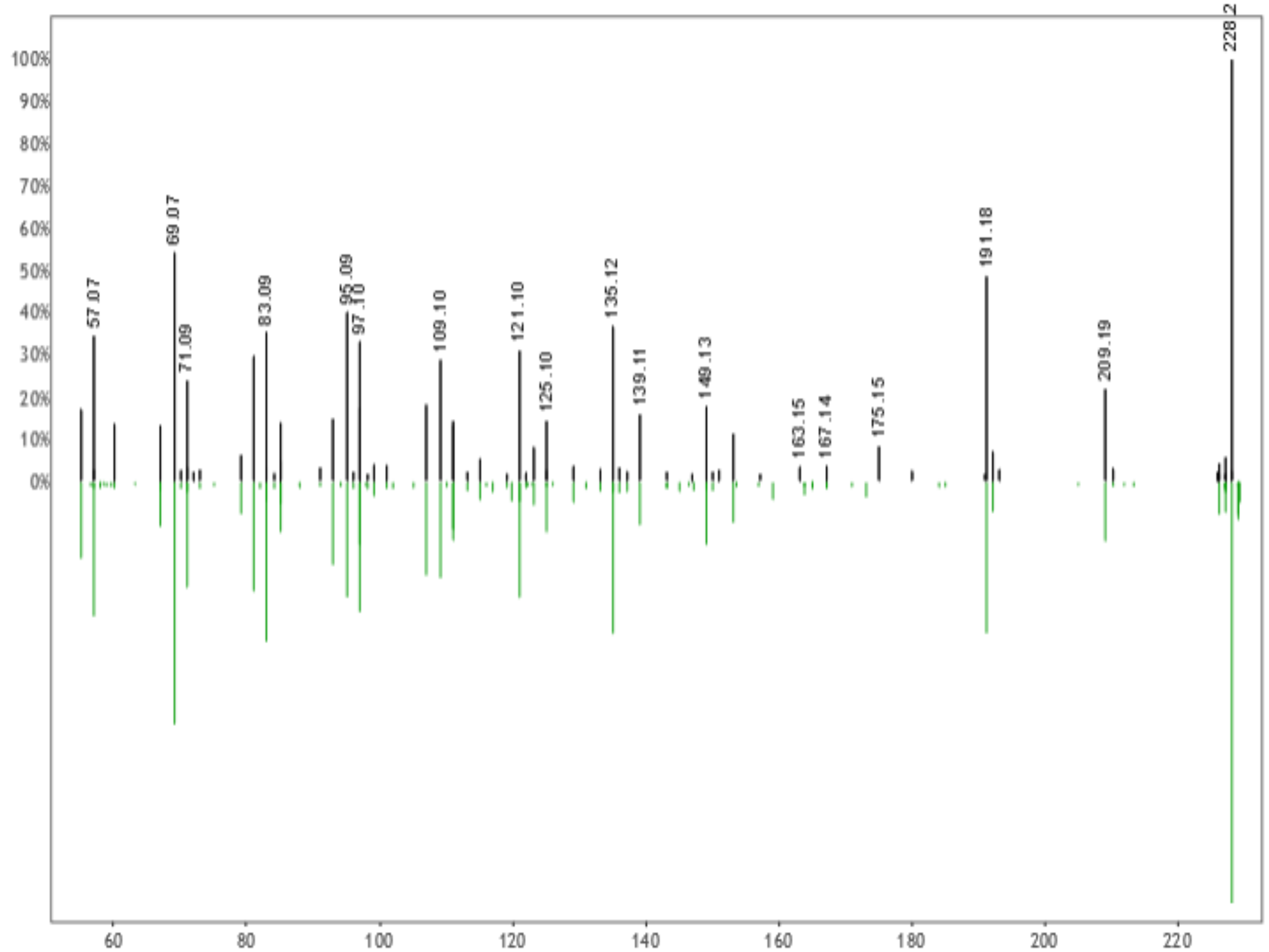
57.07
69.07
71.09
83.09
95.09
97.10
109.10
121.10
125.10
135.12
139.11
149.13
163.15
167.14
175.15
191.18
209.19
228.2
100%
90%
80%
70%
60%
50%
40%
30%
20%
10%
0%
60
80
100
120
140
160
180
200
220

au

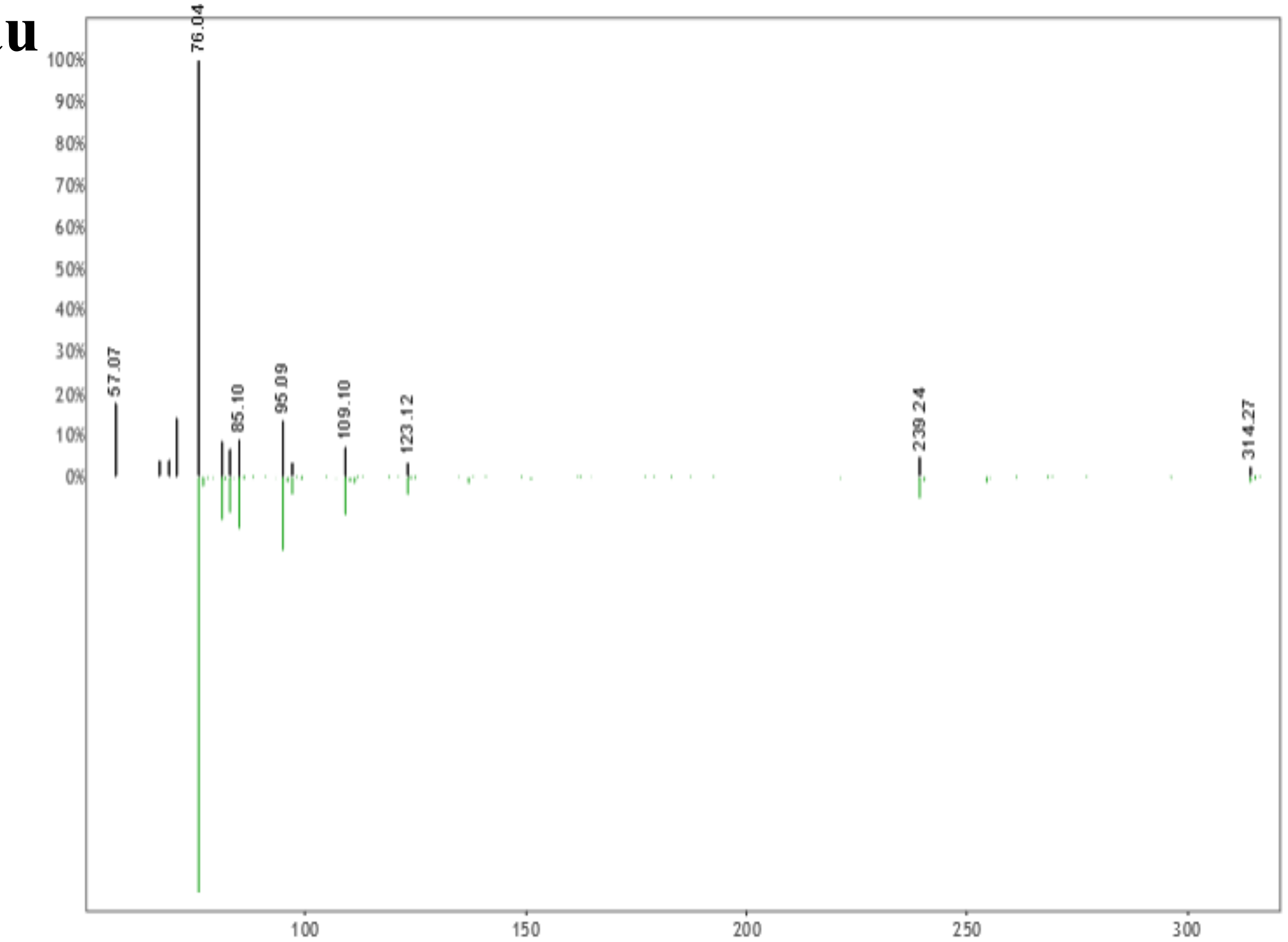
57.07
76.04
85.10
95.09
109.10
123.12
239.24
314.27
100%
90%
80%
70%
60%
50%
40%
30%
20%
10%
0%
100
150
200
250
300

av

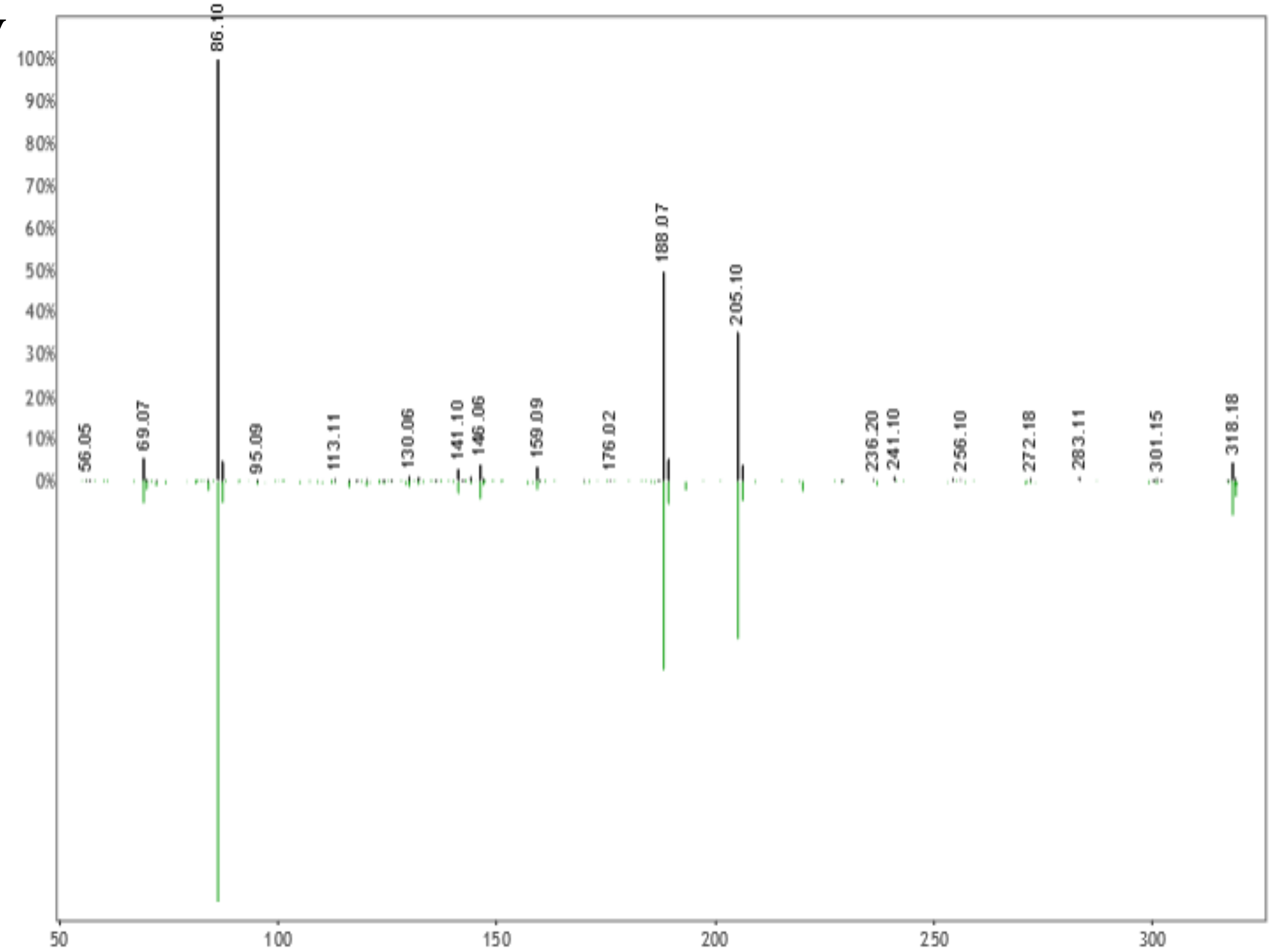
56.05
69.07
86.10
95.09
113.11
130.06
141.10
146.06
159.09
176.02
188.07
205.10
236.20
241.10
256.10
272.18
283.11
301.15
318.18
100%
90%
80%
70%
60%
50%
40%
30%
20%
10%
0%
50
100
150
200
250
300

aw

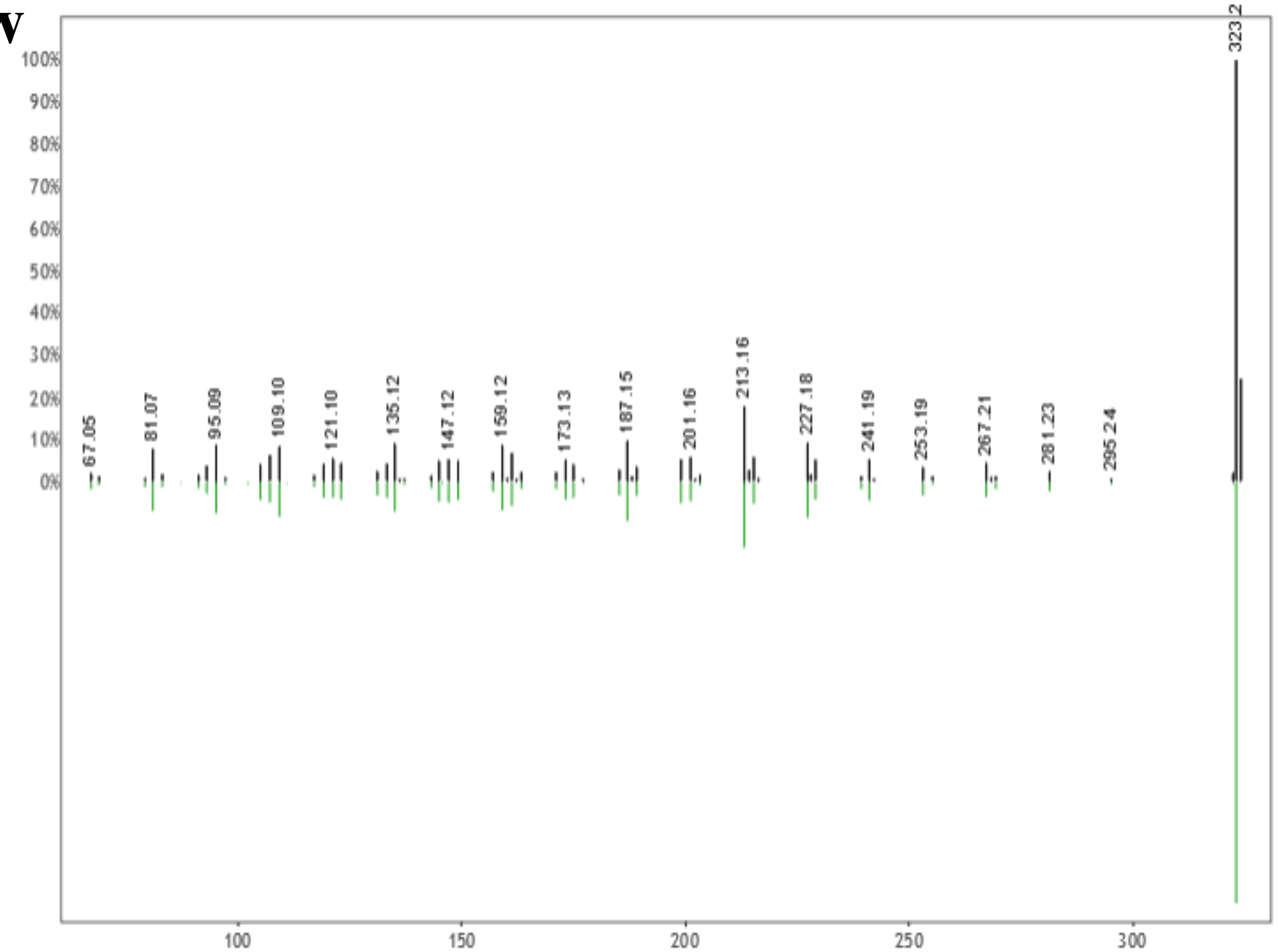
323.2
100%
90%
80%
70%
60%
50%
40%
30%
20%
10%
0%
67.05
81.07
95.09
109.10
121.10
135.12
147.12
159.12
173.13
187.15
201.16
213.16
227.18
241.19
253.19
267.21
281.23
295.24
100
150
200
250
300

ax

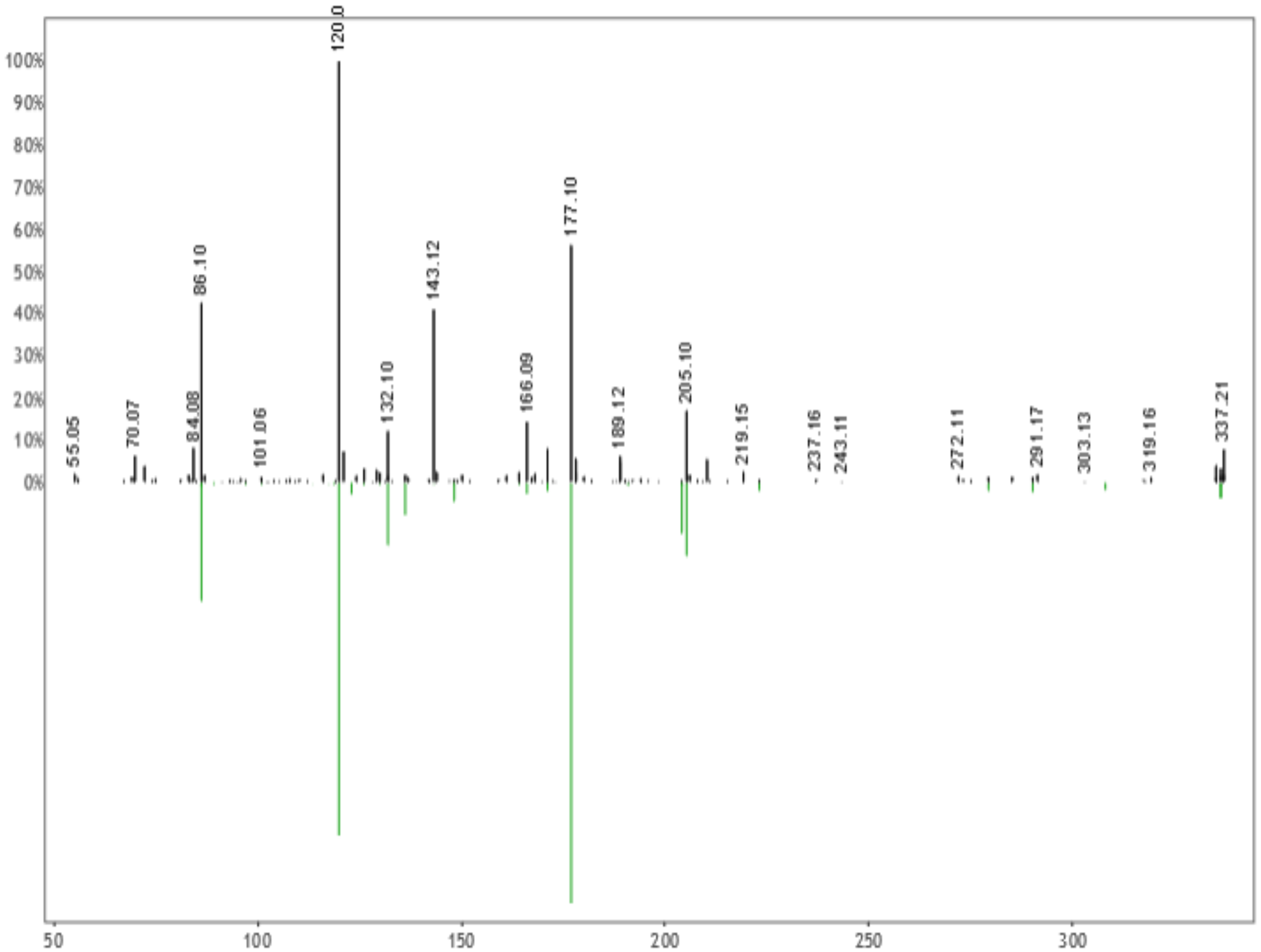
120.0
100%
90%
80%
70%
60%
50%
40%
30%
20%
10%
0%
55.05
70.07
84.08
86.10
101.06
132.10
143.12
166.09
177.10
189.12
205.10
219.15
237.16
243.11
272.11
291.17
303.13
319.16
337.21
50
100
150
200
250
300

ay

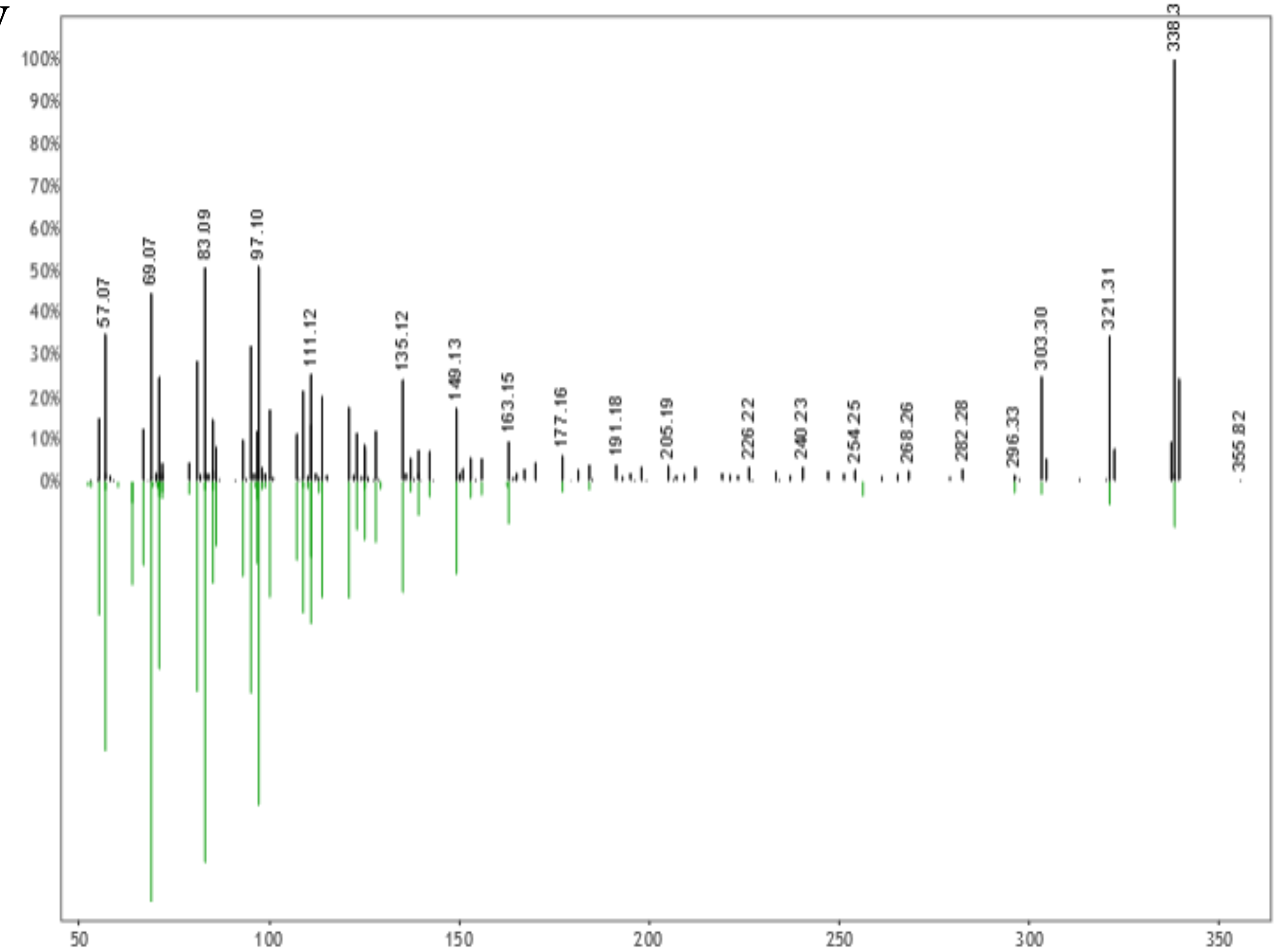
338.3
100%
90%
80%
70%
60%
50%
40%
30%
20%
10%
0%
57.07
69.07
83.09
97.10
111.12
135.12
149.13
163.15
177.16
191.18
205.19
226.22
240.23
254.25
268.26
282.28
296.33
303.30
321.31
355.82
50
100
150
200
250
300
350

az

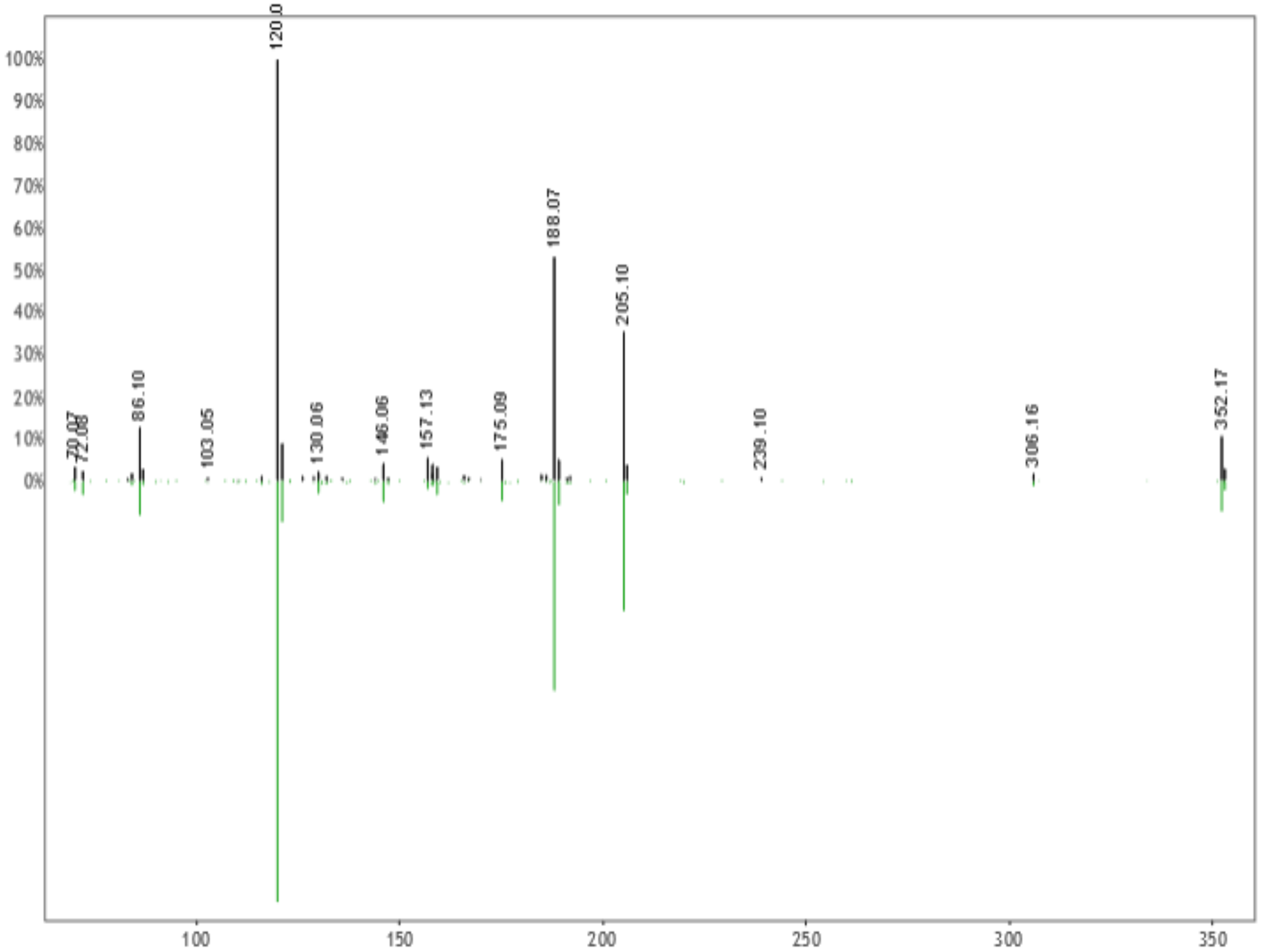
120.0
100%
90%
80%
70%
60%
50%
40%
30%
20%
10%
0%
70.07
72.08
86.10
103.05
130.06
146.06
157.13
175.09
188.07
205.10
239.10
306.16
352.17
100
150
200
250
300
350

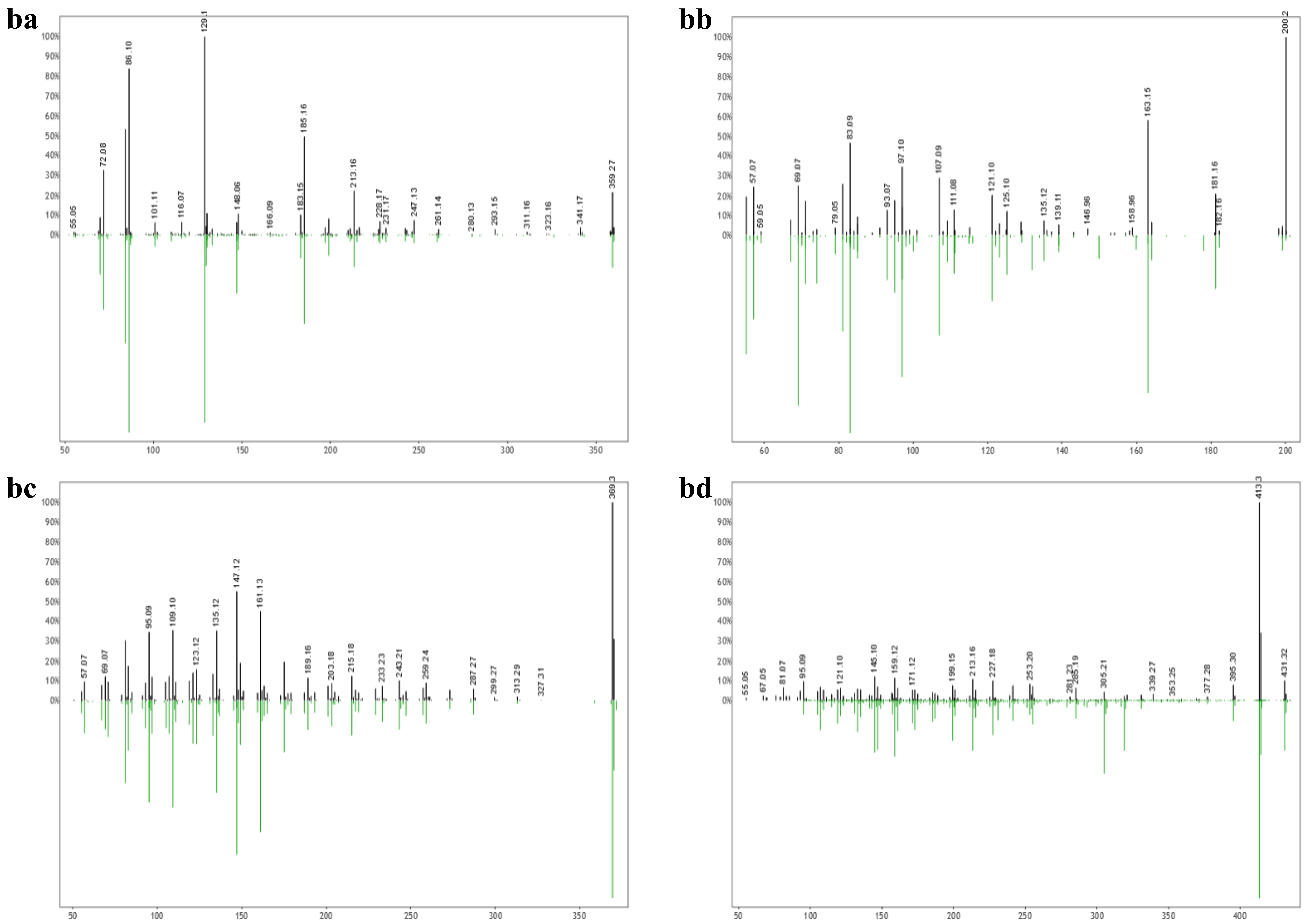
ba
55.05
72.08
86.10
101.11
116.07
129.1
148.06
166.09
183.15
185.16
213.16
228.17
231.17
247.13
261.14
280.13
293.15
311.16
323.16
341.17
359.27
bb
57.07
59.05
69.07
79.05
83.09
93.07
97.10
107.09
111.08
121.10
125.10
135.12
139.11
146.96
158.96
163.15
181.16
182.16
200.2
bc
57.07
69.07
95.09
109.10
123.12
135.12
147.12
161.13
189.16
203.18
215.18
233.23
243.21
259.24
287.27
299.27
313.29
327.31
369.3
bd
55.05
67.05
81.07
95.09
121.10
145.10
159.12
171.12
199.15
213.16
227.18
253.20
281.23
285.19
305.21
339.27
353.25
377.28
395.30
413.3
431.32

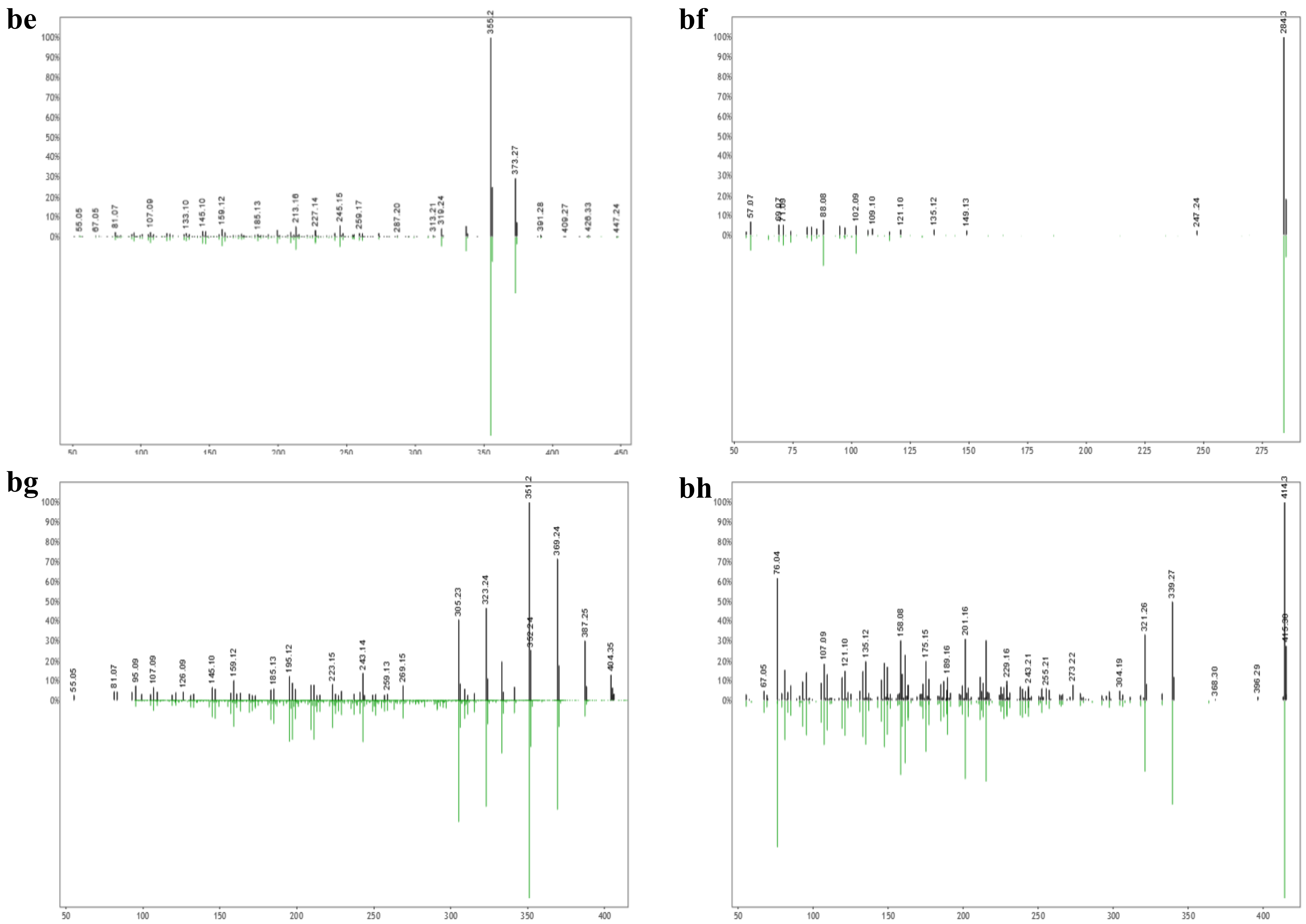
be
355.2
373.27
55.05
67.05
81.07
107.09
133.10
145.10
159.12
185.13
213.16
227.14
245.15
259.17
287.20
313.21
319.24
391.28
409.27
426.33
447.24
bf
284.3
57.07
88.08
102.09
109.10
121.10
135.12
149.13
247.24
bg
351.2
369.24
323.24
305.23
352.24
387.25
404.35
55.05
81.07
95.09
107.09
126.09
145.10
159.12
185.13
195.12
223.15
243.14
259.13
269.15
bh
414.3
76.04
339.27
321.26
201.16
158.08
175.15
135.12
121.10
107.09
189.16
229.16
243.21
255.21
273.22
304.19
67.05
368.30
396.29

bi

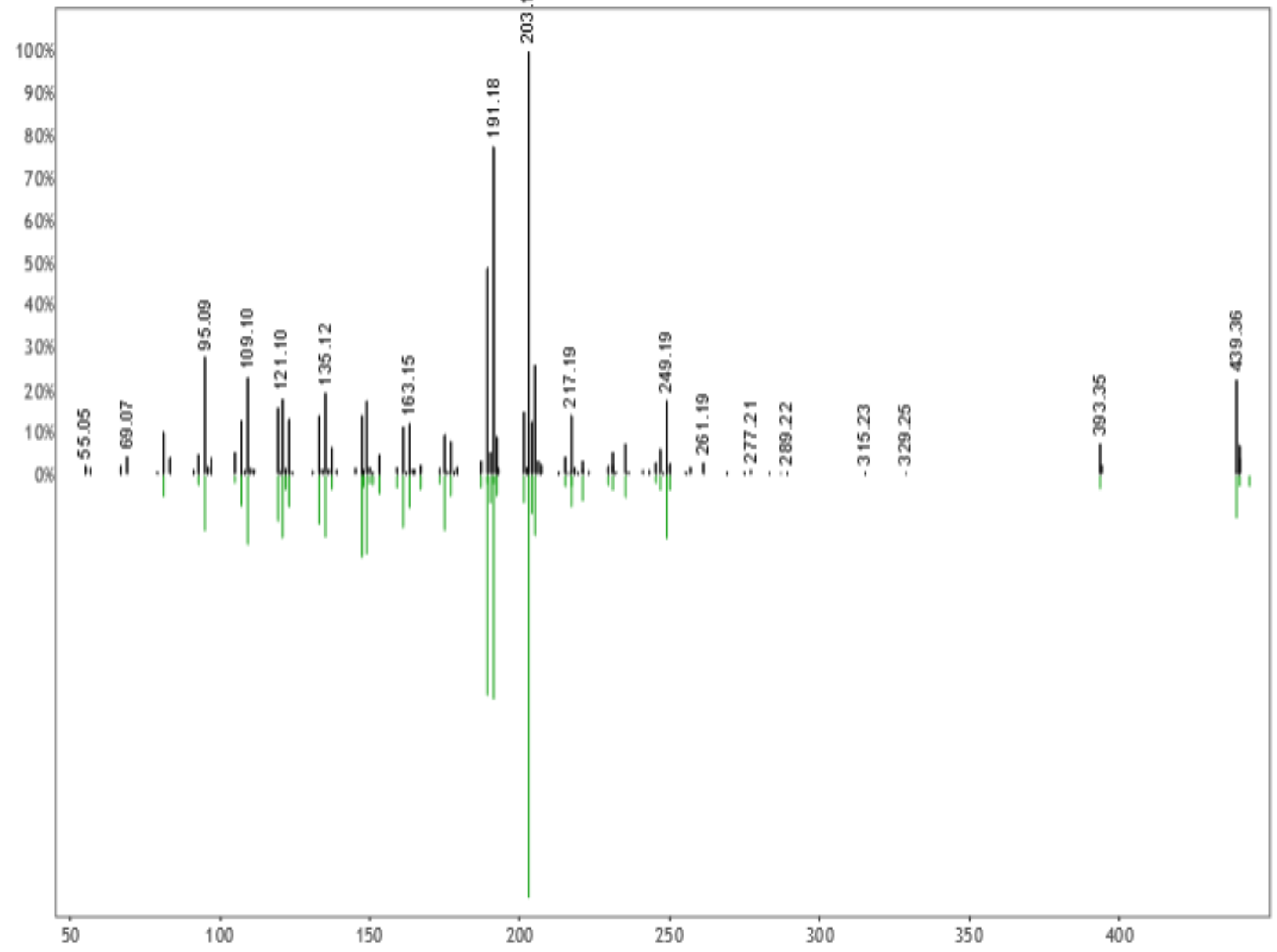
100%
90%
80%
70%
60%
50%
40%
30%
20%
10%
0%
55.05
69.07
95.09
109.10
121.10
135.12
163.15
191.18
203.1
217.19
249.19
261.19
277.21
289.22
315.23
329.25
393.35
439.36
50
100
150
200
250
300
350
400

bj

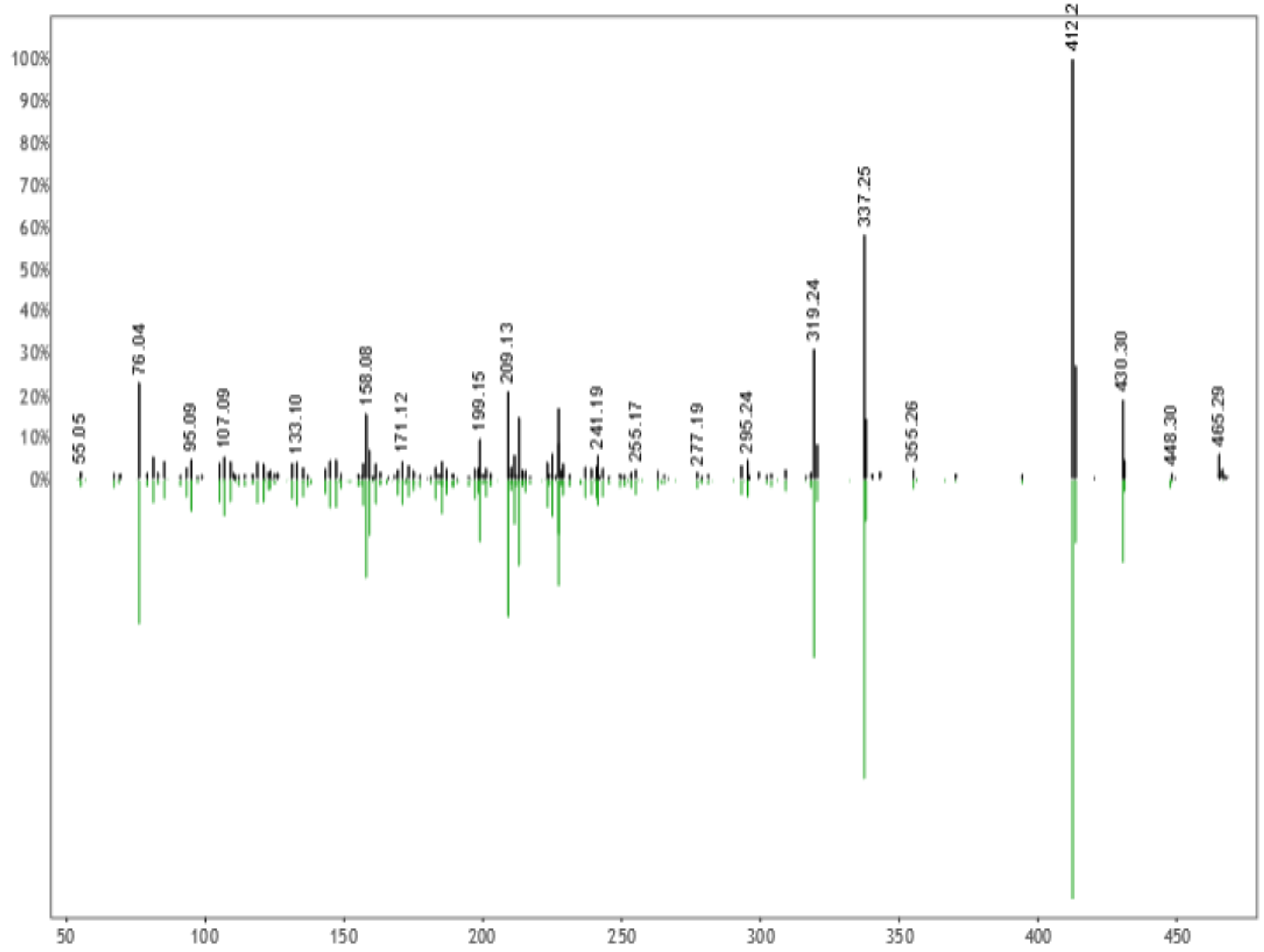
100%
90%
80%
70%
60%
50%
40%
30%
20%
10%
0%
55.05
76.04
95.09
107.09
133.10
158.08
171.12
199.15
209.13
241.19
255.17
277.19
295.24
319.24
337.25
355.26
412.2
430.30
448.30
465.29
50
100
150
200
250
300
350
400
450

bk

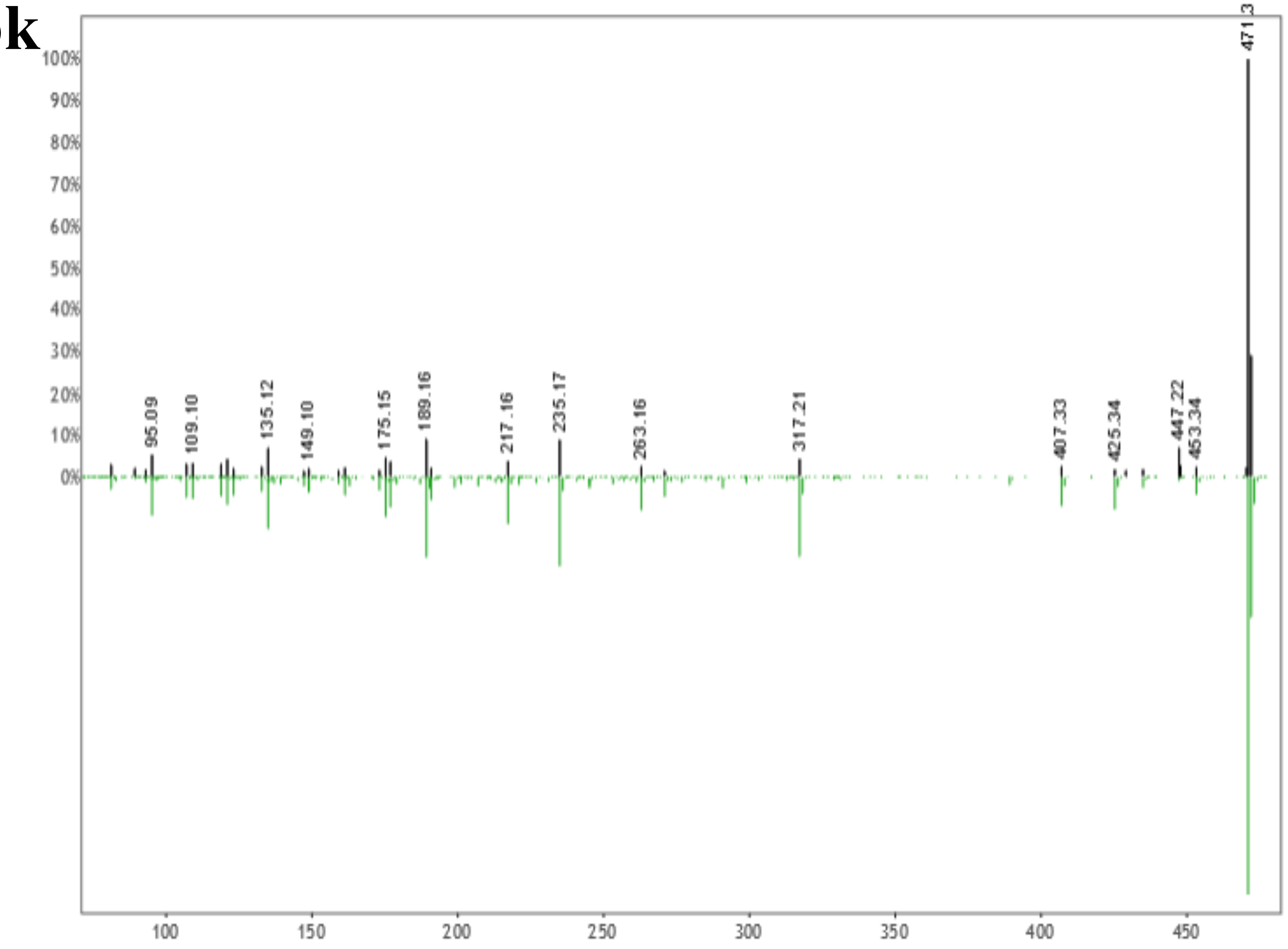
100%
90%
80%
70%
60%
50%
40%
30%
20%
10%
0%
95.09
109.10
135.12
149.10
175.15
189.16
217.16
235.17
263.16
317.21
407.33
425.34
447.22
453.34
471.3
100
150
200
250
300
350
400
450

bl

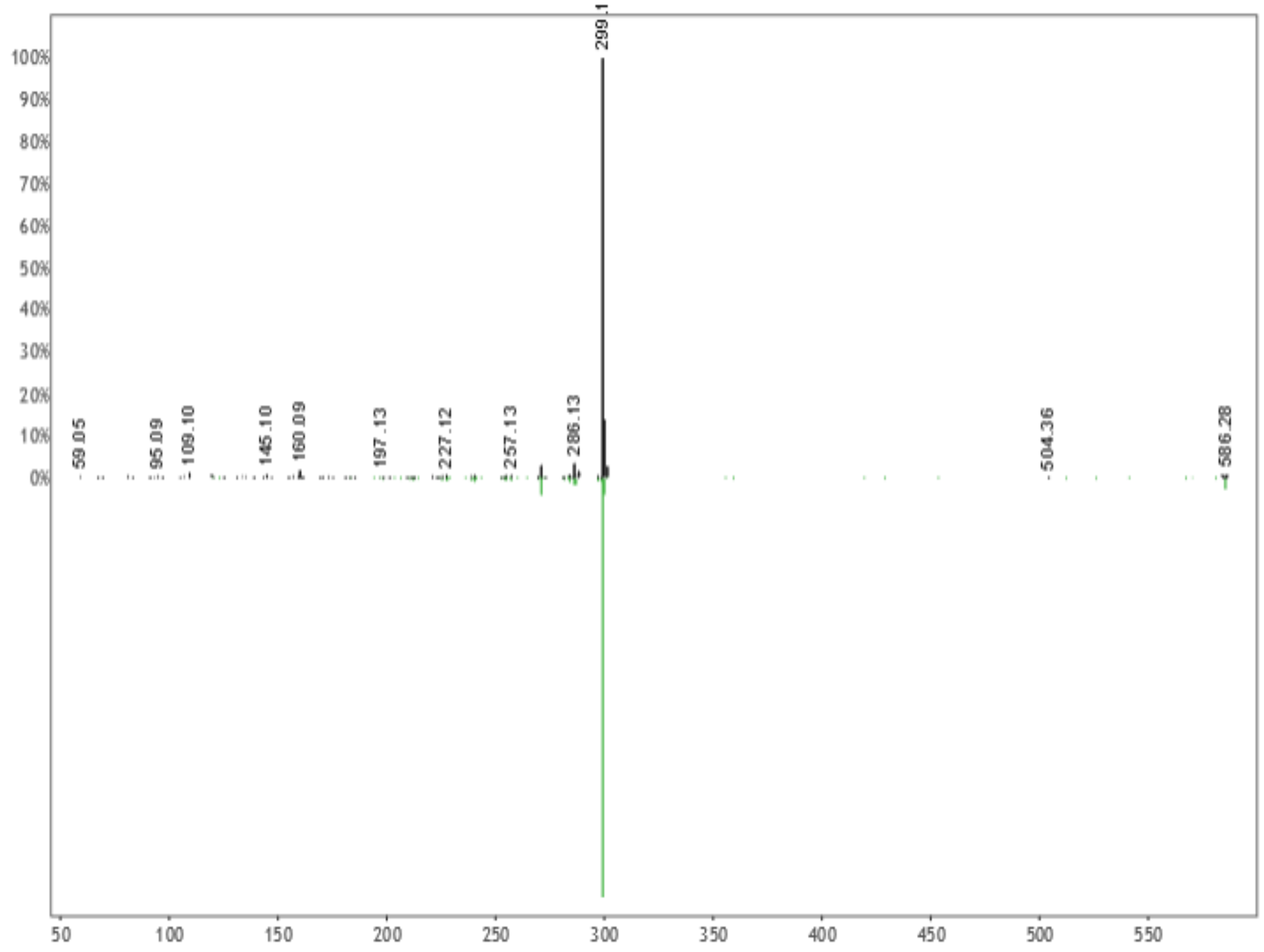
100%
90%
80%
70%
60%
50%
40%
30%
20%
10%
0%
59.05
95.09
109.10
145.10
160.09
197.13
227.12
257.13
286.13
299.1
504.36
586.28
50
100
150
200
250
300
350
400
450
500
550

bm

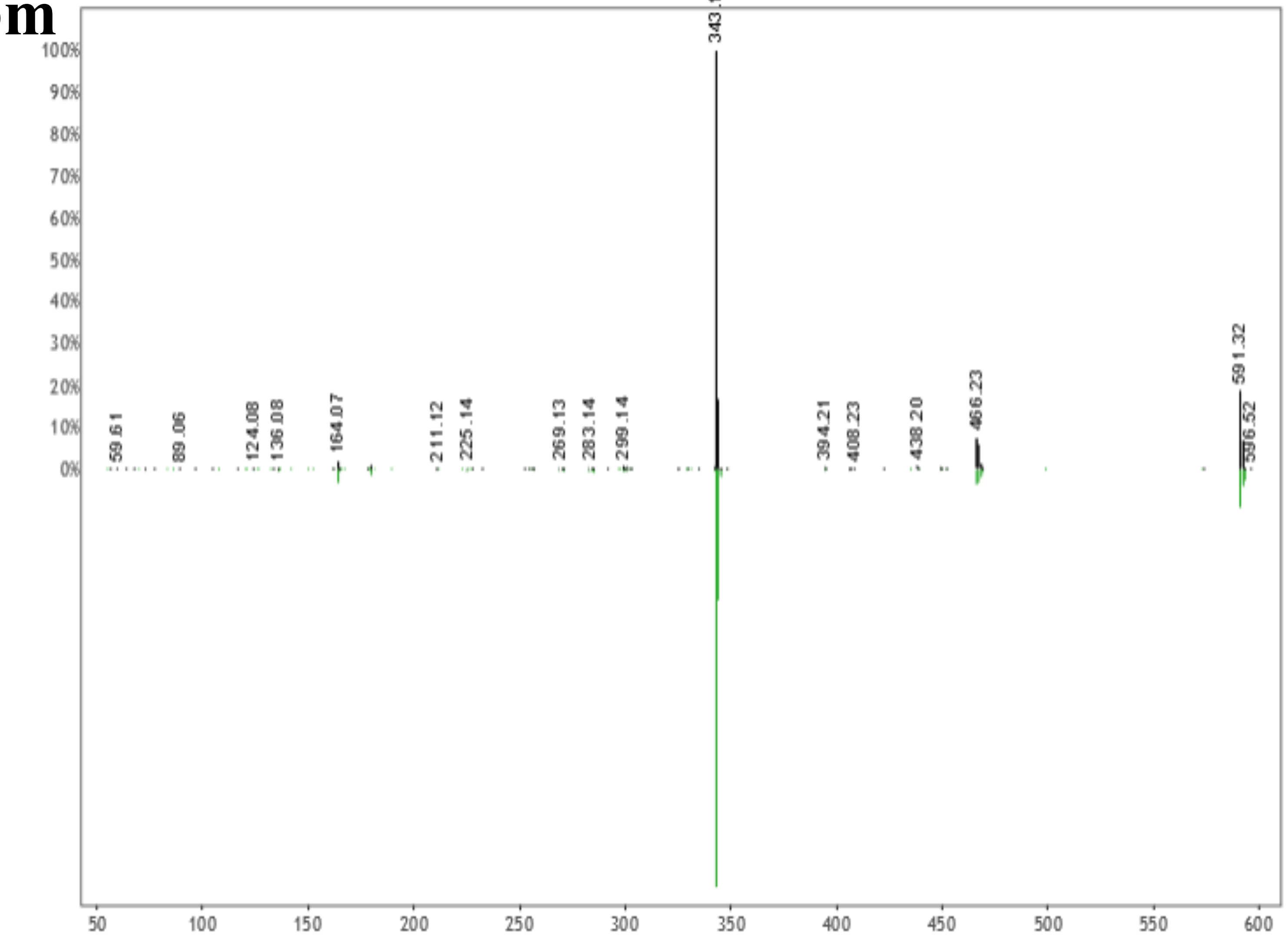
343.1
59.61
89.06
124.08
136.08
164.07
211.12
225.14
269.13
283.14
299.14
394.21
408.23
438.20
466.23
591.32
596.52

bn

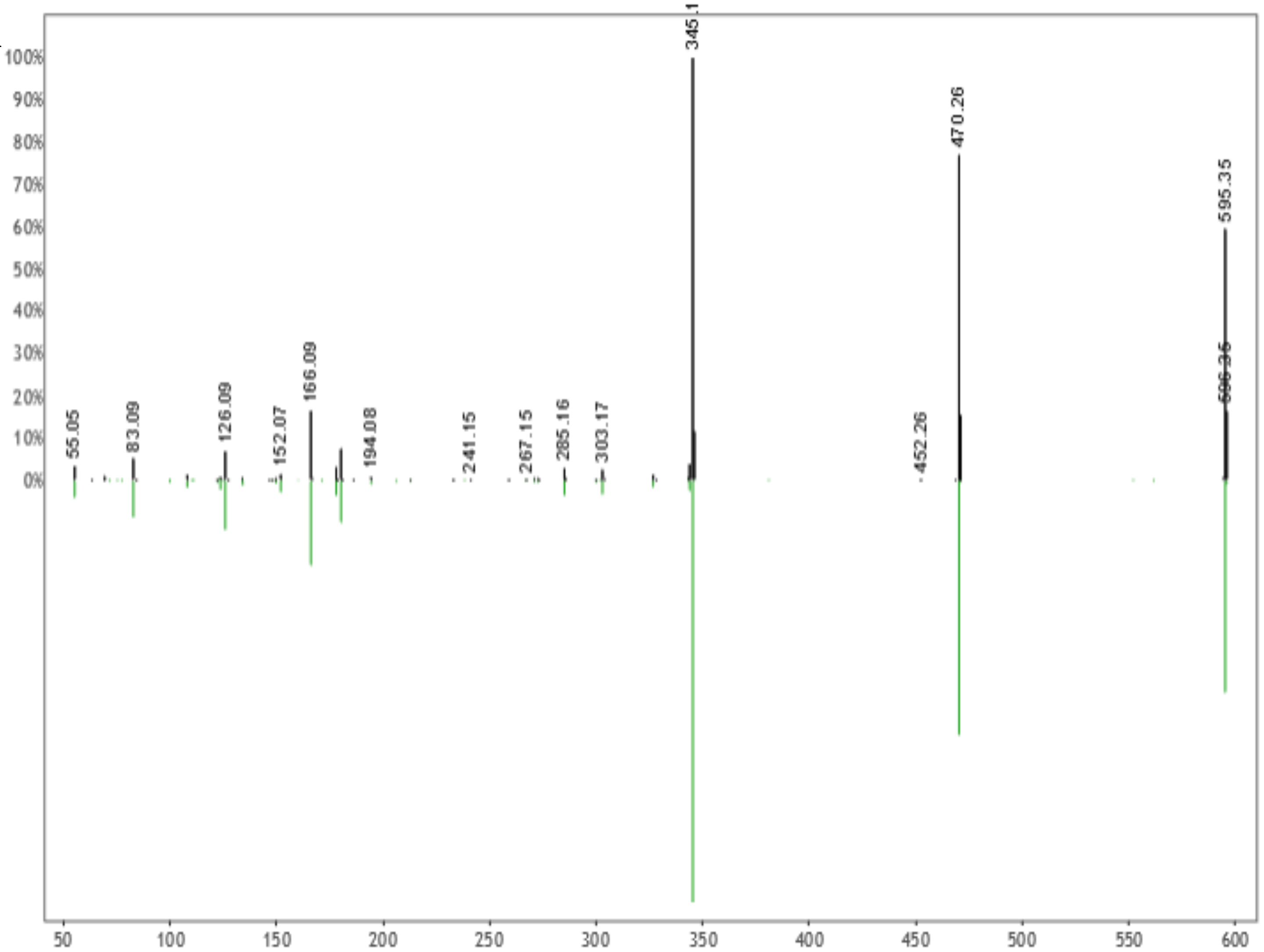
345.1
470.26
595.35
55.05
83.09
126.09
152.07
166.09
194.08
241.15
267.15
285.16
303.17
452.26

bo

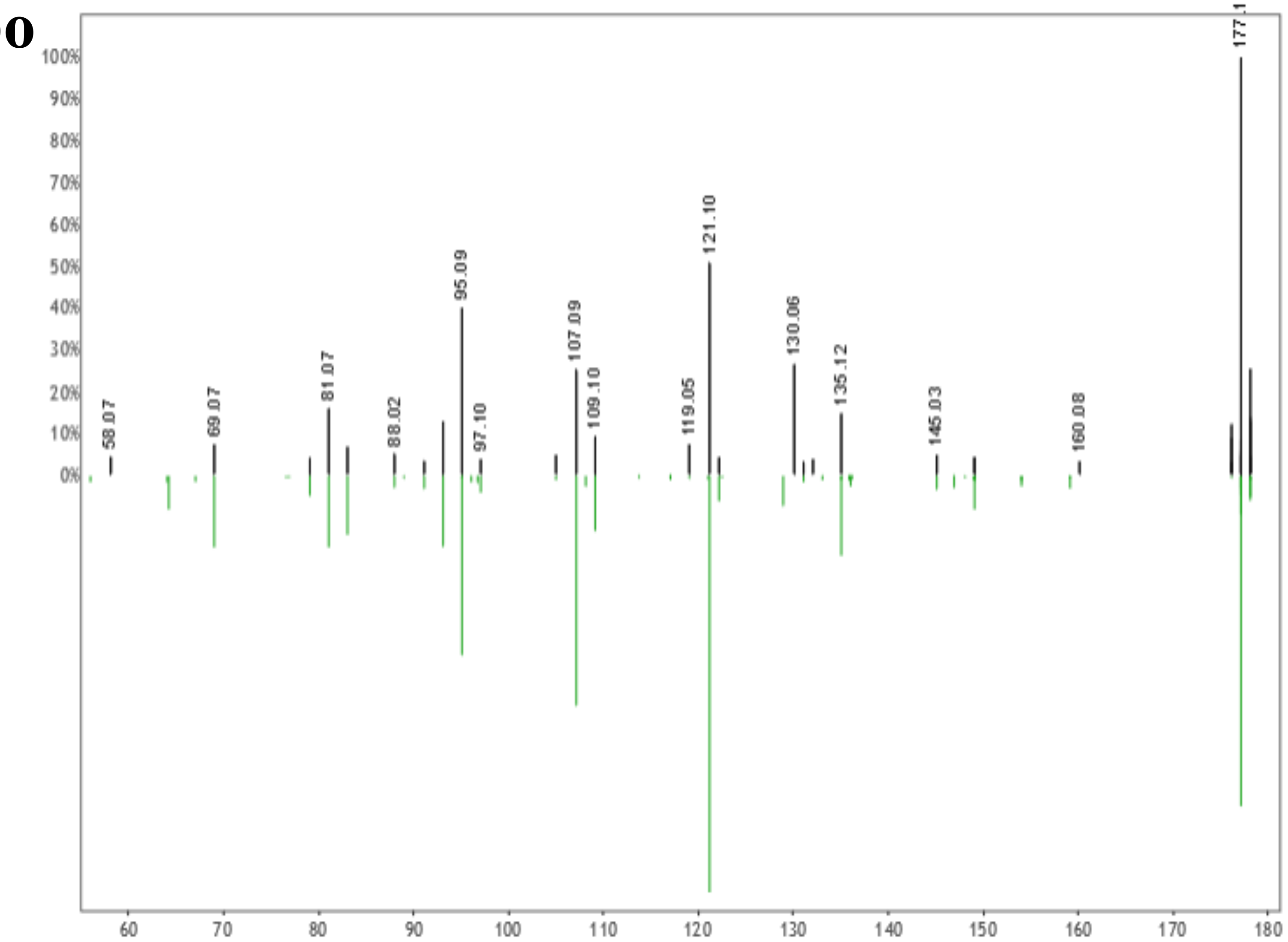
177.1
121.10
95.09
107.09
130.06
135.12
81.07
109.10
119.05
58.07
69.07
88.02
97.10
145.03
160.08
