# Supplementary material for: Untargeted Fecal Metabolomic Analyses across an Industrialization Gradient Reveal Shared Metabolites and Impact of Industrialization on Fecal Microbiome-Metabolome Interactions
Source: mSystems. 2022 Nov 23;7(6):e00710-22. doi: 10.1128/msystems.00710-22 (PMC9765122; doi:10.1128/msystems.00710-22)
Supplement: TABLE S3 [file msystems.00710-22-s0007.pdf]

**Compound Name**	***m/z***	**RT (min)**	**MASST Results**	**Common MASST Bacterial matches**	**Common MASST dataset matches**	**MASST Link**	**Present in ReDU All Human Fecal Sample Search**
Hypoxanthine	137.046	0.39	**5 dataset matches**; 1 human, 4 mouse	NA	NA	https://gnps.ucsd.edu/ProteoSAFe/status.jsp?task=4a2ea5870d2e4622a5b90e4edfca3f17	Present
Nicotinamide N-oxide	139.05	0.31	**5 dataset matches**; 1 human, 4 mouse	NA	NA	https://gnps.ucsd.edu/ProteoSAFe/status.jsp?task=6cd1ad150059472e84f3f95842588083	Absent
3-methyl-2-oxindole (3-Methyloxindole)	148.076	3.22	No results	NA	NA	https://gnps.ucsd.edu/ProteoSAFe/status.jsp?task=18ca57b672484cb2b4d89b5235afc96d	Present
Hyocholic acid	158.154	4.78	**12 dataset matches**; 1 human, 1 mouse, 3 environmental samples	*Pseudomonas, Streptomyces albus, Bacillus subtilis, E. coli, Bacteroides, Eubacterium, Akkermansia*	Human Gut Bacteria Biotransformation	https://gnps.ucsd.edu/ProteoSAFe/status.jsp?task=b93deedfe65c46de8b656c93f0e7f50d	Absent

Gly-Val (Glycylvaline)	175.107	0.36	**7 dataset matches**; 1 human, 1 environmental sample	*Pseudomonas*, *Streptomyces*, *Staphylococcus aureus*	Zoo mammal fecal metabolomes	https://gnps.ucsd.edu/ProteoSAFe/status.jsp?task=1ce565d3cbe842b1a3f3b82de47606a7	Present
3-Hydroxy-4-methoxycinnamic acid (Isoferulic acid)	177.055	4.13	**18 dataset matches**; 1 human, 5 mouse, 1 environmental sample, 4 plant (sweet orange, tomato), 1 mushroom dataset	"Bacteria"	Human-associated bacteria cultured with bile acids, Nobel twin study	https://gnps.ucsd.edu/ProteoSAFe/status.jsp?task=10153589289f45448d9db441d300369a	Absent
2-Butanone, 4-(2,6,6-trimethyl-2-cyclohexen-1-yl)	177.164	3.15	**39 dataset matches**; 6 human, 4 mouse, 5 rat, 4 environmental samples, 6 plant (rosemary, *Eudicotyledons*, thale cress, tomato)	*Marinobacter*, *Amycolatopsis*, *Streptomyces*	Burger ingredients common human diet, Amerindians urbanization gradient, zoo mammal fecal metabolomes, anti-inflammatory diet in rheumatoid arthritis patients	https://gnps.ucsd.edu/ProteoSAFe/status.jsp?task=d762844f0b444356a4958da4c610765f	Present

Paraxanthine	181.072	0.81	**14 dataset matches**; 11 human, 1 mouse, 1 environmental samples	NA	Anti-inflammatory diet in rheumatoid arthritis patients, Nobel twin study, Human rheumatoid arthritis, Metabolomic analysis of Alzheimer's disease	https://gnps.ucsd.edu/ProteoSAFe/status.jsp?task=fc336a940189429f85395db848e77d57	Present
trans-Ferulic acid	195.065	3.01	**52 dataset matches**; 11 human, 20 mouse, 1 rat, 1 environmental sample, plants (melon, corn, tomato, oranges), chemical standards with human samples	*Bacillus subtilis, Pseudomonas, Bacteroides, Bifidobacterium, Akkermansia*	Human Gut Bacteria Biotransformation	https://gnps.ucsd.edu/ProteoSAFe/status.jsp?task=bf38c3b5b06c440ba397bbc10cf73601	Present
Loliolide	197.117	3.11	**95 dataset matches**; 10 human, 4 mouse, 2 rat, 28 environmental samples, ~10 plants (thale cress, tomato, thapsia, melon, corn)	*Pseudomonas, Bacillus, Cyanobacteria, Streptomyces*	Zoo mammal fecal metabolomes, Amerindians urbanization gradient, ONR Human Wright Study, Analysis of Alzheimer's	https://gnps.ucsd.edu/ProteoSAFe/status.jsp?task=be50e86038794e1b9e0225109de958fa	Present

3-Hydroxydodecanoic acid	199.169	5.35	**21 dataset matches**; 13 human, 3 mouse, 1 environmental samples, 1 plant (corn), 1 food fermentation metagenome	*Bacillus subtilis, Burkholderia*	American Gut (with comparison for high and low plant consumption), Nobel twin study, Human fecal material standards, Human rheumatoid arthritis	https://gnps.ucsd.edu/ProteoSAFe/status.jsp?task=fb1b298fcd564e1594389d0207087f21	Present
N-Acetyl-D-mannosamine	204.087	3.27	**83 dataset matches**; 20 human, 11 mouse, 2 rat, 20 environmental samples, ~4 plant (tomato, cotton)	*Streptomyces albus, Pseudomonas, E. coli, C. difficile, Buckholderia, Actinomycete*	American Gut, Amerindians Urbanization Gradient, Human fecal characterization test, Human rheumatoid arthritis	https://gnps.ucsd.edu/ProteoSAFe/status.jsp?task=fc40869d5519485a808dc7caf0a2b8ae	Present
N-acetyl-L-Phenylalanine	208.097	2.84	**76 dataset matches**; 17 human, 15 mouse, 7 environmental samples, 8 food metagenome, 1 plant (tomato)	*Actinobacteria, Streptomyces, Rothia isolates, Bacillus, Akkermansia, Bifidobacterium, Pseudomonas, Cutibacterium acnes, Staphylococcus aureus*	American Gut (also with high and low plant consumption comparison), Amazon urbanization housing analysis, Nobel twin study, Human Gut Bacteria Biotransformation	https://gnps.ucsd.edu/ProteoSAFe/status.jsp?task=7279069e65014373ad790ecb80156672	Present

Thr-Pro (Threonylproline)	217.122	0.46	**15 dataset matches**; 5 human, 5 mouse, 1 rat, 1 environmental samples, 2 plants (tomato)	"Bacteria"	NA	https://gnps.ucsd.edu/ProteoSAFe/status.jsp?task=607f9ac84efe43a9a26a642389869975	Present
Val-Val (Valylvaline)	217.155	0.45	**35 dataset matches**; 6 human, 7 mouse, 4 rat, 2 environmental samples, 2 food metagenome, ~3 plant (thale cress, potato, wild oat)	*Staphylococcus aureus, Bacteroides, Akkermansia, Bifidobacterium*	Human gut bacteria biotransformation, Zoo mammal fecal metabolomes, Anti-inflammatory diet in rheumatoid arthritis patients, Human fecal material standards, Burger ingredients, ONR feces and plasma	https://gnps.ucsd.edu/ProteoSAFe/status.jsp?task=a0be1a5518ef4594bd188eed2bd4e6d2	Present

Abrine	219.113	0.61	**3 dataset matches**; 3 human	NA	American Gut (also UK), Malawi legume study	https://gnps.ucsd.edu/ProteoSAFe/status.jsp?task=7a23704e8e7c40a7a18692083e5b00c2	Present
Pantothenic acid	220.118	0.56	**142 dataset matches**; 33 human, 33 mouse, 4 rat, 5 environmental samples, ~11 plant (potato, peppers, melon, corn, sugarcane, tomato, thale cress, thapsia, *Nicotiana benthamiana*)	*Pseudomonas*, *E. coli*, *Streptomyces*, *Bacillus*, *Staphylococcus aureus*	American gut (also UK; also high vs low plant consumption comparison), Nobel twin study, Zoo mammal fecal samples, Human rheumatoid arthritis	https://gnps.ucsd.edu/ProteoSAFe/status.jsp?task=bce374bc20ef4f69ae9348a776d48e8f	Present

PyroGlu-Pro (Pyroglutamylproline)	227.103	0.44	**44 dataset matches**; 20 human, 5 mouse, 7 food metagenome, 2 environmental samples, ~3 plant (cotton, *Nicotiana benthamiana*, corn)	*Amycolatopsis*, *Bacillus subtilis*, *Pseudomonas*, *Streptomyces albus*	American Gut, ONR human diet, Human-associated bacteria cultured with bile acids	https://gnps.ucsd.edu/ProteoSAFe/status.jsp?task=893f768b31de46f5903ad003dfdb2152	Present
Myristoleic acid	227.201	5.94	**35 dataset matches**; 17 human, 6 mouse, 1 rat, 3 environmental samples, ~2 plant (cotton, tomato)	*Streptomyces chartreusis*	Burger ingredients, American gut (with high vs low plant consumption comparison), Amerindians urbanization gradient, ONR Primary Wright, Human fecal material standards, Alzheimer's analysis	https://gnps.ucsd.edu/ProteoSAFe/status.jsp?task=09d5bd98a46145e982b986a34e4591ef	Present

Ile-Pro (Isoleucylproline)	229.155	0.76	**168 dataset matches**; 44 human, 32 mouse, 5 rat, 12 environmental samples, 7 food metagenome, ~9 plant (tomato, corn, cotton, potato)	*Pseudomonas*, *Streptomyces*, *Bacillus*, *Bacteroides*, *Akkermansia*, *Bifidobacterium*, *Staphylococcus aureus*, *Lactobacillus*, *Amycolatopsis*, *Paenibacillus*, *E. coli*, *Eggerthella lenta*, *Serratia plymuthica*	American Gut (also UK and with plant consumption comparison), Human gut bacteria biotransformation, Human-associated bacteria cultured with bile acids, Zoo mammal fecal samples, Nobel twin study, Burger ingredients, Amerindians urbanization gradient, Human skin microbe isolates	https://gnps.ucsd.edu/ProteoSAFe/status.jsp?task=63a8a19454df4473b3bcbab0a4023ce9	Present
Val-Ile (Valylisoleucine)	231.171	2.82	**80 dataset matches**; 18 human, 11 mouse, 4 rat, 3 environmental samples, 6 food metagenome, ~6 plant (thale cress, *Nicotiana benthamiana*, tomato, cotton)	*Staphylococcus*, *Bacteroides*, *Akkermansia*, *Bifidobacterium*, *Pseudomonas*, *Actinomycete*, *Streptomyces*, *Alteromonas*	Human gut bacteria biotransformation, American gut (also UK), ONR Primary Wright, Burger ingredients, Human fecal material standards	https://gnps.ucsd.edu/ProteoSAFe/status.jsp?task=c96f435a91334e1fb38bcd1d0c111a11	Present

cis-9-Hexadecenoic acid (Palmitoleic acid)	237.221	6.49	**73 dataset matches**; 33 human, 14 mouse, 4 rat, 5 environmental samples (also built environment), ~6 plant (tomato, corn, sugarcane)	*Bacillus subtilis, Bacteroides, Bifidobacterium, Akkermansia, Lactobacillus, Cyanobacteria, Marinobacter, Prochlorococcus marinus, Rothia*	Human gut bacteria biotransformation, American Gut (subset for plant consumption comparison), ONR primary Wright, burger ingredients, Amerindians urbanization gradient, Nobel twin study	https://gnps.ucsd.edu/ProteoSAFe/status.jsp?task=d3eed7e162f24c3ab3b2c1c3e2e98785	Present
Gly-Tyr (Glycyltyrosine)	239.102	0.44	**34 dataset matches**; 13 human, 10 mouse, 1 rat, 3 environmental samples, ~2 plant (tomato, potato)	*Staphylococcus aureus, Bacteroides, Akkermansia, Bifidobacterium*	Human gut bacteria biotransformation, Human-associated bacteria cultured with bile acids, anti-inflammatory diet in rheumatoid arthritis patients	https://gnps.ucsd.edu/ProteoSAFe/status.jsp?task=ecdd1a94b14a4822a4466ca3c86b4bab	Present

Biotin	245.098	2.39	No results	NA	NA	https://gnps.ucsd.edu/ProteoSAFe/status.jsp?task=7e05ee9c085e492a871eb3bea1d24f50	Present
Leu-Leu (Leucylleucine)	245.186	2.40	**190 dataset matches**; 60 human, 30 mouse, 5 rat, 16 environment (also built environment), 13 food metagenome, ~14 plant (tomato, potato, melon, corn, malus, cotton, *Nicotiana benthamiana*, oranges)	*E. coli, Staphylococcus aureus, Pseudomonas, Streptomyces, Nocardia, Bacillus, Burkholderia, Eggerthella lenta, Bacteroides, Akkermansia, Bifidobacterium, Lactobacillus, Roseburia, Paenibacillus, Alteromonas, Amycolaptosis, Xanthomonas citri, Actinomycete*	American gut (with plant consumption comparison), Human gut bacteria biotransformation, Burger ingredients, Human-associated bacteria cultured with bile acids, ONR human diet, ONR primary wright, Human skin microbiome isolates, Amazon urbanization housing analysis, Zoo mammal fecal samples, Ancient DNA teeth, Anti-inflammatory diet	https://gnps.ucsd.edu/ProteoSAFe/status.jsp?task=5362cc2f00994eeba80d4bc3db7954e0	Present

					of rheumatoid arthritis patients, Amazon skin and environmental samples		
Lenticin	247.145	1.22	**42 dataset matches**; 35 human, 3 environmental samples, 1 plant (tomato)	NA	ONR Human study, Human rheumatoid arthritis, American gut (also UK), Zoo mammal fecal samples, Nobel twin study	https://gnps.ucsd.edu/ProteoSAFe/status.jsp?task=da61e8f7b5e744ac99a829e23a067c71	Present
Val-Met (Valylmethionine)	249.126	0.56	**27 dataset matches**; 4 human, 6 mouse, 5 rat, 3 food metagenome, ~2 plant (potato, tomato)	*Staphylococcus aureus*, *Bacteroides*, *Akkermansia*, *Bifidobacterium*	Human gut bacteria biotransformation, Burger ingredients, ONR human diet, Zoo mammal fecal samples, Anti-inflammatory diet in rheumatoid arthritis patients	https://gnps.ucsd.edu/ProteoSAFe/status.jsp?task=3772722082864e3db8ffa8be8d87607c	Present

Ser-Phe (Serylphenylalanine)	253.118	0.79	**64 dataset matches**; 18 human, 13 mouse, 2 rat, 9 environmental samples, ~6 plant (cotton, thale cress, potato, corn, tomato)	*Pseudomonas*, *Streptomyces*, *E. coli*, *Amycolaptosis*, *Bacillus*	Human gut bacteria biotransformation, American gut (with plant consumption comparison), Zoo mammal fecal samples, Burger ingredients, Nobel twin study	https://gnps.ucsd.edu/ProteoSAFe/status.jsp?task=4cd767ae2c9d4f2a815c2120511c9112	Present
Palmitelaidic acid	255.232	5.96	**31 dataset matches**; 12 human, 9 mouse, 1 rat, 3 environment (also built environment), ~1 plant (corn)	*Bacillus subtilis*	Amerindians urbanization gradient	https://gnps.ucsd.edu/ProteoSAFe/status.jsp?task=8dbc4bee89914f249f91ab103ac05d26	Present
L-Saccharopine	259.129	0.31	**15 dataset matches**; 5 human, 2 mouse, ~1 plant (tomato)	*Streptomyces clavuligerus*, *Streptomyces albus*	American and UK gut	https://gnps.ucsd.edu/ProteoSAFe/status.jsp?task=e4dd2264edc145dd9fd0525d9e8fbc4a	Present
Phe-Pro (Phenylalanylproline)	263.139	2.57	**196 dataset matches**; 62 human, 29 mouse, 5 rat, 18 environment (including built environment), 8 food	*Staphylococcus aureus*, *Bacteroides*, *Akkermansia*, *Bifidobacterium*, *Roseburia*, *Bifidobacterium*, *E. coli*, *Pseudomonas*, *Bacillus*, *Burkholderia*, *Amycolaptosis*, *Leptospira*, *Eggerthella lenta*,	Human gut bacteria biotransformation, American gut (with plant consumption comparison), Human-associated	https://gnps.ucsd.edu/ProteoSAFe/status.jsp?task=ae38f3f9e8864fb59edb943a8e514bae	Present

			metagenome, ~4 plant (cotton, sugarcane, tomato, corn)	*Actinomycete, Paenibacillus, Streptomyces, Acinetobacter pittii, Pantoea conspicua, Klebsiella, Enterobacter, Rothia, Veillonella atypica*	bacteria cultured with bile acids, Burger ingredients, Human skin microbiome isolates, Human rheumatoid arthritis, Zoo mammal fecal samples, Nobel twin study, Amazon skin and environmental samples		
Conjugated linoleic Acid (10E,12Z)	263.237	7.52	**132 dataset matches**; 43 human, 33 mouse, 6 rat, 10 environmental samples (including built environment), 14 food metagenome, ~6 plant (potato, corn, thale cress), ~5 animal (baboon, cheetah)	*Bacteroides, Akkermansia, Bifidobacterium, Roseburia, Eggerthella, Bacillus, Cutibacterium acnes*	Human gut bacteria biotransformation, American gut (with plant consumption comparison), Urbanization gradient foods, ONR wright human study, ONR primary wright, ONR human diet, Nobel twin study	https://gnps.ucsd.edu/ProteoSAFe/status.jsp?task=83c13baa8dc74e64b18653edd89e8567	Absent
Conjugated linoleic acid (9E,11E)	263.237	6.68	**144 dataset matches**; 48 human, 36 mouse, 6 rat, 11 environment (including built	*Bacteroides, Akkermansia, Bifidobacterium, Roseburia, Eggerthella, Methylobacter tundripaludum, Clostridium, Bacillus subtilis*	ONR Primary Wright Human, Human Gut Bacteria Biotransformation, ONR human diet,	https://gnps.ucsd.edu/ProteoSAFe/status.jsp?task=6389f1e6cf4f451	Present

			environment), 12 food metagenome, ~4 animal (baboon, rhino, cheetah), ~7 plant (corn, potato, thale cress, oranges)		American Gut (with plant consumption comparison), Amerindians urbanization gradient, Amazon urbanization housing analysis, Nobel twin study, Urbanization gradient foods	893599372f2b682b5	
Thr-Phe (Threonylphenylalanine)	267.134	0.48	**44 dataset matches**; 9 human, 10 mouse, 5 rat, 3 environment, ~4 plant (tomato, thale cress, potato), 3 animal (cheetah)	*Bacteroides, Akkermansia, Bifidobacterium, Staphylococcus aureus, Streptomyces*	Human Gut Bacteria Biotransformation, Amerindians urbanization gradient, Human rheumatoid arthritis, Burger ingredients, ONR wright human study, Zoo mammal fecal samples, Anti-inflammatory diet in rheumatoid arthritis patients	https://gnps.ucsd.edu/ProteoSAFe/status.jsp?task=34e92fc0e92349189ec8234819dac5d8	Present

Pro-Arg (Prolylarginine)	272.171	0.32	**7 dataset matches**; 4 human, 3 mouse	NA	NA	https://gnps.ucsd.edu/ProteoSAFe/status.jsp?task=4dd48325058045aa862fbb31c1a8230d	Present
9-OxoOTrE	275.201	4.42	**58 dataset matches**; 16 human, 8 mouse, 4 rat, 3 environment, 1 food metagenome, ~13 plant (corn, cotton, tomato, *Malpighiaceae* plants, melon, thale cress, potato, orange), ~1 other animal (rhino)	*Bacillus, Pseudomonas, Streptomyces scabiei*	Burger ingredients, ONR primary wright human study, Human rheumatoid arthritis, Zoo mammal fecal samples, Anti-inflammatory diet in rheumatoid arthritis patients	https://gnps.ucsd.edu/ProteoSAFe/status.jsp?task=9c0b6ad5c2f24f02b49c5d834598b39d	Present

Phe-Leu (Phenylalanylleucine)	279.171	2.59	**222 dataset matches**; 77 human, 37 mouse, 7 rat, 28 environment, 11 food metagenome, ~6 other animal (cheetah, rhino, cat), ~10 plant (cotton, *Malpighiaceae* plants, potato, tomato, thale cress, corn)	*Bacteroides, Akkermansia, Bifidobacterium, E. coli, Paenibacillus, Eggerthella lenta, Staphylococcus aureus, Pseudomonas, Nocardia, Streptomyces, Veillonella atypica, Achromobacter xylosoxidan, Cutibacterium acnes, Amycolaptosis*	Human gut bacteria biotransformation, American gut (with plant comparison and UK gut), Human-associated bacteria cultured with bile acids, Human skin microbiome isolates, Burger ingredients, Urbanization gradient foods, Amerindians urbanization gradient, Amazon urbanization housing analysis, Zoo mammal fecal samples, Ancient DNA teeth	https://gnps.ucsd.edu/ProteoSAFe/status.jsp?task=0506cb53c28b4aefa42281c3da9c0881	Present

Leu-Phe (Leucylphenylalanine)	279.171	3.47	**147 dataset matches**; 41 human, 26 mouse, 5 rat, 21 environment, 5 food metagenome, ~3 other animal (baboon, cheetah), ~9 plant (peppers, *Nicotiana benthamiana*, corn, potato, tomato, thale cress, cotton)	*Staphylococcus aureus*, *Eggerthella lenta*, *Akkermansia*, *Bacteroides*, *Bifidobacterium*, *Roseburia*, *Clostridium*, *Bacillus*, *Pseudomonas*, *Amycolaptosis*, *E. coli*, *Nocardia*, *Streptomyces*	Human gut bacteria biotransformation, ONR primary wright, Human-associated bacteria cultured with bile acids, Burger ingredients, Human rheumatoid arthritis, Amerindians urbanization gradient, American gut (with plant consumption comparison), Zoo mammal fecal samples, Ancient DNA teeth, Nobel twin study	https://gnps.ucsd.edu/ProteoSAFe/status.jsp?task=7781fd3196b142e0975dbafc0f737392	Present

N-Tetracosenoyl-4-sphingenine	282.279	6.09	**29 dataset matches**; 7 human, 11 mouse, 4 rat, 2 environment,	"Bacteria"	Burger ingredients, Human rheumatoid arthritis, Amerindians urbanization gradient, Anti-inflammatory diet in rheumatoid arthritis patients	https://gnps.ucsd.edu/ProteoSAFe/status.jsp?task=ca9486b15bc64efea62c56a21ff74057	Present
Octadecanamide	284.295	8.55	**5 dataset matches**; 3 human, 2 mouse	NA	ONR human plasma	https://gnps.ucsd.edu/ProteoSAFe/status.jsp?task=e11d31d2e9294d8ea30cf1712be3184a	Present

Xanthosine	285.083	0.40	**41 dataset matches**; 5 human, 21 mouse, 3 rat, 1 environment, 3 food metagenome, ~1 plant (*Genipapo*), ~1 other animal (rhino)	*Streptomyces, Pseudomonas fluorescens, Photobacterium galatheae, Paenibacillus peoriae, Xenorhabdus hominickii*	Burger ingredients, Zoo mammal fecal samples, Nobel twin study	https://gnps.ucsd.edu/ProteoSAFe/status.jsp?task=f40c0769edcc4b89b9e2f5507e2a976a	Present
Arg-Ile (Arginylisoleucine)	288.203	0.36	**53 dataset matches**; 9 human, 9 mouse, 5 rat, 5 environment, 6 food metagenome, ~3 plant (cotton, potato, tomato), ~1 other animal (rhino)	*Bacteroides, Akkermansia, Bifidobacterium, Roseburia, Clostridium, Paenibacillus, Amycolaptosis, Pseudomonas fluorescens, Photobacterium galatheae, Xenorhabdus hominickii*	Human gut bacteria biotransformation, Burger ingredients, Zoo mammal fecal samples	https://gnps.ucsd.edu/ProteoSAFe/status.jsp?task=ac1635b5525e4c48a6ba690f9bd314b1	Absent

cis-11,14-Eicosadienoic acid	291.268	5.54	**21 dataset matches**; 7 human, 2 mouse, 1 rat, 2 environment, 1 food metagenome, ~2 plant (Malpighiaceae plants, Myoporeae plants	NA	American Gut (with plant consumption comparison), Nobel twin study	https://gnps.ucsd.edu/ProteoSAFe/status.jsp?task=c7075cb219be444493020b722375a145	Absent
N-Acetylmuramic Acid	294.119	0.38	**17 dataset matches**; 1 human, 6 mouse, 1 rat, 2 environment, 1 plant (tomato)	*Streptomyces scabiei*, *Streptomyces*, *Streptomyces clavuligerus*	NA	https://gnps.ucsd.edu/ProteoSAFe/status.jsp?task=6c495d96e0bb4ceeb8c8821fd7a8f57b	Present

Tyr-Leu (Tyrosylleucine)	295.165	2.71	**153 dataset matches**; 48 human, 18 mouse, 6 rat, 16 environment, 12 food metagenome, ~3 other animal (cheetah, dog, rhino), ~6 plant (thale cress, melon, potato, corn)	*E. coli*, *Bacteroides*, *Akkermansia*, *Bifidobacterium*, *Roseburia*, *Eggerthella*, *Clostridium*, *Pseudomonas*, *Nocardia*, *Streptomyces*, *Bacillus*, *Amycolaptosis*, *Paenibacillus*, *Photobacterium galatheae*, *Xenorhabdus hominickii*	Human Gut bacteria biotransformation, ONR human diet, Human skin microbiome isolates, Burger ingredients, Human-associated bacteria cultured with bile acids, ONR primary wright, Amerindians urbanization gradient, Zoo mammal fecal samples, Nobel twin study, Anti-inflammatory diet in rheumatoid arthritis patients	https://gnps.ucsd.edu/ProteoSAFe/status.jsp?task=b55595173aa54b429e8056f941be4d3a	Present

Phe-Met (Phenylalanylmethionine)	297.126	2.18	**31 dataset matches**; 5 human, 7 mouse, 3 rat, 4 environment, 4 food metagenome, ~2 plant (tomato, corn)	*Bacillus*	ONR Wright human study, Amerindians urbanization gradient, Zoo mammal fecal samples	https://gnps.ucsd.edu/ProteoSAFe/status.jsp?task=f691a68d2503426aa15f80cdd92f7d90	Absent
Ile-Gly-Ile (Isoleucylglycylisoleucine)	302.205	3.04	**99 dataset matches**; 16 human, 29 mouse, 5 rat, 14 environment, 7 food metagenome, ~3 other animal (cheetah, cat, dog), ~5 plant (corn, tomato, potato)	*Eubacteria*, *Bacillus*, *Bacteroides*, *Akkermansia*, *Bifidobacterium*, *Roseburia*, *Eggerthella*, *Clostridium*, *Pseudomonas*, *Amycolaptosis*, *Streptomyces albus*, *Paenibacillus*	Burger ingredients, Human gut bacteria biotransformation, Human-associated bacteria cultured with bile acids, ONR human diet, Amerindians urbanization gradient, Zoo mammal fecal samples	https://gnps.ucsd.edu/ProteoSAFe/status.jsp?task=0b17a25c3d6445d5b2c4a8e335adba23	Present

Val-Trp (Valyltryptophan)	304.167	3.02	**28 dataset matches**; 2 human, 6 mouse, 2 rat, 2 food metagenome,~3 plant (tomato, cotton, potato), 1 other animal (rhino)	*Streptomyces clavuligerus*, *Streptomyces scabiei*, *Bacteroides*, *Akkermansia*, *Bifidobacterium*, *Roseburia*, *Eggerthella*, *Clostridium*	Human gut bacteria biotransformation, Zoo mammal fecal samples	https://gnps.ucsd.edu/ProteoSAFe/status.jsp?task=f2352760f6db441f9bb0880f3ee98c26	Present
Fructoselysine	309.164	0.31	**44 dataset matches**; 12 human, 13 mouse, 4 rat, 1 environment, 3 food metagenome	*Pseudomonas*, *Nocardia*, *Streptomyces*, *Bacteroides*, *Akkermansia*, *Bifidobacterium*, *Roseburia*, *Eggerthella*, *Clostridium*, *Amycolaptosis*	Human gut bacteria biotransformation, American gut (also UK)	https://gnps.ucsd.edu/ProteoSAFe/status.jsp?task=d3840c7b8fd0438ab2518ebbabbf5b62	Present
N-Palmitoylglycine	314.27	7.34	**8 dataset matches**; 1 human, 2 mouse, ~1 plant (*Euphorbiaceae*)	*Pseudomonas*, *Bacillus*	NA	https://gnps.ucsd.edu/ProteoSAFe/status.jsp?task=998632092bb7495692e01f7ba1be0248	Present

Ile-Trp (Iseoleucyltryptophan)	318.167	2.27	**59 dataset matches**; 7 human, 12 mouse, 4 rat, 5 environment, 2 food metagenome, ~5 plant (cotton, wild oat, corn, tomato, peppers), ~2 other animal (cheetah)	*Staphylococcus aureus, Bacillus, Streptomyces, Pseudomonas, Amycolaptosis, Bacteroides, Akkermansia, Bifidobacterium, E. coli, Eggerthella lenta*	Burger ingredients, Human gut bacteria biotransformation, Human-associated bacteria cultured with bile acids, ONR Wright human study, Zoo mammal fecal samples	https://gnps.ucsd.edu/ProteoSAFe/status.jsp?task=cf87c54ff6f14611846d4b9bb63f3b3b	Absent
Lithocholic acid	323.273	6.84	**17 dataset matches**; 8 human, 2 mouse, 3 rat, 1 environment, 1 plant (tomato)	NA	American Gut (also UK), ONR Primary Wright, Human rheumatoid arthritis, Zoo mammal fecal samples	https://gnps.ucsd.edu/ProteoSAFe/status.jsp?task=2bd41fcad2ef45be8edc72db760066f3	Present

Leucine enkephalin	336.192	3.22	**51 dataset matches**; 8 human, 10 mouse, 2 rat, 3 environment, 5 food metagenome, ~4 plant (corn, tomato), ~2 other animal (cheetah)	*Bacteroides, Akkermansia, Bifidobacterium, Eggerthella lenta, Roseburia, lactobacillus, Eubacterium, Bacillus, Streptomyces, Pseudomonas, Amycolaptosis, Paenibacillus*	Human gut bacteria biotransformation, Burger ingredients, ONR human diet, Human skin microbiome isolates, IBD severity human samples	https://gnps.ucsd.edu/ProteoSAFe/status.jsp?task=e0bbeeff3ae04f2191b9bedf2ba39a22	Absent

13-Docosenamide, (Z)- (Erucamide)	338.342	9.19	**228 dataset matches**; 52 human, 31 mouse, 6 rat, 24 environment, 5 food metagenome, ~10 other animal (baboon, cheetah, rhino, sheep), ~16 plant (*Mitragyna speciosa*, *Myoporeae* plants, *Angelica keiskei*, *Malpighiaceae* plants, *Thapsia garganica*, oranges, melon, tomato, wild oat, thale cress, *Psychotria insularum*, seaweed)	*Bacteroides*, *Akkermansia*, *Bifidobacterium*, *Roseburia*, *Eggerthella*, *Clostridium*, *Methylobacter tundripaludum*, *E. coli*, *Pseudomonas*, *Bacillus*, *Cyanobacteria*, *Colwellia chukchiensis*, *Maribacter ulvicola*, *Algoriphagus machipongonensis*, *Zobellia uliginosa*, *Saccharomonospora viridis*, *Burkholderia*, *Streptomyces*, *Moorena bouillonii*, *Glutamicibacter arilaitensis*, *Staphylococcus aureus*	Human gut bacteria biotransformation, Burger ingredients, ONR primary wright, Human-associated bacteria cultured with bile acids, Human skin microbiome isolates, ONR human fecal, American Gut (with plant consumption comparison), Amerindians urbanization gradient, ONR primary bile acids, Ancient DNA teeth	https://gnps.ucsd.edu/ProteoSAFe/status.jsp?task=50b42b413dbd4aa98745d64374ae8f6d	Present

Phe-Trp (Phenylalanyltryptophan)	352.166	3.28	**43 dataset matches**; 19 human, 11 mouse, 1 rat, 1 environment, ~2 other animal (baboon, cheetah), ~2 plant (tomato)	*Bacillus subtilis, Paenibacillus*	ONR Wright Human study, Burger ingredients, ONR primary wright, Zoo mammal fecal samples, Analysis of Alzheimer's, Nobel twin study	https://gnps.ucsd.edu/ProteoSAFe/status.jsp?task=ab7256224a5240c38e829d2455ec4738	Absent
Ile-Val-Lys (Isoleucylvalyllysine)	359.266	0.60	**13 dataset matches**; 1 human, 5 mouse, 3 rat,	*Pseudomonas, bacillus, Bacillus subtilis*	NA	https://gnps.ucsd.edu/ProteoSAFe/status.jsp?task=f1259278f31d49e7965135dc4fd92f5f	Present

Cholesterol	369.352	10.50	**204 dataset matches**; 102 human, 49 mouse, 6 rat, 5 environment, 17 food metagenome, ~5 plant (seaweed, tomato, sugarcane, thale cress, tomato), ~7 other animal (baboon, dog, sheep)	*Bacteroides, Bifidobacterium, Akkermansia, Cutibacterium acnes, Streptomyces coelicolor, Pseudomonas aeruginosa*	Amerindians urbanization gradient, Nobel twin study, American Gut (with plant consumption comparison and UK), Human gut bacteria biotransformation, urbanization gradient foods, ONR Wright human study, ONR human diet, ONR human fecal, Molecular analysis of human brain tissues	https://gnps.ucsd.edu/ProteoSAFe/status.jsp?task=4d622370a5fa4ad5bb1eab577469d246	Present

(R)-4-((3R,5S,8R,9S,10S,13R,14S,17R)-3-hydroxy-10,13-dimethyl-7,12-dioxohexadecahydro-1H-cyclopenta[a]phenanthren-17-yl)pentanoic acid	405.264	4.81	**51 dataset matches**; 22 human, 12 mouse, 1 rat, 1 environment, 5 food metagenome, ~3 other animal (cheetah)	*Streptomyces, Pseudonocardia, Gordonia, Kitasatospora*	Human-associated bacteria cultured with bile acids, IBD severity, American Gut (with plant consumption comparison), Zoo mammal fecal samples, Nobel twin study, IBD severity	https://gnps.ucsd.edu/ProteoSAFe/status.jsp?task=8cfb07ca20de46af9157341b39d25de5	Present
Glycoursodeoxycholic acid	414.301	5.01	**47 dataset matches**; 26 human, 2 mouse, 5 rat, 3 environment, ~2 other animal (baboon), ~1 plant (tomato)	*Bacteroides, Akkermansia, Bifidobacterium, Alteromonas, Pseudomonas fragi, E. coli, Streptomyces*	Human rheumatoid arthritis, ONR primary wright, Human gut bacteria biotransformation, Nobel twin study, Amerindians urbanization gradient, Analysis of Alzheimer's	https://gnps.ucsd.edu/ProteoSAFe/status.jsp?task=43e8d71f3ad84cc0b7a7967e821a45a9	Present

Cholic acid	426.318	5.19	**187 dataset matches**; 51 human, 54 mouse, 10 rat, 15 environment, 13 food metageneome, ~2 plant (Ardisia crenata, thale cress), ~6 other animal (baboon, dog, cheetah, sheep)	*Bacteroides, Akkermansia, Bifidobacterium, Roseburia, Eggerthella, Clostridium, Pseudomonas, Stenotrophomonas, Achromobacter, Salinispora, Photobacterium galatheae, Paenibacillus peoriae, Xenorhabdus hominickii, Streptomyces, Salinispora, Nocardiopsis, Brevibacterium, Bacillus, Alteromonas*	ONR Wright Human study, ONR Primary Wright, Burger ingredients, Human gut bacteria biotransformation, American Gut (also UK), Zoo mammal fecal samples, Nobel twin study, IBD severity	https://gnps.ucsd.edu/ProteoSAFe/status.jsp?task=496f2c33aaed4da0a9096623376f7afe	Present
6R)-2-(hydroxymethyl)-6-((3R,5R,7R,8R,9S,10S,12S,13R,14S,17R)-3,7,12-trihydroxy-10,13-dimethylhexadecahydro-1H-cyclopenta[a]phenan	431.318	5.25	**6 dataset matches**; 1 human, 2 mouse, 2 rat, 1 other animal (cheetah)	NA	Amerindians Urbanization gradient	https://gnps.ucsd.edu/ProteoSAFe/status.jsp?task=9d7b3242ba97467f88cc7e2849a73c9a	Absent

thren-17-yl)heptanoic acid							
Oleanolic acid	439.359	7.62	**106 dataset matches**; 22 human, 11 mouse, 3 rat, 5 environment, 16 food metagenome, ~5 other animal (cheetah, ant), ~11 plant (rosemary, *Fraxinus excelsior*, oranges, corn, tomato, *Parietaria judaica*)	*Streptomyces*, *Burkholderia*, *Bacillus*, *Eggerthella lenta*, *Cyanonacteria*, *Staphylococcus*, *Acinetobacter pittii*, *Pantoea conspicua*, *Klebsiella*, *Enterobacter*	ONR Wright Human study, American Gut, Amazon urbanization housing analysis, Urbanization gradient foods, Zoo mammal fecal samples	https://gnps.ucsd.edu/ProteoSAFe/status.jsp?task=800acf7db452461c9d0a890f990ecd83	Present

Glycocholic acid	446.32	4.69	**204 dataset matches**; 85 human, 19 mouse, 2 rat, 2 environment, 18 food metagenome, ~9 plant (corn, tomato, *Ardisia crenata*), ~7 other animal (rhino, baboon, cheetah)	*Cyanobacteria*, *Pseudomonas*, *Streptomyces*, *E. coli*, *Alteromonas*, *Bacillus subtilis*, *Pseudonocardia*, *Gordonia*, *Kitasatospora*, *Actinomadura*, *Nocardia*, *Actinomycetes*,*Pseudoaltero monas*, *Roseovarius*, *Staphylococcus aureus*, *Bacteroides*, *Akkermansia*, *Bifidobacterium*, *Roseburia*, *Eggerthella*, *Clostridium*, *Paenibacillus*, *Cutibacterium acnes*	Human-associated bacteria cultured with bile acids, ONR Wright human study, IBD human samples, Burger ingredients, Human rheumatoid arthritis, Human gut bacteria biotransformation, Amerindians urbanization gradient, Urbanization gradient foods, ONR human plasma, ONR primary bile acids, Nobel twin study, *Salinispora*, American Gut (with plant consumption comparison)	https://gnps.ucsd.edu/ProteoSAFe/status.jsp?task=8d5d343a95964faba3108020318837ff	Absent

Enoxolone	471.347	6.92	**32 dataset matches**; 12 human, 2 mouse, 2 environment, 4 food metagenome, ~2 other animal (cheetah), ~3 plant (melon)	*Bacillus subtilis, E. coli*	American Gut (also UK), Ancient DNA teeth	https://gnps.ucsd.edu/ProteoSAFe/status.jsp?task=bdc20710970b4c47a99c178a1c2606e9	Absent
Bilirubin	585.272	8.90	**123 dataset matches**; 65 human, 33 mouse, 7 rat, 1 environment, 1 food metagenome, ~5 other animal (cheetah, baboon, dog), ~2 plant (tomato, thale cress)	*Streptomyces albus, Kitasatospora cystarginea*	ONR human plasma, IBD human fecal samples, ONR Primary Wright, Human rheumatoid arthritis, American gut (with plant consumption comparison), Amerindians urbanization gradient, Amazon urbanization housing analysis, IBD severity	https://gnps.ucsd.edu/ProteoSAFe/status.jsp?task=2405d7eb22f64cceba64d6b43bd2f0aa	Present
Urobilin	591.318	4.07	**116 dataset matches**; 62 human, 34 mouse, 6 rat, 6 environment, ~4 other animal	NA	ONR Primary Wright, ONR Human Study, IBD severity, Human rheumatoid	https://gnps.ucsd.edu/ProteoSAFe/status.jsp?task=da0037fa7f4c4f7	Present

			(cheetah, cats, rhino), ~1 plant (tomato)		arthritis, American Gut (with plant consumption comparison), Zoo mammal fecal samples, IBD severity, Nobel twin study	2860d39a4d7184402	
Stercobilin	595.349	4.05	**94 dataset matches**; 57 human, 16 mouse, 6 rat, 6 environment, 2 food metagenome, ~4 other animal (rhino), ~2 plant (*Genipapo*, tomato)	NA	ONR human fecal, ONR Primary Wright, IBD severity, American gut (also UK), Amazon urbanization housing analysis, Amazon urbanization skin and environmental samples, Zoo mammal fecal samples, Nobel twin study, Anti-inflammatory diet of rheumatoid arthritis patients, Analysis of Alzheimer's disease	https://gnps.ucsd.edu/ProteoSAFe/status.jsp?task=e314b28d4ff343ccbef124eb09ffb016	Present
