# Supplementary material for: Untargeted Fecal Metabolomic Analyses across an Industrialization Gradient Reveal Shared Metabolites and Impact of Industrialization on Fecal Microbiome-Metabolome Interactions
Source: mSystems. 2022 Nov 23;7(6):e00710-22. doi: 10.1128/msystems.00710-22 (PMC9765122; doi:10.1128/msystems.00710-22)
Supplement: TABLE S4 [file msystems.00710-22-s0008.pdf]

MZmine v 2.33

Method	Parameter	Value
**Mass Detection**	MS1	1.80E+05
	MS2	1.00E+03
**Chromatogram Builder**	Min time span (min)	0.01
	Min height	5.40E+05
	m/z tolerance (ppm)	1.00E+01
	Filter	MS1
**Chromatogram Deconvolution - Local Minimum Search**	Chromatographic Threshold	5%
	Search minimum in RT range	0.10 min
	Min relative height	15%
	Min absolute height	3.00E+05
	Min ratio of peak top/edge	2
	Peak duration range	0.01-1.5min
	m/z range for MS2 scan pairing	0.01
	RT range for MS2 scan pairing	0.1min
**Isotopic Peaks Grouper**	*m/z* tolerance (ppm)	1.00E-02
	Retention time tolerance (min)	0.05
	Max charge	3
	Representative isotope	Lowest *m/z*
	Monotonic shape	Yes
**Join Aligner**	*m/z* tolerance (ppm)	10
	Weight for *m/z*	5
	Weight for RT	1
	Retention time tolerance (min)	0.5
**Peaks List Row Filter**	Retention time (min)	0.2-12.51
	Min peaks per row	empty (leave unchecked)
	Keep only peaks with MS2 scan (GNPS)	yes
**Gap Filling Peak Finder***	Intensity tolerance (%)	75
	m/z tolerance (ppm)	1.00E+01
	m/z tolerance (*m/z*)	1.00E-06
	Retention time tolerance (min)	0.3
	RT correction	yes
